# Theoretical investigation of the structural and spectroscopic properties of expanded metalloporphyrin complexes

**DOI:** 10.1098/rsos.181199

**Published:** 2019-01-23

**Authors:** Jie Jiang, Shengwei Guo, Xiaorong Wang, Liyan Xu, Qiang Li, Xiaoxin Zhang

**Affiliations:** 1 College of Chemistry, Chemical Engineering and Environmental Engineering, Liaoning Shihua University, Fushun, Liaoning 113001, People's Republic of China; 2 School of Materials Science and Engineering, North Minzu University, Yinchuan, Ningxia 750021, People's Republic of China

**Keywords:** porphyrin, benzoporphyrin, charge transfer, absorption spectra

## Abstract

The frontier molecular orbitals, UV–Vis absorption spectra, charge transfer (CT) and triplet excited states of 12 expanded D–A porphyrin/benzoporphyrin complexes were investigated using the density functional theory (DFT) method and time-dependent DFT in this work. The results showed that thiophene was an effective fragment for absorption of ‘long wavelength’, while the benzoporphyrin worked on the ‘short wavelength’, which was derived from its saddle-shaped structure; this expanded D–A conjugated system had a mild CT process with anthraquinone/isoindigo as acceptors and a strong CT process with naphtoquinone as acceptor. In addition, based on the simulation of the triplet state, the theoretical phosphorescence wavelength range of this series of derivatives was between 1000 and 1200 nm. This study is expected to assist the design of conjugated porphyrin for the field of porphyrin chemistry.

## 1. Introduction

Since the discovery of porphyrins (first reported by Woodward [1]) for the first time in the 1960s, porphyrin derivatives have attracted wide attention from researchers. Owing to the adjustability and diversity of porphyrin structures and their rich physical and chemical properties, researchers have applied them to the fields of sensors, biomimetics, catalytic chemistry, optical materials, molecular targeted drugs, solar cells and so on [2–7].

**Scheme 1.**
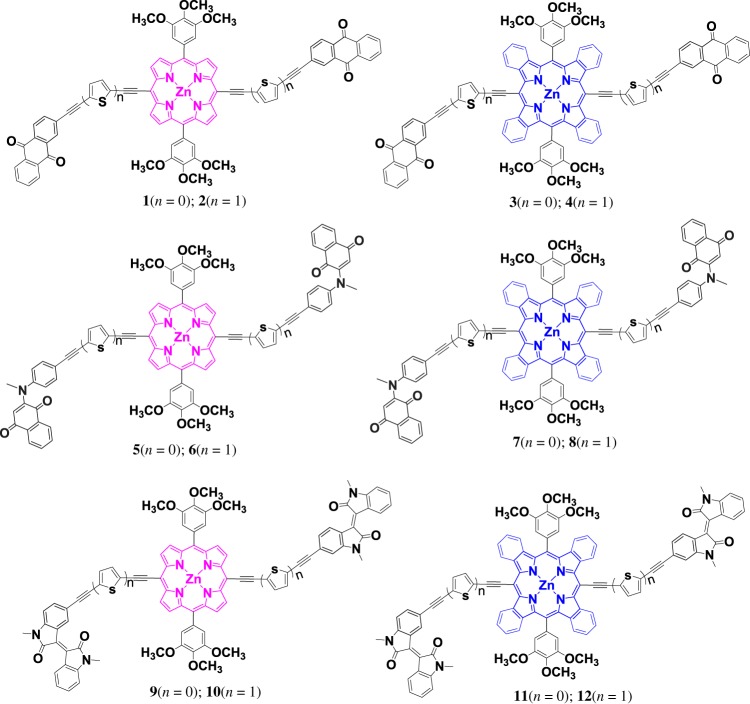
Structures of **1–12**.

The porphyrin ring with four pyrroles (shown in pink in scheme 1), as a conjugated system, is a common part of different porphyrin derivatives. Thus, different porphyrin derivatives have characteristic absorption in the UV–visible region, such as TPP (Tetraporphyrin): the simplest porphyrin with a strong narrow absorption peak (Soret band) near 420 nm and several weak absorption peaks (Q band) around 500–700 nm. In order to expand the light absorbing ability of porphyrin derivatives, donor–acceptor (D–A) strategy and increasing the degree of conjugation are usually adopted to make absorption spectrum red-shifted to 300 ∼ 550 nm, 600 ∼ 1000 nm [8].

In constructing the D–A large conjugated system, the following three methods are usually used: (1) thiophenes are widely used as one of the fragments by researchers because of their excellent electro-optical properties [9]; (2) by transforming different acceptors, A series of new D–A structures of porphyrin conjugated derivatives [8] were obtained; (3) by using benzoporphyrin (shown in blue in scheme 1) [10], it seems that the π-conjugated system is larger.

However, it is found that expanded D–A porphyrin complexes are difficult to synthesize, especially for the benzoporphyrin complexes. Up to now, there is little theoretical and experimental research on the expanded D–A benzoporphyrin complexes. In this work, we have investigated the frontier molecular orbitals, UV–Vis absorption spectra, charge transfer (CT) and triplet excited states of a series of unexploited expanded porphyrin/benzoporphyrin complexes which are D–A system (A–C≡C–[S]*_n_*–C≡C–[B]*_n_*–[Zn]–C≡C–[S]*_n_*–C≡C–A, *n* = 0 or 1, [S] = Thiophene, [B] = Benzo, [Zn] = zinc(II) porphyrin, as the donor unit; A = anthraquinone/naphtoquinone/isoindigo as acceptors). Twelve molecules were selected for systematic comparison shown in scheme 1. This work will provide valuable information and theoretical foundation for the spectroscopic properties of porphyrin derivatives.

## 2. Computational details

All calculations were performed with Gaussian 16 program package [11] at Liaoning Shihua University with supercomputer. The density functional theory (DFT) [12–15] and time-dependent DFT (TDDFT) [16–18] were calculated with the B3LYP [19–21] method while B3LYP functional has been widely used and comparatively accurate for porphyrin [22–27]. We also tried several DFT functionals but there was little difference for the ground state geometries. 6–31G* [28–33] basis sets were used for C, H, N and O atoms, and VDZ (valence double *ζ*) with SBKJC effective core potentials [34–36] were used for Zn atoms. Frequency calculations were also performed to make sure that the geometries of ground state reached the minimum point on the potential energy surfaces. The calculated absorption spectra and related MO contributions were obtained from the TDDFT/singlets output file and gaussum2.2. [37] The electrode potentials and orbital energies were obtained from the DFT calculations with a method similar to that used by Namazian [38]. The model compounds were optimized before the TDDFT calculations. Only the relevant (stronger oscillator strength and wave function coefficients) molecular orbitals are shown. All computations were performed in THF environment without symmetry constraints. The predicted phosphorescence wavelengths were obtained by calculating the difference in the total energy between the optimized triplet state (*T*_1_) and the optimized singlet state (*S*_0_). This energy difference is equated to the *T*_1_–*S*_0_ gap and can therefore be used to predict the phosphorescence wavelength of a compound [39].

## 3. Results and discussion

### 3.1. Optimized geometric structures

The geometrically optimized structures of the porphyrin derivatives **1–12** are shown in the electronic supplementary material. **1** and **2** exhibit good planarity. The dihedral angle formed between porphyrin ring and anthraquinone is close to 0°. Although a good planar structure is essential for communication and **5/6** is non-planar, electronic communication can also occur in the bridging NMe unit [40]. The isoindigo used in **9** and **10** is good acceptor. The dihedral angle between porphyrin ring and isoindigo is small (less than 20°C), which denotes their good planarity and excellent electron delocalization properties.

The common parent ring of **3–4**, **7–8**, **11–12**, benzoporphyrin, is saddle-shaped (the two corresponding ends are facing up, and the other two are facing down). The benzoporphyrin parent ring is not on a horizontal surface, resulting in an overall no longer planar structure, which is not conducive to the delocalization of electrons.

**Figure 1.**
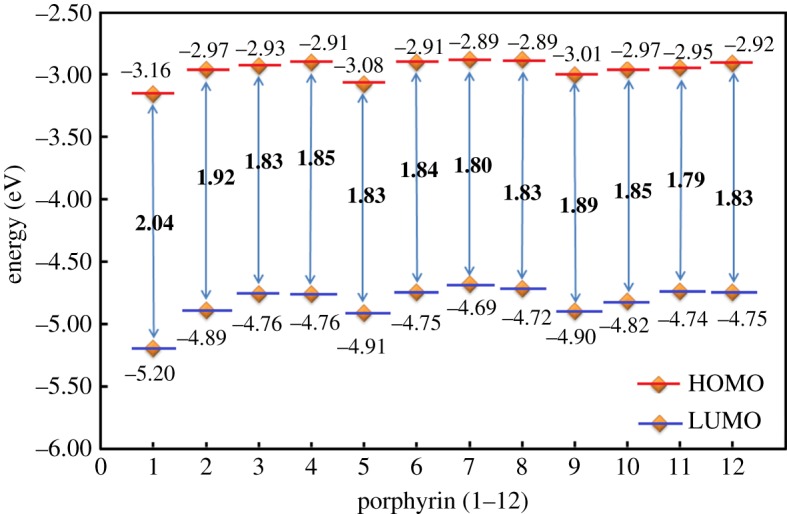
Illustration of the HOMOs and LUMOs for **1–12**.

The energy levels of the highest occupied molecular orbital (HOMO) and the lowest unoccupied orbital (LUMO) of **1–12** are shown in figure 1. With the addition of thiophene and the use of benzoporphyrin, the energy level difference between HOMO and LUMO is decreasing, **3** (1.83) ∼ **4** (1.85) < **2** (1.92) < **1** (2.04); **7** (1.80) <**8** (1.83) ∼ **5** (1.83) ∼ **6** (1.84); **11** (1.79) < **12** (1.83) ∼ **10** (1.85) < **9** (1.89). It is inferred that the electrons are more likely to transit from HOMO to LUMO for a smaller gap and their corresponding absorption spectrum is more red-shifted; the ability to absorb visible light will also be enhanced. Although both the thiophene and benzoporphyrin can make the HOMO-LUMO gap narrow, the change is very small for **5–8** using naphtoquinone as acceptor. This result indicates that it is necessary to take into account the collocation between the acceptor and other fragments in design.

### 3.2. Optical absorption properties

The classic UV–Vis absorption spectra of D–A porphyrins can be divided into two characteristic absorption bands: the first band around 350 ∼ 550 nm assigned to *S*_0_ → *S*_2_ transition, and the second band (called CT band) of 600 ∼ 1000 nm from *S*_0_ → *S*_1_ transition. A band that is neither present in the spectra of the isolated donor nor in the acceptor is called a CT band and represents evidence for interactions between the donor and acceptor, and consequently witnesses the electronic communication across the backbone of the porphyrin complexes. The presence of CT bands always arises from molecular orbital (MO) overlaps through π-conjugation namely (but not exclusively). The energy gap is much smaller and the CT band is red-shifted. And it can be explained by a simple molecular orbital theory concept [41].

**Figure 2.**
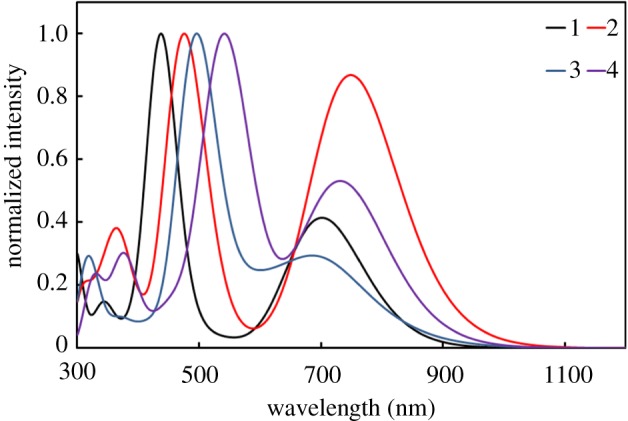
Simulated absorption spectra of **1–4**.

**Figure 3.**
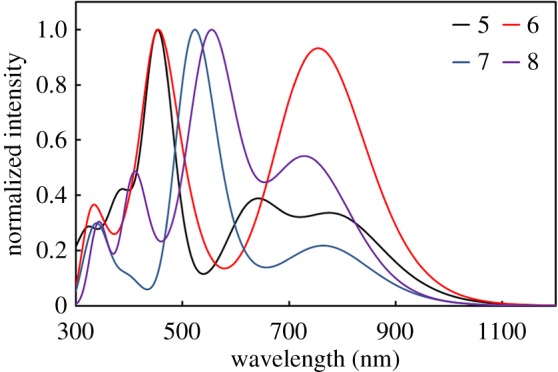
Simulated absorption spectra of **5–8**.

**Figure 4.**
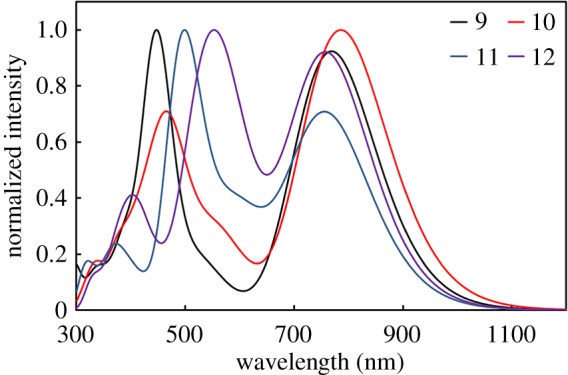
Simulated absorption spectra of **9–12**.

According to figures 2–4, all the *λ*_abs_ and main configuration of **1–12** show typical absorption characteristic of metalloporphyrins in the ultraviolet–visible (UV–Vis) region. The light-absorption ability and energy levels of porphyrin complexes can be modulated using different combinations of donor and acceptor units and conjugated strategy. Usually, expanded D–A conjugated systems are developed to possess broad and strong optical absorption, which can capture the solar irradiation as much as possible.

According to figure 2, with the insertion of thiophene in **2**, the spectra undergo significant red-shift and broadening, from the initial 350 ∼ 500 nm, 600 ∼ 900 nm to 400 ∼ 550 nm, 600 ∼ 1000 nm, and the relative absorption intensity of CT band has a very obvious increase, indicating that the addition of thiophene is very beneficial to the red-shift and broadening of the absorption spectrum. Compared with **1**, with the use of benzoporphyrin in the parent ring of **3**, the overall absorption spectrum did not have an obvious red-shift; only the Soret band was red-shifted to approximately 50 nm, indicating that the benzoporphyrin ring did not effectively strengthen the actual degree of CT. The ring of benzoporphyrin presents a saddle shape, resulting in a non-planar overall structure and the degree of overall conjugation is weakened. On the basis of the structure of **3**, the absorption spectrum of **4** is red-shifted by approximately 50 nm after the insertion of thiophene, further indicating that the thiophene fragment is favourable for the absorption of long-wavelength light.

Similarly, as shown in figure 3, the relative intensity of CT band of **6, 8** shows an obvious increase compared with that of **5, 7**, respectively, these absorption characteristics exactly come from the contribution of thiophene fragment in the structures of **6** and **8**. The Soret band of **7**, **8** shows a red-shift (68 nm, 100 nm) compared with **5**, **6**. However, the relative intensity of CT band of **7**, **8** is weaker than that of **5**, **6**, respectively. Therefore, it is clear that benzoporphyrin mainly acts on the Soret band that is typical *π* → π* transition of metal porphyrin, with no effect on the CT band.

As can be seen from figure 4, the profiles of the absorption spectra of **9–12** here are also similar to that discussed in the preceding paragraphs. We find that **10**, **12** has a red-shifted absorption spectrum relative to **9**, **11,** respectively. Thus, for the porphyrin complexes, the thiophene fragment is a more efficient group for improving the light-harvesting properties of long wavelength. By using benzoporphyrin instead of porphyrin ring, **11**, **12** shows a 50 nm, 90 nm red-shift of Soret band compared with **9**, **10**, respectively, which leads to a broad spectrum in the short visible region (450–600 nm).

### 3.3. Charge transfer

The representation of selected frontier MOs (HOMO−4; HOMO−3; HOMO−2; HOMO−1; HOMO; LUMO; LUMO+1; LUMO+2; LUMO+3; LUMO+4) for **1**–**12**, their normalized distributions of the fragment orbital contributions, selected calculated positions of the pure electronic transitions, oscillator strengths (*f*) and their major contributions are provided in tables 1 and 2. A bar graph reporting *f* as a function of wavelength is shown in the electronic supplementary material. By applying an arbitrary thickness of 1000 cm^−1^ to the bar graph (blue) of the 100 first electronic transitions, calculated spectra are generated (figures 2–4). CT feature normally detected greater than 700 nm is noted in table 1. For **1–12**, the CT processes are obvious computationally.

**Table 1.** Selected calculated positions of the pure electronic transitions, oscillator strengths (*f*) and major contributions^a^.

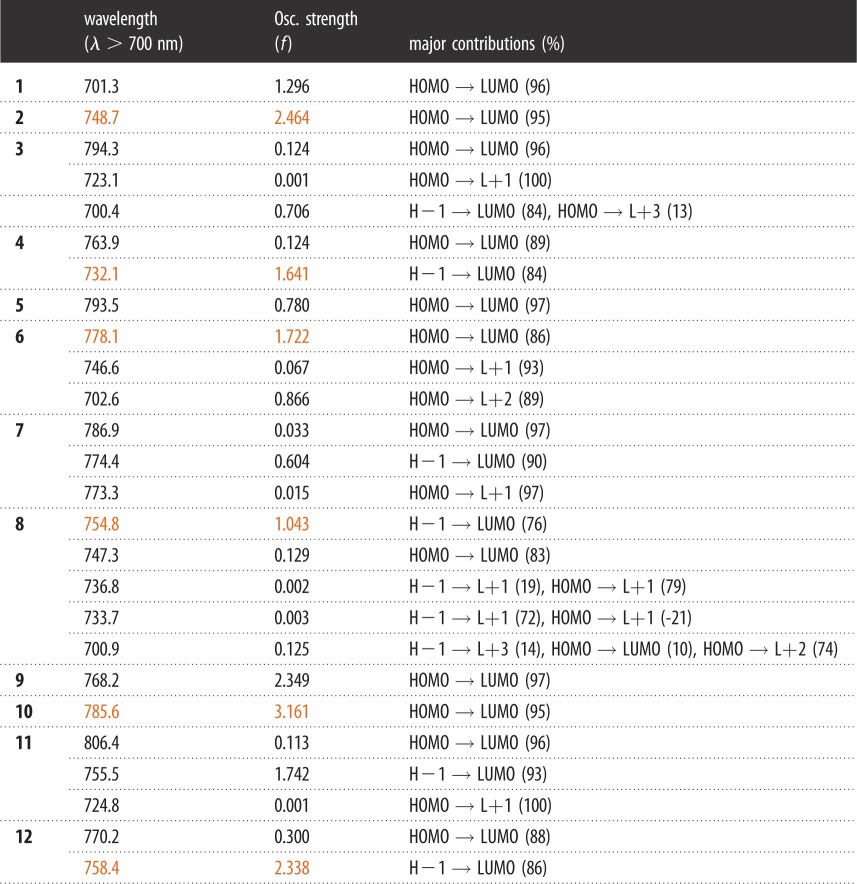

^a^The 100th transition is shown in the electronic supplementary material.

**Table 2.** Normalized distributions of the fragment orbital contributions of the frontier MOs for **1–12**, where the major contributions are in bold.

	H−4	H−3	H−2	H−1	H	L	L + 1	L + 2	L + 3	L + 4
**1**										
C≡C–[Zn]–C≡C	**0.76**	**0.99**	**0.99**	**∼1.00**	**0.88**	0.40	0.05	0.48	∼**1.00**	0.19
anthraquinone	0.24	0.01	0.01	∼0.0	0.12	**0.60**	**0.95**	**0.52**	∼0.0	**0.81**
**2**										
C≡C–[S]–C≡C–[Zn]–C≡C–[S]–C≡C	**∼1.00**	**0.96**	**0.86**	**∼1.00**	**0.96**	**0.60**	0.09	0.43	**∼1.00**	**0.61**
anthraquinone	∼0.0	0.04	0.14	∼0.0	0.04	0.40	**0.91**	**0.57**	∼0.0	0.39
**3**										
C≡C–[B-Zn]–C≡C	**0.81**	**0.99**	**0.98**	**0.89**	**0.99**	0.37	0.06	**0.56**	**0.99**	0.24
anthraquinone	0.19	0.01	0.02	0.11	0.01	**0.63**	**0.94**	0.44	0.01	**0.76**
**4**										
C≡C–[S]–C≡C–[B-Zn]–C≡C–[S]–C≡C	**∼1.00**	**0.96**	**0.88**	**0.96**	**∼1.00**	0.42	0.10	**0.63**	**∼1.00**	**0.62**
anthraquinone	∼0.0	0.04	0.12	0.04	∼0.0	**0.58**	**0.90**	0.37	∼0.0	0.38
**5**										
C≡C–[Zn]–C≡C	**1.00**	0.39	0.19	**∼1.00**	**0.72**	0.07	0.01	**0.80**	**∼1.00**	**0.52**
benzene-N-naphtoquinone	0.00	**0.61**	**0.81**	∼0.00	0.28	**0.93**	**0.99**	0.20	∼0.00	0.48
**6**										
C≡C–[S]–C≡C–[Zn]–C≡C–[S]–C≡C	**0.97**	0.40	**∼1.00**	0.47	**0.87**	0.16	0.02	**0.81**	**∼1.00**	**0.81**
benzene-N-naphtoquinone	0.03	**0.60**	∼0.0	**0.53**	0.13	**0.84**	**0.98**	0.19	∼0.0	0.19
**7**										
C≡C–[B-Zn]–C≡C	**∼1.00**	0.43	0.29	**0.74**	**0.99**	0.06	0.01	**0.85**	**0.99**	**0.61**
benzene-N-naphtoquinone	∼0.0	**0.57**	**0.71**	0.26	0.01	**0.94**	**0.99**	0.15	0.01	0.39
**8**										
C≡C–[S]–C≡C–[B-Zn]–C≡C–[S]–C≡C	**0.89**	0.44	**0.52**	**0.88**	**∼1.00**	0.06	0.02	**0.92**	**∼1.00**	**0.81**
benzene-N-naphtoquinone	0.11	**0.56**	0.48	0.12	**∼0.0**	**0.94**	**0.98**	0.08	∼0.0	0.19
**9**										
C≡C–[Zn]–C≡C	0.43	0.19	0.14	**∼1.00**	**0.74**	0.40	0.07	**0.54**	**∼1.00**	**0.78**
isoindigo	**0.57**	**0.81**	**0.86**	∼0.0	0.26	**0.60**	**0.93**	0.46	∼0.0	0.22
**10**										
C≡C–[S]–C≡C–[Zn]–C≡C–[S]–C≡C	0.23	0.28	**∼1.00**	0.46	**0.90**	**0.52**	0.12	**0.54**	**∼1.00**	**0.82**
isoindigo	**0.77**	**0.72**	∼0.0	**0.54**	0.10	0.48	**0.88**	0.46	∼0.0	0.18
**11**										
C≡C–[B-Zn]–C≡C	**0.55**	0.21	0.22	**0.76**	**0.99**	0.34	0.08	**0.65**	**0.99**	**0.77**
isoindigo	0.45	**0.79**	**0.78**	0.24	0.01	**0.66**	**0.92**	0.35	0.01	0.23
**12**										
C≡C–[S]–C≡C–[B-Zn]–C≡C–[S]–C≡C	0.29	0.31	**0.52**	**0.91**	**∼1.00**	0.38	0.12	**0.70**	**∼1.00**	**0.82**
isoindigo	**0.71**	**0.69**	0.48	0.09	∼0.0	**0.62**	**0.88**	0.30	∼0.0	0.18

Time-dependent DFT (TDDFT) calculations (table 1) place the lowest electronic transitions at 701.3 nm (H → L (96%), 1.293) for **1**, 748.7 nm (H → L (95%), 2.464) for **2**, 794.3 nm (H → L (96%), 0.124) for **3**, 763.9 nm (H → L (89%), 0.124) for **4**, 793.5 nm (H → L (97%), 0.780) for **5**, 778.1 nm (H → L (86%), 1.722) for **6**, 786.9 nm (H → L (97%), 0.033) for **7**, 754.8 nm (H−1 → L (76%), 1.043) for **8**, 768.2 nm (H → L (97%), 2.349) for **9**, 785.6 nm (H → L (95%), 3.161) for **10**, 806.4 nm (H → L (96%), 0.113) for **11**, 770.2 nm (H → L (88%), 0.300) for **12**, respectively. Although the longest wavelength does not appear to be regular, the strongest *f* occurs at **2**, **4**, **6**, **8**, **10**, **12** owing to the thiophene fragment, which can further explain that [S] is beneficial for the CT process (orange, table 1).

The atomic orbital contributions of the HOMOs are mainly composed of the C≡C–[S]_n_–C≡C–[B]_n_–[Zn]–C≡C–[S]_n_–C≡Cπ-system for **1–4** (88%; 96%; 99%; approx. 100%); conversely, the LUMO exhibits atomic orbital contributions located on the π-systems of the anthraquinones (60%; 40%; 63%; 58% for **1–4**, respectively). The remainder is located on the C≡C–[S]_n_–C≡C–[B]_n_–[Zn]–C≡C–[S]_n_–C≡C π-system. The HOMO → LUMO transitions are consistent with the expected CT processes C≡C–[S]_n_–C≡C–[B]_n_–[Zn]–C≡C–[S]_n_–C≡C → AQ. This distribution for the C≡C–[S]_n_–C≡C–[B]_n_–[Zn]–C≡C–[S]_n_–C≡C/anthraquinone fragments makes this CT process rather moderate.

For **5–8** series, DFT computations indicate that most of the atomic orbital contributions of the HOMOs are composed of the C≡C–[S]_n_–C≡C–[B]_n_–[Zn]–C≡C–[S]_n_–C≡Cπ-system (72%; 87%; 99%; approx. 100%), whereas the LUMO is composed only of the naphtoquinone (93%; 84%; 94%; 94% for **5–8**, respectively). This distribution for the C≡C–[S]_n_–C≡C–[B]_n_–[Zn]–C≡C–[S]_n_–C≡C/naphtoquinone fragments makes this CT process (C≡C–[S]_n_–C≡C–[B]_n_–[Zn]–C≡C–[S]_n_–C≡C → NQ) stronger compared to that of **1–4** series.

An exactly similar situation is observed for the HOMOs of **9–12** where the atomic contributions are most computed on the C≡C–[S]_n_–C≡C–[B]_n_–[Zn]–C≡C–[S]_n_–C≡Cπ-system (74%; 90%; 99%; approx. 100%), while the LUMO exhibits atomic contributions, respectively, centred on the π-systems of the isoindigo (60%; 48%; 66%; 62% for **9–12**). Electronic transitions between HOMO → LUMO lead to a moderate CT process.

### 3.4. Triplet state

The transition metal [Zn] used in **1–12** makes the original forbidden triplet transition possible by spin–orbit coupling effect, suggesting the possible presence of phosphorescence. The total energy difference between the lowest energy triplet excited state and the ground state is calculated to predict the phosphorescence wavelength by DFT. As a result, the theoretical phosphorescence wavelengths of **1–12** are between 1000 and 1200 nm (table 3).

**Table 3.** Predicted position (from DFT computations) of the phosphorescence 0–0 Peaks.

	*T*_1_–*S*_0_ energy gap		
compounds	Hartree	eV	calculated *T*_1_ → *S*_0_ wavelength (nm)
**1**	0.04482	1.22	1017
**2**	0.04089	1.11	1114
**3**	0.03892	1.06	1171
**4**	0.04433	1.21	1028
**5**	0.04411	1.20	1033
**6**	0.04054	1.10	1124
**7**	0.04204	1.14	1084
**8**	0.03971	1.08	1148
**9**	0.04127	1.12	1104
**10**	0.04040	1.10	1128
**11**	0.03777	1.03	1206
**12**	0.03846	1.05	1185

## 4. Conclusion

In summary, the frontier molecular orbitals, UV–Vis absorption spectra, CT and triplet excited states of a series of expanded D–A porphyrin/benzoporphyrin complexes were investigated by the DFT and TDDFT methods. From the above research, some concrete conclusions could be obtained:

The thiophene fragment is very effective for producing strong CT band (*S*_0_ → *S*_1_) that bring a red-shift and broadening of the UV–Vis absorption spectrum of this system. The benzoporphyrin is a saddle-shaped structure, which does not contribute to the CT band but effectively to *π* → π* transition (*S*_0_ → *S*_2_). By using different acceptors (anthraquinone/naphtoquinone/isoindigo), the light-absorption ability, energy levels and the degree of the CT process can be modulated. The theoretical phosphorescence wavelength of this series of derivatives ranges from 1000 to 1200 nm.

## Supplementary Material

Image of the optimized structures, representations of the frontier MOs, computed positions of the electronic transitions, oscillator strength (f), major contributions and the calculated absorption spectra for all molecules are given

## Data accessibility

The datasets supporting this article have been uploaded as electronic supplementary material: image of the optimized structures, representations of the frontier MOs, computed positions of the electronic transitions, oscillator strength (*f*), major contributions and the calculated absorption spectra for all molecules are given.

## References

[1] Woodward RB 1966 *Aromaticity: an international symposium*. Sheffield, UK: The Chemical Society.

[2] Kundu S, Patra A 2017 Nanoscale strategies for light harvesting. Chem. Rev. 117, 712–757. (10.1021/acs.chemrev.6b00036) 27494796

[3] Ishida YJ, Shimada T, Masui D, Tachibana H, Inoue H, Takagi S 2011 Efficient excited energy transfer reaction in clay/porphyrin complex toward an artificial light-harvesting system. J. Am. Chem. Soc. 133, 14 280–14 286. (10.1021/ja204425u) 21809841

[4] Zhang X, Ballem MA, Hu ZJ, Bergman P, Uvdal K 2011 Nanoscale light-harvesting metal–organic frameworks. Angew. Chem. Int. Ed. 123, 5847–5851. (10.1002/ange.201007277) 21557406

[5] Lee H, Jeong YH, Kim JH, Kim I, Lee E, Jang WD 2015 Supramolecular coordination polymer formed from artificial light-harvesting dendrimer. J. Am. Chem. Soc. 137, 12 394–12 399. (10.1021/jacs.5b08092) 26349620

[6] Lovell JF, Liu TWB, Chen J, Zheng G 2010 Activatable photosensitizers for imaging and therapy. Chem. Rev. 110, 2839–2857. (10.1021/cr900236h) 20104890

[7] Zhang W, Lu J, Gao X, Li P, Zhang W, Ma Y, Wang H, Tang B. 2018 Enhanced photodynamic therapy by reduced levels of intracellular glutathione obtained by employing a nano-MOF with Cu^II^ as the active center. Angew. Chem. Int. Ed. 130, 4985–4990. (10.1002/ange.201710800) 29451722

[8] Wang X, Brisard G, Fortin D, Karsenti PL, Harvey PD 2015 Push–pull porphyrin-containing polymers: materials exhibiting ultrafast near-IR photophysics. Macromolecules 48, 7024–7038. (10.1021/acs.macromol.5b01607)

[9] Bucher L, Desbois N, Harvey PD, Sharma GD, Gros CP 2017 Porphyrins and BODIPY as building blocks for efficient donor materials in bulk heterojunction solar cells. Sol. RRL 1, 1700127 (10.1002/solr.201700127)

[10] Keawsongsaeng W et al. 2016 Systematic investigation of porphyrin-thiophene conjugates for ternary bulk heterojunction solar cells. Adv. Energy Mater. 6, 1600957 (10.1002/aenm.201600957)

[11] Frisch MJ et al. 2016 Gaussian, *Gaussian 16*, Revision A.03, (Gaussian, Inc., Wallingford, CT).

[12] Hohenberg P, Kohn W 1964 Inhomogeneous electron gas. Phys. Rev. 136, B864–B871. (10.1103/PhysRev.136.B864)

[13] Kohn W, Sham L 1965 Self-consistent equations including exchange and correlation effects. J. Phys. Rev. 140, A1133–A1138. (10.1103/PhysRev.140.A1133)

[14] Salahub DR, Zerner MC 1989 Challenge of d and f electrons. In *Chemical Congress of North America 1988: Toronto, Canada.* Washington, DC: American Chemical Society.

[15] Parr RG, Yang W 1989 *Density-Functional Theory of Atoms and Molecules*, vol. 16 of International Series of Monographs on Chemistry. Oxford, UK: Oxford University Press.

[16] Stratmann RE, Scuseria GE, Frisch MJ 1998 An efficient implementation of time-dependent density-functional theory for the calculation of excitation energies of large molecules. J. Chem. Phys. 109, 8218–8224. (10.1063/1.477483)

[17] Bauernschmitt R, Ahlrichs R 1996 Treatment of electronic excitations within the adiabatic approximation of time dependent density functional theory. Chem. Phys. Lett. 256, 454–464. (10.1016/0009-2614(96)00440-X)

[18] Casida ME, Jamorski C, Casida KC, Salahub DR 1998 Molecular excitation energies to high-lying bound states from time-dependent density-functional response theory: characterization and correction of the time-dependent local density approximation ionization threshold. J. Chem. Phys. 108, 4439–4449. (10.1063/1.475855)

[19] Becke AD 1993 Density-functional thermochemistry. III. The role of exact exchange. J. Chem. Phys. 98, 5648–5652. (10.1063/1.464913)

[20] Lee C, Yang W, Parr RG 1988 Development of the Colle-Salvetti correlation-energy formula into a functional of the electron density. Phys. Rev. B: Condens. Matter Mater. Phys. 37, 785–789. (10.1103/PhysRevB.37.785) 9944570

[21] Miehlich B, Savin A, Stoll H, Preuss H 1989 Results obtained with the correlation energy density functionals of Becke and Lee, Yang and Parr. Chem. Phys. Lett. 157, 200–206. (10.1016/0009-2614(89)87234-3)

[22] Karthikeyan S, Lee JY 2013 Zinc-porphyrin based dyes for dye-sensitized solar cells. J. Phys. Chem. A 117, 10 973–10 979.(10.1021/jp408473k) 24090130

[23] Zhang MJ, Guo YR, Fang GZ, Pan QJ 2013 DFT/TD-DFT studies on structural and spectroscopic properties of metalloporphyrin complexes: a design of ruthenium porphyrin photosensitizer. Comput. Theor. Chem. 1019, 94–100. (10.1016/j.comptc.2013.07.006)

[24] Ornso KB, Garcia-Lastra JM, Thygesen KS 2013 Computational screening of functionalized zinc porphyrins for dye sensitized solar cells. Phys. Chem. Chem. Phys. 15, 19 478–19 486. (10.1039/C3CP54050B) 24129651

[25] Xie M, Bai F-Q, Wang J, Zheng Y-Q, Lin Z 2018 Theoretical investigations on the unsymmetrical effect of β-link Zn–porphyrin sensitizers on the performance for dye-sensitized solar cells. Phys. Chem. Chem. Phys. 20, 3741–3751. (10.1039/C7CP07115A) 29345699

[26] Lu J, Zhang B, Liu S, Li H, Yuan H, Shen Y, Xu J, Cheng Y, Wang M 2014 A cyclopenta[1,2-*b*:5,4-*b*′]dithiophene–porphyrin conjugate for mesoscopic solar cells: a D–π–D–A approach. Phys. Chem. Chem. Phys. 16, 24 755–24 762. (10.1039/C4CP03425B) 25315179

[27] Jin X, Li D, Sun L, Wang CL, Bai FQ 2018 Theoretical design of porphyrin sensitizers with different acceptors for application in dye-sensitized solar cells. RSC Advances 8, 19 804–19 810. (10.1039/C8RA02974A) PMC9080764 35541014

[28] Binkley JS, Pople JA, Hehre WJ 1980 Self-consistent molecular orbital methods. 21. Small split-valence basis sets for first-row elements. J. Am. Chem. Soc. 102, 939–947. (10.1021/ja00523a008)

[29] Gordon MS, Binkley JS, Pople JA, Pietro WJ, Hehre WJ 1982 Self-consistent molecular-orbital methods. 22. Small split-valence basis sets for second-row elements. J. Am. Chem. Soc. 104, 2797–2803. (10.1021/ja00374a017)

[30] Pietro WJ, Francl MM, Hehre WJ, Defrees DJ, Pople JA, Binkley JS 1982 Self-consistent molecular orbital methods. 24. Supplemented small split-valence basis sets for second-row elements. J. Am. Chem. Soc. 104, 5039–5048. (10.1021/ja00383a007)

[31] Dobbs KD, Hehre WJ 1986 Molecular orbital theory of the properties of inorganic and organometallic compounds 4. Extended basis sets for third-and fourth-row, main-group elements. J. Comput. Chem. 7, 359–378. (10.1002/jcc.540070313)

[32] Dobbs KD, Hehre WJ 1987 Molecular orbital theory of the properties of inorganic and organometallic compounds 5. Extended basis sets for first-row transition metals. J. Comput. Chem. 8, 861–879. (10.1002/jcc.540080614)

[33] Dobbs KD, Hehre WJ 1987 Molecular orbital theory of the properties of inorganic and organometallic compounds. 6. Extended basis sets for second-row transition metals. J. Comput. Chem. 8, 880–893. (10.1002/jcc.540080615)

[34] Godbout N, Salahub DR, Andzelm J, Wimmer E 1992 Optimization of Gaussian-type basis sets for local spin density functional calculations. Part I. Boron through neon, optimization technique and validation. Can. J. Chem. 70, 560–571. (10.1139/v92-079)

[35] Stuttgart RSC. 2008 ECP EMSL Basis Set Exchange Library.

[36] Stevens WJ, Krauss M, Basch H, Jasien PG 1992 Relativistic compact effective potentials and efficient, shared-exponent basis sets for the third-, fourth-, and fifth-row atoms. Can. J. Chem. 70**,** 612–630. (10.1139/v92-085)

[37] O'boyle NM, Tenderholt AL, Langner KM 2008 cclib: a library for package-independent computational chemistry algorithms. J. Comput. Chem. 29, 839–845. (10.1002/jcc.20823) 17849392

[38] Namazian M 2003 Density functional theory response to the calculation of electrode potentials of quinones in non-aqueous solution of acetonitrile. J. Mol. Struct.: THEOCHEM 664–665, 273–278. (10.1016/j.theochem.2003.10.001)

[39] Lowry MS, Hudson WR, Pascal RA Jr, Bernhard S 2004 Accelerated luminophore discovery through combinatorial synthesis. J. Am. Chem. Soc. 126, 14 129–14 135. (10.1021/ja047156+) 15506778

[40] Wang X, Fortin D, Brisard G, Harvey PD 2014 Electronic communication across N-linked unconjugated polymers: important insight into the charge transfer processes of polyaniline. Chem. Commun. 50, 350–352. (10.1039/C3CC47092J) 24247556

[41] Sun Y, Zhang Y, Zhao S, Jiang J, Xie M, Wang X, Zhou S 2017 Research progress of organic solar cells based on porphyrin units. Journal of Liaoning Shihua University 37, 6–12.

