# Supplementary material for: Theoretical investigation of the structural and spectroscopic properties of expanded metalloporphyrin complexes
Source: R Soc Open Sci. 2019 Jan 23;6(1):181199. doi: 10.1098/rsos.181199 (PMC6366163; doi:10.1098/rsos.181199)
Supplement: Image of the optimized structures, representations of the frontier MOs, computed positions of the electronic transitions, oscillator strength (f), major contributions and the calculated absorption spectra for all molecules are given [file rsos181199supp1.docx]

Supporting Information

**Table of Content**

[Figure S1. Structure of AQ-[Zn]-AQ. 1](#_Toc519512370)

[Figure S2. Image of the optimized structure ofAQ-[Zn]-AQ. 1](#_Toc519512371)

[Figure S3. Representations of the frontier MOs of AQ-[Zn]-AQ.The MO energies are inHartree. 2](#_Toc519512372)

[Table S1. Computed positions of the electronic transitions, oscillator strength (f), and major contributions of the AQ-[Zn]-AQ. 3](#_Toc519512373)

[Figure S4. Bar graph (blue) reporting the position of the electronic transitions vs the oscillator strength (f) for AQ-[Zn]-AQ.The black line represents a spectrum where 1000 cm-1 is applied to each transition. 5](#_Toc519512374)

[Table S2. Atomic contributions for the frontier MO for AQ-[Zn]-AQ. 5](#_Toc519512375)

[Figure S5. Structure of AQ-[S]-[Zn]-[S]-AQ. 6](#_Toc519512376)

[Figure S6. Image of the optimized structure ofAQ-[S]-[Zn]-[S]-AQ. 6](#_Toc519512377)

[Figure S7. Representations of the frontier MOs of AQ-[S]-[Zn]-[S]-AQ.The MOenergies are in Hartree. 7](#_Toc519512378)

[Table S3. Computed positions of the electronic transitions, oscillator strength (f), andmajor contributions of the AQ-[S]-[Zn]-[S]-AQ. 8](#_Toc519512379)

[Figure S8. Bar graph (blue) reporting the position of the electronic transitions vs the oscillator strength (f) forAQ-[S]-[Zn]-[S]-AQ.The black line represents a spectrum where 1000 cm-1 is applied to each transition. 10](#_Toc519512380)

[Table S4. Atomic contributions for the frontier MO forAQ-[S]-[Zn]-[S]-AQ. 11](#_Toc519512381)

[Figure S9. Structure of AQ-[B-Zn]-AQ. 11](#_Toc519512382)

[Figure S10. Image of the optimized structure ofAQ-[B-Zn]-AQ. 11](#_Toc519512383)

[Figure S11. Representations of the frontier MOs of AQ-[B-Zn]-AQ.The MO energies are inHartree. 12](#_Toc519512384)

[Table S5. Computed positions of the electronic transitions, oscillator strength (f), and major contributions of the AQ-[B-Zn]-AQ. 13](#_Toc519512385)

[Figure S12. Bar graph (blue) reporting the position of the electronic transitions vs the oscillator strength (f) for AQ-[B-Zn]-AQ.The black line represents a spectrum where 1000 cm-1 is applied to each transition. 15](#_Toc519512386)

[Table S6. Atomic contributions for the frontier MO for AQ-[B-Zn]-AQ. 15](#_Toc519512387)

[Figure S13. Structure of AQ-[S]-[B-Zn]-[S]-AQ. 16](#_Toc519512388)

[Figure S14. Image of the optimized structure ofAQ-[S]-[B-Zn]-[S]-AQ. 16](#_Toc519512389)

[Figure S15. Representations of the frontier MOs of AQ-[S]-[B-Zn]-[S]-AQ.The MO energies arein Hartree. 17](#_Toc519512390)

[Table S7. Computed positions of the electronic transitions, oscillator strength (f), and major contributions of the AQ-[S]-[B-Zn]-[S]-AQ. 18](#_Toc519512391)

[Figure S16. Bar graph (blue) reporting the position of the electronic transitions vs the oscillator strength (f) for AQ-[S]-[B-Zn]-[S]-AQ.The black line represents a spectrum where 1000 cm-1 is applied to each transition. 20](#_Toc519512392)

[Table S8. Atomic contributions for the frontier MO for AQ-[S]-[B-Zn]-[S]-AQ. 20](#_Toc519512393)

[Figure S17. Structure of NQ-[Zn]-NQ. 21](#_Toc519512394)

[Figure S18. Image of the optimized structure ofNQ-[Zn]-NQ. 21](#_Toc519512395)

[Figure S19. Representations of the frontier MOs ofNQ-[Zn]-NQ.The MO energies are in Hartree. 22](#_Toc519512396)

[Table S9. Computed positions of the electronic transitions, oscillator strength (f), andmajor contributions of the NQ-[Zn]-NQ. 23](#_Toc519512397)

[Figure S20. Bar graph (blue) reporting the position of the electronic transitions vs the oscillator strength (f) for NQ-[Zn]-NQ.The black line represents a spectrum where 1000 cm-1 is applied to each transition. 25](#_Toc519512398)

[Table S10. Atomic contributions for the frontier MO for NQ-[Zn]-NQ. 25](#_Toc519512399)

[Figure S21. Structure of NQ-[S]-[Zn]-[S]-NQ. 26](#_Toc519512400)

[Figure S22. Image of the optimized structure ofNQ-[S]-[Zn]-[S]-NQ. 26](#_Toc519512401)

[Figure S23. Representations of the frontier MOs ofNQ-[S]-[Zn]-[S]-NQ.The MO energies are in Hartree. 27](#_Toc519512402)

[Table S11. Computed positions of the electronic transitions, oscillator strength (f), and major contributions of the NQ-[S]-[Zn]-[S]-NQ. 28](#_Toc519512403)

[Figure S24. Bar graph (blue) reporting the position of the electronic transitions vs the oscillator strength (f) for NQ-[S]-[Zn]-[S]-NQ.The black line represents a spectrum where 1000 cm-1 is applied to each transition. 31](#_Toc519512404)

[Table S12. Atomic contributions for the frontier MO for NQ-[S]-[Zn]-[S]-NQ. 31](#_Toc519512405)

[Figure S26. Image of the optimized structure of NQ-[B-Zn]-NQ. 32](#_Toc519512406)

[Figure S27. Representations of the frontier MOs of NQ-[B-Zn]-NQ.The MO energies are inHartree. 33](#_Toc519512407)

[Table S13. Computed positions of the electronic transitions, oscillator strength (f), and major contributions of the NQ-[B-Zn]-NQ. 34](#_Toc519512408)

[Figure S28. Bar graph (blue) reporting the position of the electronic transitions vs the oscillator strength (f) for NQ-[B-Zn]-NQ.The black line represents a spectrum where 1000 cm-1 is applied to each transition. 36](#_Toc519512409)

[Table S14. Atomic contributions for the frontier MO for NQ-[B-Zn]-NQ. 36](#_Toc519512410)

[Figure S29. Structure of NQ-[S]-[B-Zn]-[S]-NQ. 37](#_Toc519512411)

[Figure S30. Image of the optimized structure of NQ-[S]-[B-Zn]-[S]-NQ. 37](#_Toc519512412)

[Figure S31. Representations of the frontier MOs of NQ-[S]-[B-Zn]-[S]-NQ.The MO energies are in Hartree. 38](#_Toc519512413)

[Table S15. Computed positions of the electronic transitions,oscillator strength (f), and major contributions of the NQ-[S]-[B-Zn]-[S]-NQ. 39](#_Toc519512414)

[Figure S32. Bar graph (blue) reporting the position of the electronic transitions vs the oscillator strength (f) for NQ-[S]-[B-Zn]-[S]-NQ.The black line represents a spectrum where 1000 cm-1 is applied to each transition. 41](#_Toc519512415)

[Table S16. Atomic contributions for the frontier MO for NQ-[S]-[B-Zn]-[S]-NQ. 42](#_Toc519512416)

[Figure S33. Structure of ID-[Zn]-ID. 42](#_Toc519512417)

[Figure S34. Image of the optimized structure of ID-[Zn]-ID. 42](#_Toc519512418)

[Figure S35. Representations of the frontier MOs ofID-[Zn]-ID.The MO energies are in Hartree. 43](#_Toc519512419)

[Table S17. Computed positions of the electronic transitions, oscillator strength (f), andmajor contributions of the ID-[Zn]-ID. 44](#_Toc519512420)

[Figure S36. Bar graph (blue) reporting the position of the electronic transitions vs the oscillator strength (f) for ID-[Zn]-ID.The black line represents a spectrum where 1000 cm-1 is applied to each transition. 46](#_Toc519512421)

[Table S18. Atomic contributions for the frontier MO for ID-[Zn]-ID. 47](#_Toc519512422)

[Figure S37. Structure of NQ-[S]-[Zn]-[S]-NQ. 47](#_Toc519512423)

[Figure S38. Image of the optimized structure of NQ-[S]-[Zn]-[S]-NQ. 47](#_Toc519512424)

[Figure S39. Representations of the frontier MOs of NQ-[S]-[Zn]-[S]-NQ.The MO energies are inHartree. 48](#_Toc519512425)

[Table S19. Computed positions of the electronic transitions, oscillator strength (f), and major contributions of the NQ-[S]-[Zn]-[S]-NQ. 49](#_Toc519512426)

[Figure S40. Bar graph (blue) reporting the position of the electronic transitions vs the oscillator strength (f) for NQ-[S]-[Zn]-[S]-NQ.The black line represents a spectrum where 1000 cm-1 is applied to each transition. 51](#_Toc519512427)

[Table S20. Atomic contributions for the frontier MO for NQ-[S]-[Zn]-[S]-NQ. 52](#_Toc519512428)

[Figure S41. Structure of ID-[B-Zn]-ID. 52](#_Toc519512429)

[Figure S42. Image of the optimized structure of ID-[B-Zn]-ID. 52](#_Toc519512430)

[Figure S43. Representations of the frontier MOs of ID-[B-Zn]-ID.The MO energies are in Hartree. 53](#_Toc519512431)

[Table S21. Computed positions of the electronic transitions, oscillator strength (f), and major contributions of the ID-[B-Zn]-ID. 54](#_Toc519512432)

[Figure S44. Bar graph (blue) reporting the position of the electronic transitions vs the oscillator strength (f) for ID-[B-Zn]-ID.The black line represents a spectrum where 1000 cm-1 is applied to each transition. 56](#_Toc519512433)

[Table S22. Atomic contributions for the frontier MO for ID-[B-Zn]-ID. 57](#_Toc519512434)

[Figure S45. Structure of ID-[S]-[B-Zn]-[S]-ID. 57](#_Toc519512435)

[Figure S46. Image of the optimized structure of ID-[S]-[B-Zn]-[S]-ID. 57](#_Toc519512436)

[Figure S47. Representations of the frontier MOs ofID-[S]-[B-Zn]-[S]-ID.The MO energies are in Hartree. 58](#_Toc519512437)

[Table S23. Computed positions of the electronic transitions, oscillator strength (f), and major contributions of the ID-[S]-[B-Zn]-[S]-ID. 59](#_Toc519512438)

[Figure S48. Bar graph (blue) reporting the position of the electronic transitions vs the oscillator strength (f) for ID-[S]-[B-Zn]-[S]-ID.The black line represents a spectrum where 1000 cm-1 is applied to each transition. 61](#_Toc519512439)

[Table S24. Atomic contributions for the frontier MO for ID-[S]-[B-Zn]-[S]-ID. 62](#_Toc519512440)

**AQ**-[**Zn**]-**AQ**

# Figure S1. Structure of AQ-[Zn]-AQ.


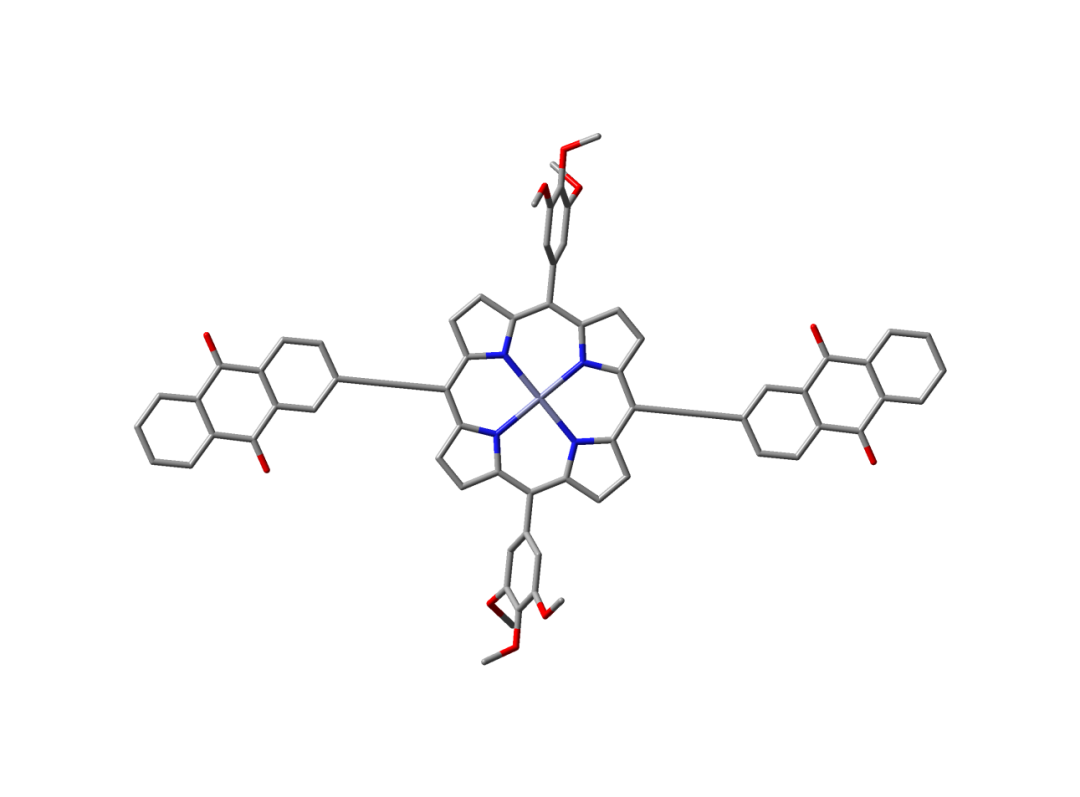


# Figure S2. Image of the optimized structure ofAQ-[Zn]-AQ.


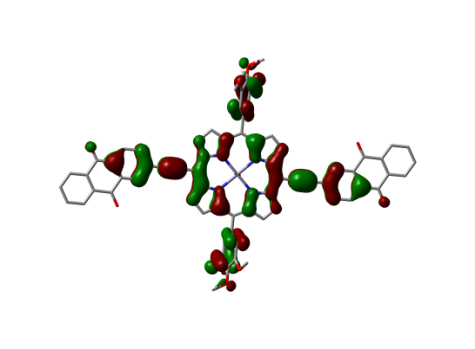


H-4 (-0.23267)


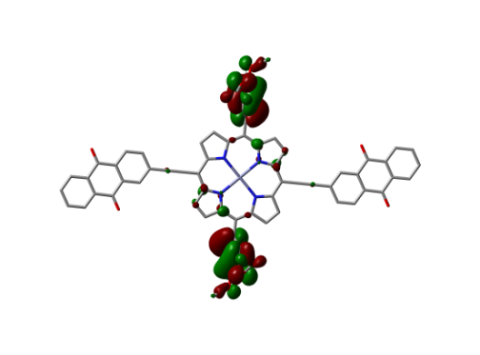


H-3 (-0.22432)


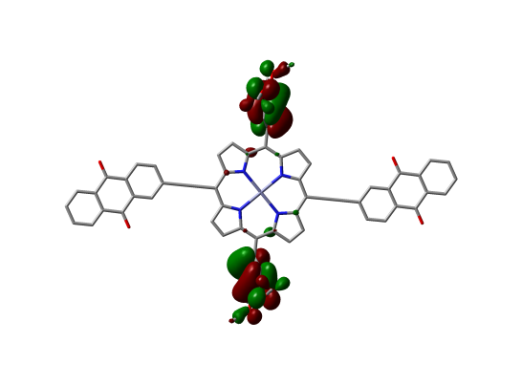


H-2 (-0.22301)


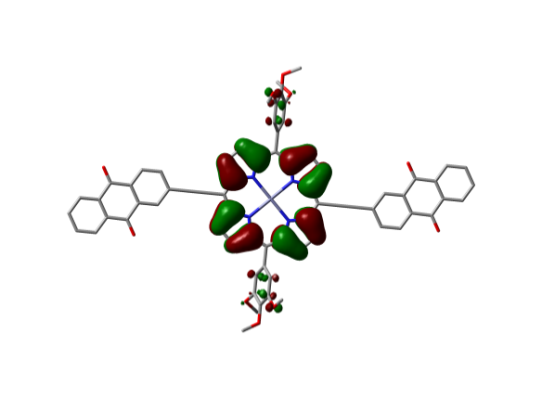


H-1 (-0.20337)


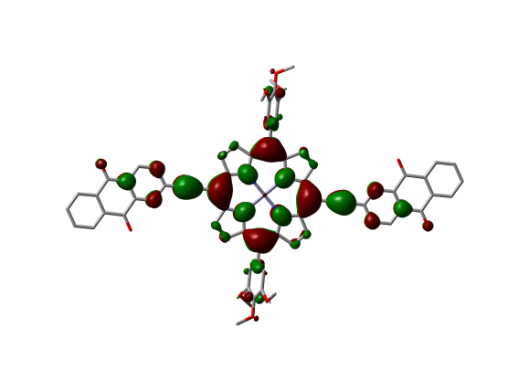


HOMO (-0.19115)


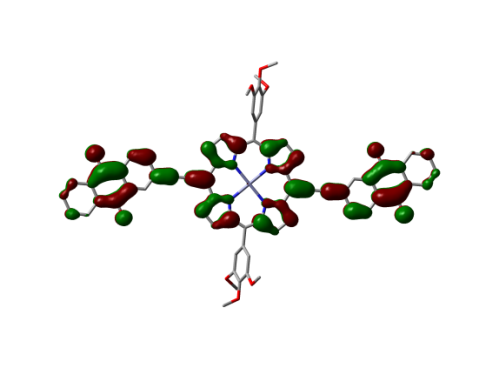


LUMO (-0.11610)


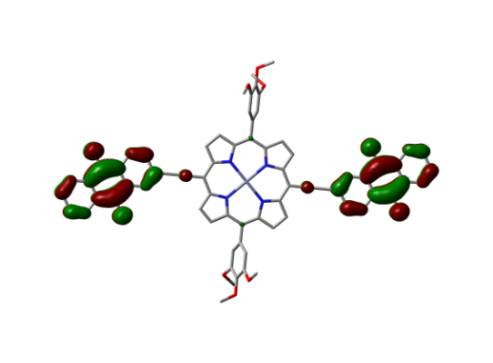


L+1 (-0.10836)


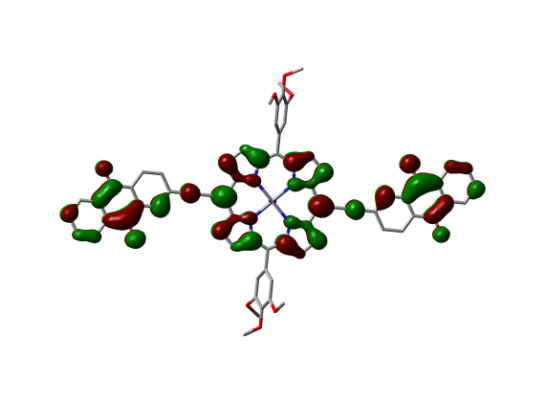


L+2 (-0.09850)


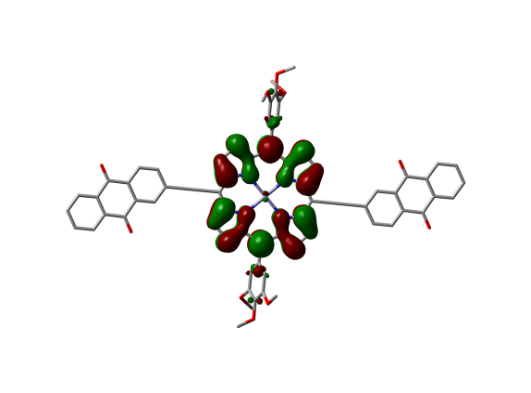


L+3 (-0.09040)


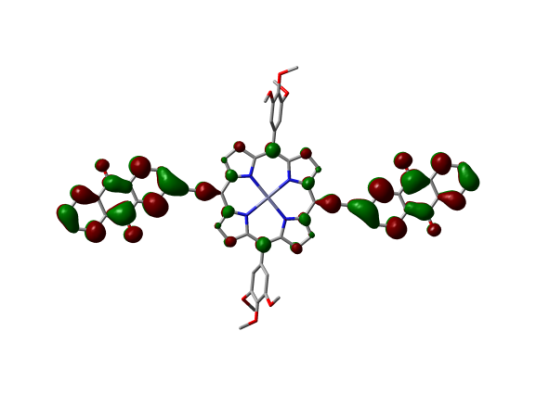


L+4 (-0.06644)

# Figure S3.Representations of the frontier MOs of AQ-[Zn]-AQ.The MO energies are inHartree.

# Table S1.Computed positions of the electronic transitions, oscillator strength (f), and major contributions of the AQ-[Zn]-AQ.

| Wavelength (nm) | Oscillator strength (f) | Major contributions (%) |
| --- | --- | --- |
| 701.3 | 1.2960 | HOMO→LUMO (96) |
| 622.9 | 0.0000 | HOMO→L+1 (99) |
| 612.6 | 0.0161 | H-1→LUMO (71), HOMO→L+3 (27) |
| 563.6 | 0.0257 | H-1→L+3 (12), HOMO→L+2 (87) |
| 538.5 | 0.0000 | H-1→L+1 (100) |
| 521.1 | 0.0812 | H-1→LUMO (22), H-1→L+2 (46), HOMO→L+3 (32) |
| 479.0 | 0.0002 | H-2→LUMO (97) |
| 473.9 | 0.0851 | H-3→LUMO (93) |
| 439.1 | 0.0001 | H-4→LUMO (91) |
| 437.4 | 1.7666 | H-1→L+3 (48) |
| 437.0 | 1.2332 | H-1→L+2 (34), H-1→L+3 (18), HOMO→L+3 (27) |
| 430.0 | 0.0170 | H-2→L+1 (96) |
| 425.9 | 0.0000 | H-5→LUMO (88) |
| 425.2 | 0.0000 | H-3→L+1 (91) |
| 422.8 | 0.0714 | H-6→LUMO (84) |
| 419.5 | 0.0001 | H-13→L+1 (39), H-12→LUMO (36), H-12→L+2 (10) |
| 419.5 | 0.0000 | H-13→LUMO (36), H-13→L+2 (11), H-12→L+1 (39) |
| 409.2 | 0.0044 | H-7→LUMO (80), H-7→L+2 (16) |
| 405.2 | 0.0001 | HOMO→L+4 (91) |
| 403.8 | 0.0116 | H-4→L+1 (70) |
| 402.1 | 0.0002 | H-2→L+2 (95) |
| 397.0 | 0.0520 | H-3→L+2 (84) |
| 396.9 | 0.0002 | H-2→L+3 (91) |
| 392.0 | 0.1201 | H-3→L+3 (78) |
| 387.4 | 0.0000 | H-9→LUMO (68), H-6→L+1 (17) |
| 386.7 | 0.0006 | H-5→L+1 (79) |
| 386.3 | 0.0227 | H-8→LUMO (80) |
| 385.7 | 0.0000 | H-9→LUMO (15), H-6→L+1 (66), H-4→L+2 (11) |
| 383.8 | 0.0000 | H-22→L+1 (36), H-21→LUMO (31), H-21→L+2 (11) |
| 383.8 | 0.0000 | H-22→LUMO (31), H-22→L+2 (11), H-21→L+1 (36) |
| 378.4 | 0.0000 | H-10→LUMO (19), H-6→L+1 (14), H-4→L+2 (52) |
| 378.3 | 0.0144 | HOMO→L+5 (71) |
| 369.6 | 0.0001 | H-10→LUMO (61), H-4→L+2 (27) |
| 366.5 | 0.0000 | H-4→L+3 (45), H-1→L+4 (46) |
| 364.1 | 0.0000 | H-5→L+2 (62), H-1→L+4 (20) |
| 362.6 | 0.0001 | H-5→L+2 (19), H-5→L+3 (17), H-4→L+3 (37), H-1→L+4 (21) |
| 362.4 | 0.0129 | H-6→L+2 (94) |
| 359.3 | 0.0643 | H-6→L+3 (82) |
| 359.0 | 0.0002 | H-5→L+3 (76), H-1→L+4 (11) |
| 356.3 | 0.0014 | H-7→L+3 (94) |
| 351.8 | 0.0000 | H-14→LUMO (34), H-11→LUMO (29) |
| 349.3 | 0.0000 | H-7→L+1 (99) |
| 345.9 | 0.1954 | H-14→LUMO (42), H-11→LUMO (28) |
| 345.8 | 0.0000 | HOMO→L+6 (79) |
| 344.9 | 0.0837 | H-1→L+5 (94) |
| 344.9 | 0.0000 | H-8→L+1 (93) |
| 342.9 | 0.0038 | H-9→L+1 (89) |
| 338.5 | 0.0000 | H-20→L+1 (13), H-19→LUMO (24), H-11→L+1 (34) |
| 337.0 | 0.0015 | H-7→LUMO (17), H-7→L+2 (81) |
| 334.3 | 0.0205 | H-10→L+1 (70) |
| 334.0 | 0.1204 | H-20→LUMO (16), H-16→LUMO (42), H-15→L+1 (22) |
| 333.8 | 0.0000 | H-16→L+1 (26), H-15→LUMO (53) |
| 331.3 | 0.0037 | H-20→LUMO (24), H-19→L+1 (11), H-16→LUMO (17), H-10→L+1 (12) |
| 328.6 | 0.0043 | H-8→L+2 (83) |
| 328.0 | 0.0000 | H-19→LUMO (12), H-11→L+1 (21), H-9→L+3 (13) |
| 327.9 | 0.0157 | H-8→L+3 (90) |
| 327.5 | 0.0000 | H-9→L+2 (71) |
| 326.1 | 0.0000 | H-25→LUMO (17), H-13→L+1 (13), H-12→LUMO (43), H-12→L+2 (14) |
| 326.0 | 0.0000 | H-27→LUMO (16), H-13→LUMO (44), H-13→L+2 (14), H-12→L+1 (14) |
| 325.2 | 0.0000 | H-11→L+1 (19), H-9→L+3 (62) |
| 319.1 | 0.0000 | H-10→L+2 (11), H-10→L+3 (47), H-1→L+6 (28) |
| 319.0 | 0.0003 | H-18→LUMO (83) |
| 318.9 | 0.0000 | H-17→LUMO (86) |
| 318.4 | 0.0000 | H-14→L+1 (16), H-10→L+2 (44), H-10→L+3 (10), H-9→L+3 (13) |
| 315.4 | 0.0000 | H-27→LUMO (58), H-13→LUMO (12), H-12→L+1 (11) |
| 315.3 | 0.0001 | H-25→LUMO (56), H-13→L+1 (10), H-12→LUMO (12) |
| 313.9 | 0.0155 | H-24→LUMO (15), H-11→L+2 (44), HOMO→L+7 (21) |
| 312.4 | 0.0001 | H-10→L+3 (34), H-1→L+6 (54) |
| 311.0 | 0.0099 | H-2→L+4 (96) |
| 311.0 | 0.0000 | H-14→L+1 (78), H-10→L+2 (16) |
| 308.7 | 0.0001 | H-3→L+4 (91) |
| 306.6 | 0.0000 | H-23→LUMO (53), H-19→LUMO (12) |
| 304.9 | 0.0001 | H-16→LUMO (33), H-16→L+2 (11), H-15→L+1 (47) |
| 304.8 | 0.0000 | H-16→L+1 (47), H-15→LUMO (32), H-15→L+2 (10) |
| 303.6 | 0.0570 | H-24→LUMO (21), H-20→LUMO (12), H-11→L+2 (39), HOMO→L+7 (14) |
| 301.2 | 0.1327 | H-24→LUMO (25), H-14→L+2 (17), HOMO→L+7 (43) |
| 299.7 | 0.0019 | H-21→LUMO (34), H-13→L+1 (15), H-12→L+2 (18) |
| 299.7 | 0.0000 | H-22→LUMO (36), H-22→L+2 (10), H-13→L+2 (18), H-12→L+1 (16) |
| 299.6 | 0.0316 | H-20→LUMO (28), H-20→L+2 (10), H-19→L+1 (40) |
| 299.3 | 0.0461 | H-14→L+2 (60), H-14→L+3 (10), HOMO→L+7 (10) |
| 299.3 | 0.0000 | H-20→L+1 (41), H-19→LUMO (26), H-19→L+2 (10) |
| 298.8 | 0.0168 | H-14→L+2 (10), H-14→L+3 (79) |
| 298.4 | 0.0003 | H-17→L+1 (94) |
| 298.3 | 0.0000 | H-18→L+1 (92) |
| 296.8 | 0.0001 | H-2→L+5 (94) |
| 296.7 | 0.0000 | HOMO→L+8 (81) |
| 295.8 | 0.0003 | H-22→L+1 (14), H-21→LUMO (20), H-13→L+1 (13), H-12→L+2 (45) |
| 295.8 | 0.0000 | H-22→LUMO (20), H-21→L+1 (14), H-13→L+2 (45), H-12→L+1 (13) |
| 295.6 | 0.1634 | H-11→L+3 (14), H-4→L+4 (42), H-3→L+5 (31) |
| 294.1 | 0.0003 | H-26→LUMO (80) |
| 294.1 | 0.4587 | H-11→L+3 (64), H-4→L+4 (19) |
| 293.8 | 0.0507 | H-4→L+4 (22), H-3→L+5 (67) |
| 288.0 | 0.0424 | H-27→L+1 (16), H-25→L+2 (11), H-23→L+1 (38) |
| 287.8 | 0.0000 | H-27→L+2 (32), H-25→L+1 (52) |
| 287.7 | 0.0199 | H-27→L+1 (36), H-25→L+2 (24), H-23→L+1 (14) |
| 287.5 | 0.0174 | H-23→L+1 (11), H-5→L+4 (76) |
| 287.3 | 0.0002 | H-6→L+4 (75) |
| 285.9 | 0.0000 | H-24→L+1 (56), H-23→L+2 (10) |
| 284.0 | 0.0000 | H-16→L+1 (14), H-15→L+2 (67) |
| 283.8 | 0.0000 | H-28→LUMO (25), H-17→L+2 (66) |

**
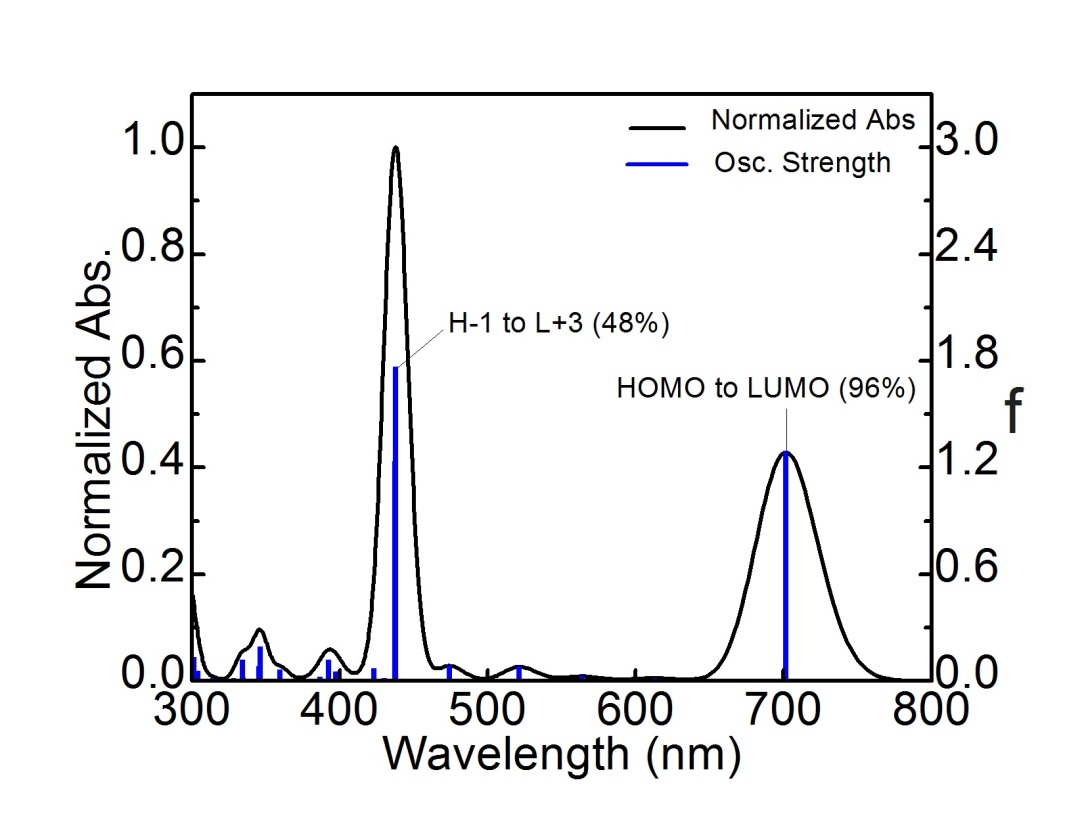
**

# Figure S4.Bar graph (blue) reporting the position of the electronic transitions vs the oscillator strength (f) for AQ-[Zn]-AQ.The black line represents a spectrum where 1000 cm-1 is applied to each transition.

# Table S2.Atomic contributions for the frontier MO for AQ-[Zn]-AQ.

|  | H-4 | H-3 | H-2 | H-1 | H | L | L+1 | L+2 | L+3 | L+4 |
| --- | --- | --- | --- | --- | --- | --- | --- | --- | --- | --- |
| Ethyne-porphyrins | 0.76 | 0.99 | 0.99 | ~1.00 | 0.88 | 0.40 | 0.05 | 0.48 | ~1.00 | 0.19 |
| 2*anthranquinone | 0.24 | 0.01 | 0.01 | ~0.0 | 0.12 | 0.60 | 0.95 | 0.52 | ~0.0 | 0.81 |

**AQ**-[**S**]-[**Zn**]-[**S**]-**AQ**

# Figure S5.Structure of AQ-[S]-[Zn]-[S]-AQ.

**
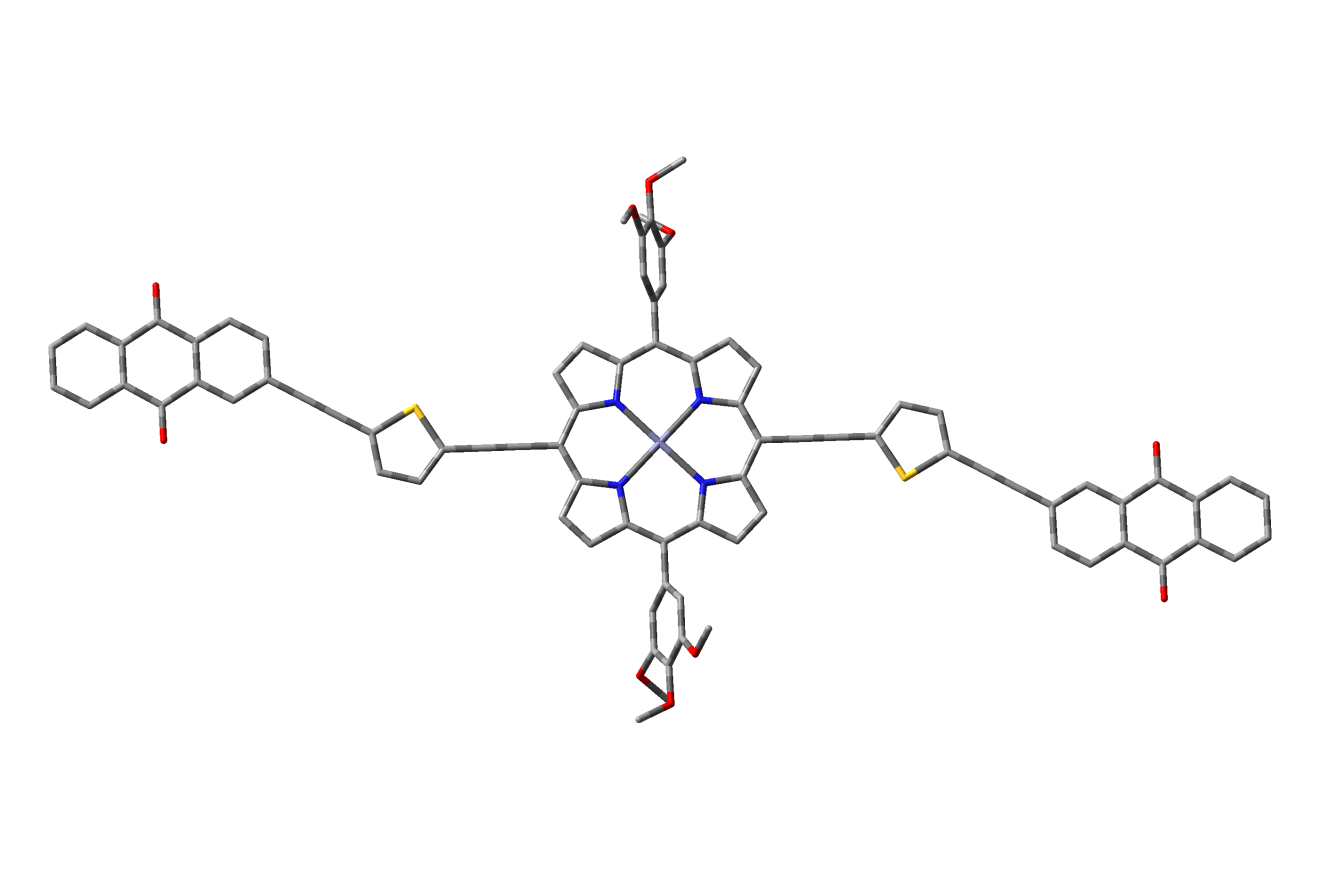
**

# Figure S6. Image of the optimized structure ofAQ-[S]-[Zn]-[S]-AQ.


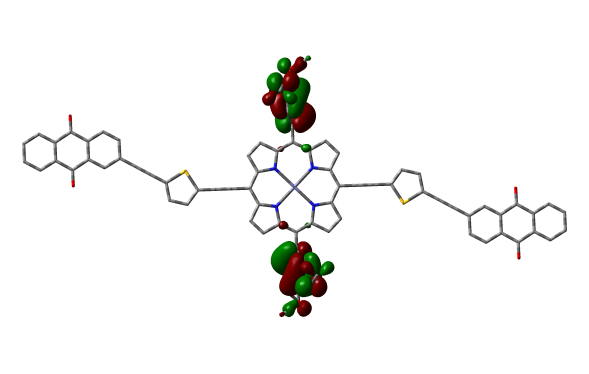


H-4 (-0.21643)


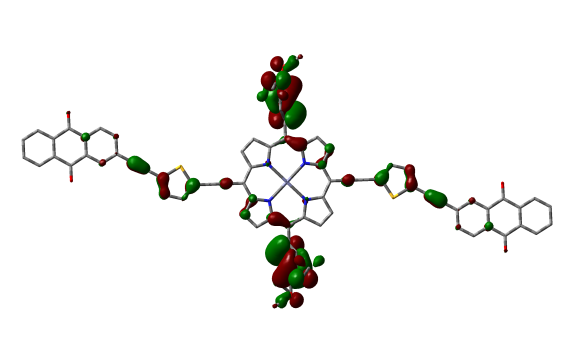


H-3 (-0.21593)


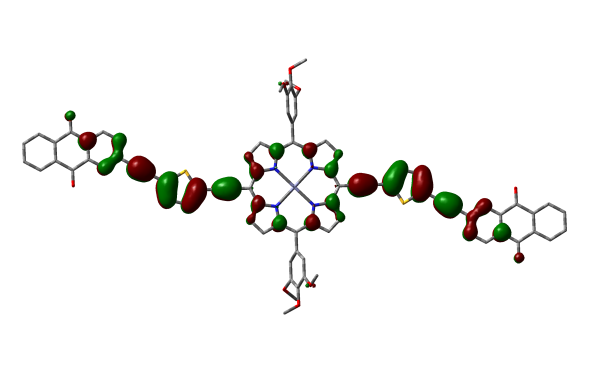


H-2 (-0.20639)


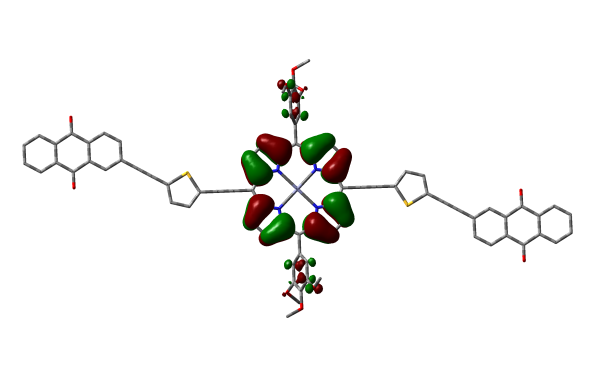


H-1 (-0.19873)


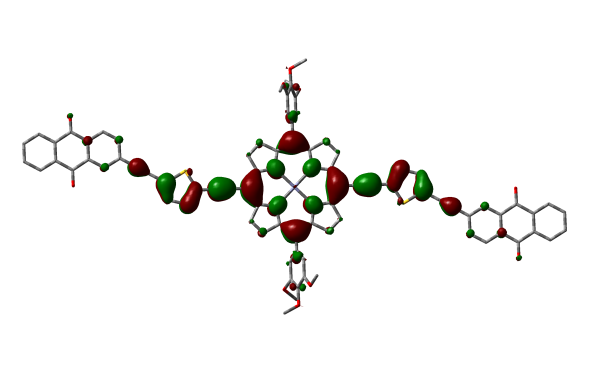


HOMO (-0.17973)


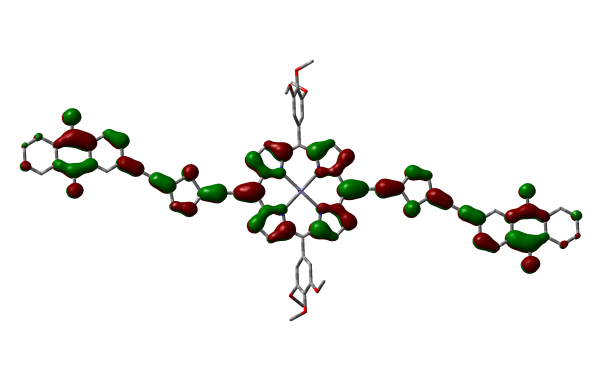


LUMO (-0.10904)


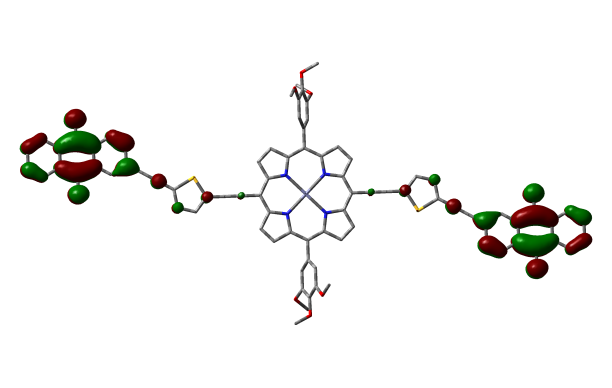


L+1 (-0.10369)


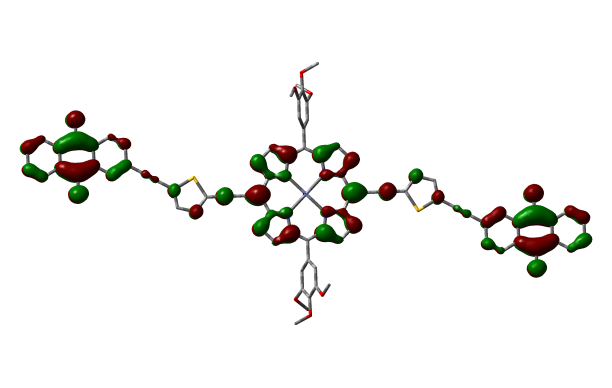


L+2 (-0.09872)


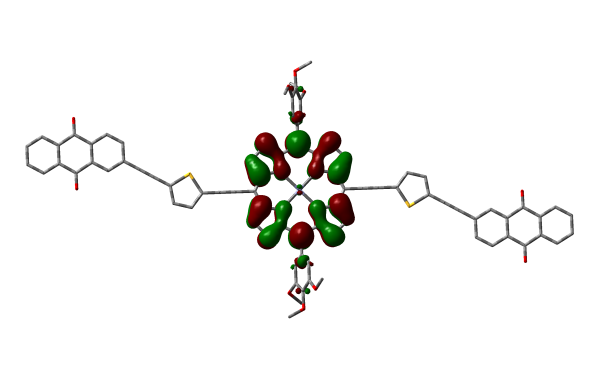


L+3 (-0.08540)

.
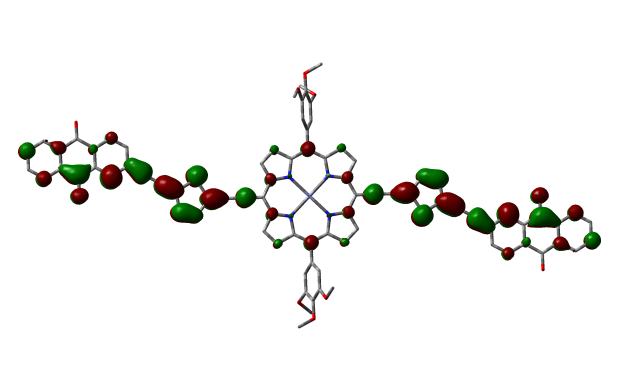


L+4 (-0.07308)

#

# Figure S7.Representations of the frontier MOs of AQ-[S]-[Zn]-[S]-AQ.The MOenergies are in Hartree.

#

# Table S3. Computed positions of the electronic transitions, oscillator strength (f), andmajor contributions of the AQ-[S]-[Zn]-[S]-AQ.

| Wavelength (nm) | Osc. Strength | Major contribs |
| --- | --- | --- |
| 748.7 | 2.4636 | HOMO→LUMO (95%) |
| 661.8 | 0 | HOMO→L+1 (99%) |
| 622.8 | 0.0887 | HOMO→L+2 (94%) |
| 615.5 | 0.0007 | H-1→LUMO (43%), HOMO→L+3 (48%) |
| 524.9 | 0 | H-2→LUMO (95%) |
| 518.9 | 0.1474 | H-1→LUMO (50%), H-1→L+2 (-28%), HOMO→L+3 (-20%) |
| 517.2 | 0 | H-1→L+1 (99%) |
| 490.5 | 1.1445 | H-3→LUMO (-28%), H-2→L+1 (51%), H-1→L+3 (-12%) |
| 478.8 | 0.1642 | H-3→LUMO (38%), H-2→L+1 (34%), H-1→L+2 (-13%) |
| 476.2 | 0.0003 | H-4→LUMO (86%) |
| 473.0 | 0 | H-2→L+2 (13%), HOMO→L+4 (79%) |
| 467.9 | 0.9985 | H-5→LUMO (12%), H-1→L+2 (30%), H-1→L+3 (20%), HOMO→L+3 (-15%) |
| 462.4 | 0 | H-2→L+2 (79%), HOMO→L+4 (-14%) |
| 461.0 | 0.6798 | H-3→LUMO (-21%), H-1→L+2 (-16%), H-1→L+3 (23%), HOMO→L+3 (11%), HOMO→L+5 (10%) |
| 443.1 | 0.124 | H-5→LUMO (-43%), HOMO→L+5 (47%) |
| 431.0 | 0 | H-3→L+1 (94%) |
| 428.1 | 0.0001 | H-4→L+1 (98%) |
| 426.4 | 0 | H-6→LUMO (35%), H-2→L+3 (57%) |
| 421.8 | 0 | H-6→LUMO (54%), H-2→L+3 (-38%) |
| 420.9 | 0 | H-14→L+1 (30%), H-13→LUMO (20%), H-13→L+1 (16%), H-13→L+2 (-17%) |
| 420.9 | 0 | H-14→LUMO (20%), H-14→L+1 (-16%), H-14→L+2 (-17%), H-13→L+1 (30%) |
| 420.5 | 0.0049 | H-7→LUMO (12%), H-3→L+2 (49%), HOMO→L+5 (-14%) |
| 417.8 | 0.0029 | H-7→LUMO (42%), H-3→L+2 (-34%) |
| 416.6 | 0.0001 | H-4→L+2 (87%) |
| 411.1 | 0.0154 | H-8→LUMO (56%), H-8→L+2 (18%) |
| 407.8 | 0.0001 | H-5→L+1 (81%) |
| 406.2 | 0.0634 | H-8→LUMO (16%), H-7→LUMO (17%), H-3→L+3 (40%) |
| 403.0 | 0.0002 | H-4→L+3 (82%) |
| 399.6 | 0.0432 | H-5→L+2 (40%), H-3→L+3 (34%) |
| 396.5 | 0 | H-1→L+4 (91%) |
| 387.9 | 0.1611 | H-6→L+1 (-12%), H-5→L+2 (34%) |
| 385.2 | 0 | H-25→LUMO (23%), H-25→L+1 (43%), H-25→L+2 (-21%) |
| 385.2 | 0 | H-26→LUMO (-23%), H-26→L+1 (43%), H-26→L+2 (21%) |
| 384.2 | 0.0353 | H-6→L+1 (85%) |
| 382.3 | 0 | HOMO→L+6 (84%) |
| 381.5 | 0.0041 | H-10→LUMO (74%), H-10→L+2 (16%) |
| 380.7 | 0 | H-11→LUMO (23%), H-9→LUMO (45%) |
| 380.1 | 0 | H-11→LUMO (53%), H-11→L+2 (11%), H-9→LUMO (-24%) |
| 376.9 | 0 | H-7→L+1 (71%), H-6→L+2 (10%) |
| 374.8 | 0 | H-6→L+2 (79%) |
| 372.6 | 0.1681 | H-5→L+3 (45%), H-2→L+4 (15%), H-1→L+5 (19%) |
| 369.2 | 0.2148 | H-2→L+4 (53%), HOMO→L+7 (-24%) |
| 368.4 | 0.0368 | H-7→L+2 (74%), HOMO→L+7 (12%) |
| 366.5 | 0.2151 | H-7→L+3 (18%), H-1→L+5 (59%), HOMO→L+7 (-10%) |
| 365.0 | 0.0003 | H-6→L+3 (94%) |
| 361.5 | 0.0052 | H-8→L+3 (92%) |
| 355.9 | 0.2504 | H-2→L+4 (14%), HOMO→L+7 (37%) |
| 350.4 | 0 | H-3→L+4 (-11%), H-2→L+5 (66%) |
| 346.3 | 0 | H-20→L+1 (-12%), H-12→LUMO (30%), H-9→L+2 (-12%), H-8→L+1 (11%) |
| 346.3 | 0.0077 | H-9→L+1 (69%) |
| 344.5 | 0.2127 | H-7→L+3 (60%), H-5→L+3 (17%) |
| 344.2 | 0 | H-8→L+1 (87%) |
| 342.0 | 0.0455 | H-15→LUMO (50%), H-15→L+2 (10%) |
| 340.3 | 0 | H-3→L+4 (43%), H-2→L+5 (14%), HOMO→L+8 (31%) |
| 339.1 | 0.0072 | H-8→LUMO (-22%), H-8→L+2 (68%) |
| 339.0 | 0.0043 | H-4→L+4 (94%) |
| 338.8 | 0 | H-9→L+2 (-10%), H-3→L+4 (40%), HOMO→L+8 (-39%) |
| 336.6 | 0 | H-10→L+1 (93%) |
| 335.2 | 0.0058 | H-11→L+1 (84%), H-10→L+2 (10%) |
| 334.7 | 0.1054 | H-24→LUMO (-10%), H-21→L+1 (22%), H-20→LUMO (13%), H-9→L+1 (15%) |
| 332.8 | 0 | H-24→L+1 (-11%), H-21→LUMO (16%), H-18→L+1 (13%), H-9→L+2 (-12%) |
| 331.6 | 0.0783 | H-19→L+1 (38%), H-18→LUMO (-37%), H-18→L+2 (12%) |
| 331.6 | 0 | H-19→LUMO (-16%), H-18→L+1 (14%), H-12→LUMO (15%), H-9→L+2 (33%) |
| 331.0 | 0 | H-11→LUMO (-11%), H-11→L+2 (24%), H-9→L+3 (44%) |
| 329.9 | 0 | H-19→LUMO (13%), H-12→LUMO (22%) |
| 329.6 | 0.0085 | H-11→L+1 (-10%), H-10→LUMO (-16%), H-10→L+2 (71%) |
| 328.7 | 0 | H-11→L+2 (13%), H-1→L+6 (77%) |
| 327.5 | 0.0125 | H-15→LUMO (-11%), H-10→L+3 (80%) |
| 326.6 | 0 | H-11→L+2 (-39%), H-9→L+3 (41%) |
| 324.9 | 0.0069 | H-24→LUMO (-10%), H-12→L+1 (26%), H-3→L+5 (-12%), H-2→L+6 (13%) |
| 324.6 | 0 | H-11→L+3 (75%), H-5→L+4 (-12%) |
| 323.1 | 0 | H-11→L+3 (13%), H-5→L+4 (68%) |
| 320.9 | 0 | H-17→LUMO (35%), H-14→LUMO (11%), H-13→LUMO (-28%) |
| 320.8 | 0 | H-16→LUMO (-13%), H-14→LUMO (42%), H-14→L+2 (10%), H-13→LUMO (16%) |
| 320.6 | 0 | H-16→LUMO (63%), H-13→LUMO (14%) |
| 320.6 | 0 | H-17→LUMO (42%), H-16→LUMO (-16%), H-14→LUMO (-14%), H-13→LUMO (10%) |
| 319.6 | 0.1031 | H-3→L+5 (82%) |
| 318.7 | 0.0001 | H-4→L+5 (95%) |
| 317.0 | 0 | H-27→LUMO (-10%), H-20→L+1 (10%), H-12→L+2 (38%) |
| 315.8 | 0.0602 | H-1→L+7 (93%) |
| 314.2 | 0.0315 | H-22→LUMO (77%) |
| 313.5 | 0 | H-23→LUMO (75%) |
| 313.2 | 0.0003 | H-20→LUMO (33%), H-12→L+1 (35%), H-2→L+6 (-13%) |
| 311.3 | 0.007 | H-6→L+4 (95%) |
| 310.1 | 0.0001 | H-29→LUMO (66%) |
| 310.1 | 0 | H-30→LUMO (60%) |
| 309.7 | 0.0558 | H-24→LUMO (16%), H-2→L+6 (58%) |
| 306.7 | 0 | H-15→L+1 (94%) |
| 306.2 | 0.23 | H-5→L+5 (75%) |
| 306.0 | 0 | H-7→L+4 (93%) |
| 303.6 | 0.012 | H-19→L+1 (21%), H-18→LUMO (44%), H-18→L+2 (11%) |
| 303.5 | 0 | H-19→LUMO (55%), H-19→L+2 (15%), H-18→L+1 (22%) |
| 302.9 | 0 | H-12→L+2 (32%), H-2→L+7 (20%) |
| 302.7 | 0.0522 | H-28→LUMO (19%), H-27→L+1 (11%), H-5→L+5 (12%), HOMO→L+9 (-13%) |
| 302.6 | 0 | H-14→L+1 (27%), H-14→L+2 (-12%), H-13→L+1 (13%), H-13→L+2 (31%) |
| 302.6 | 0 | H-14→L+1 (-13%), H-14→L+2 (31%), H-13→L+1 (26%), H-13→L+2 (11%) |
| 301.3 | 0.0322 | H-15→LUMO (-14%), H-15→L+2 (74%) |
| 301.1 | 0.0016 | H-17→L+1 (31%), H-16→L+1 (62%) |
| 301.1 | 0.001 | H-17→L+1 (60%), H-16→L+1 (-33%) |

**
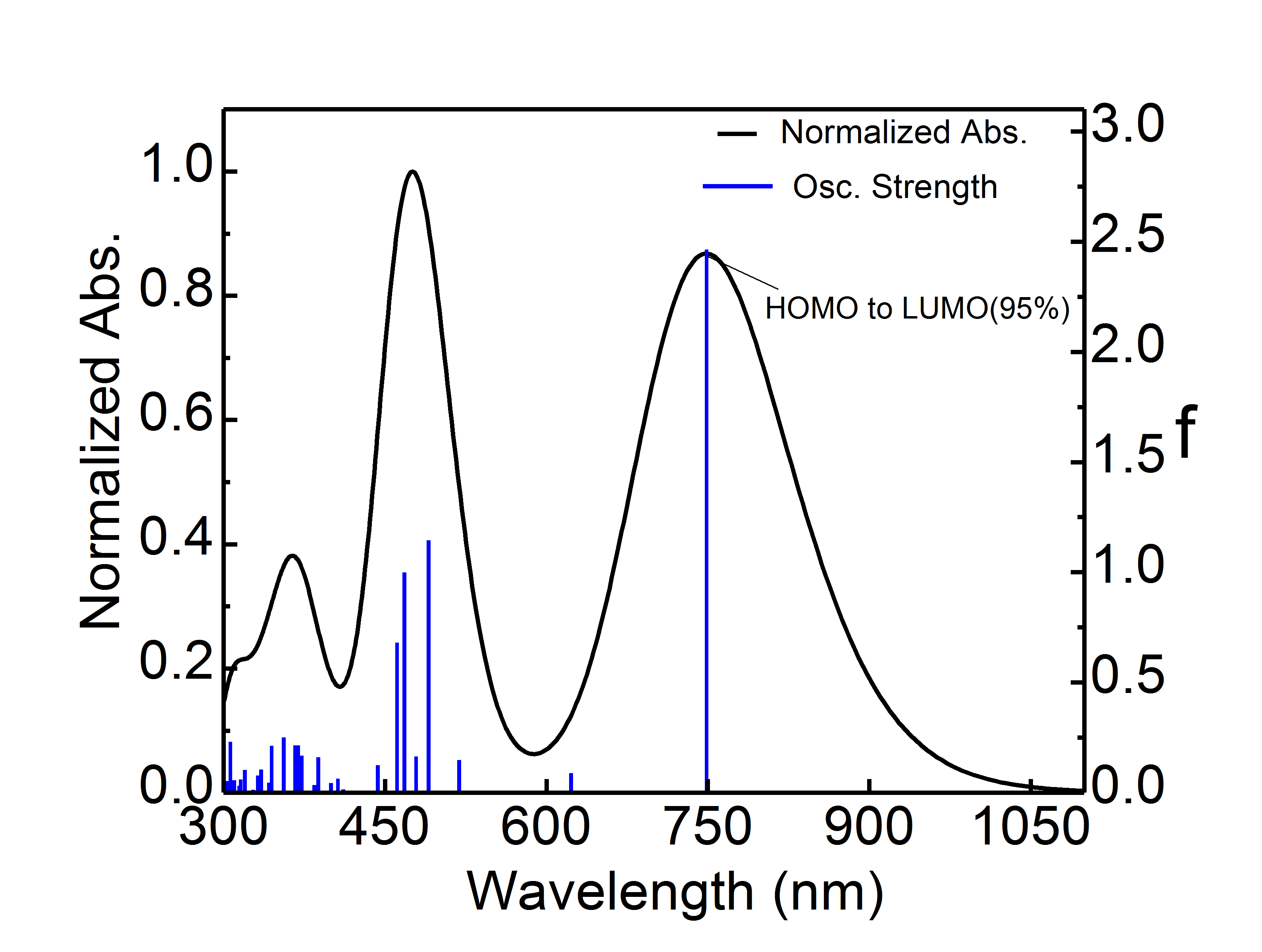
**

# Figure S8.Bar graph (blue) reporting the position of the electronic transitions vs the oscillator strength (f) forAQ-[S]-[Zn]-[S]-AQ.The black line represents a spectrum where 1000 cm-1 is applied to each transition.

# Table S4.Atomic contributions for the frontier MO forAQ-[S]-[Zn]-[S]-AQ.

|  | H-4 | H-3 | H-2 | H-1 | H | L | L+1 | L+2 | L+3 | L+4 |
| --- | --- | --- | --- | --- | --- | --- | --- | --- | --- | --- |
| 2*thiophene-porphyrins | ~1.00 | 0.96 | 0.86 | ~1.00 | 0.96 | 0.60 | 0.09 | 0.43 | ~1.00 | 0.61 |
| anthranquinone | ~0.0 | 0 04 | 0.14 | ~0.0 | 0.04 | 0.40 | 0.91 | 0.57 | ~0.0 | 0.39 |

**AQ-[B-Zn]-AQ**

# Figure S9.Structure of AQ-[B-Zn]-AQ.

**
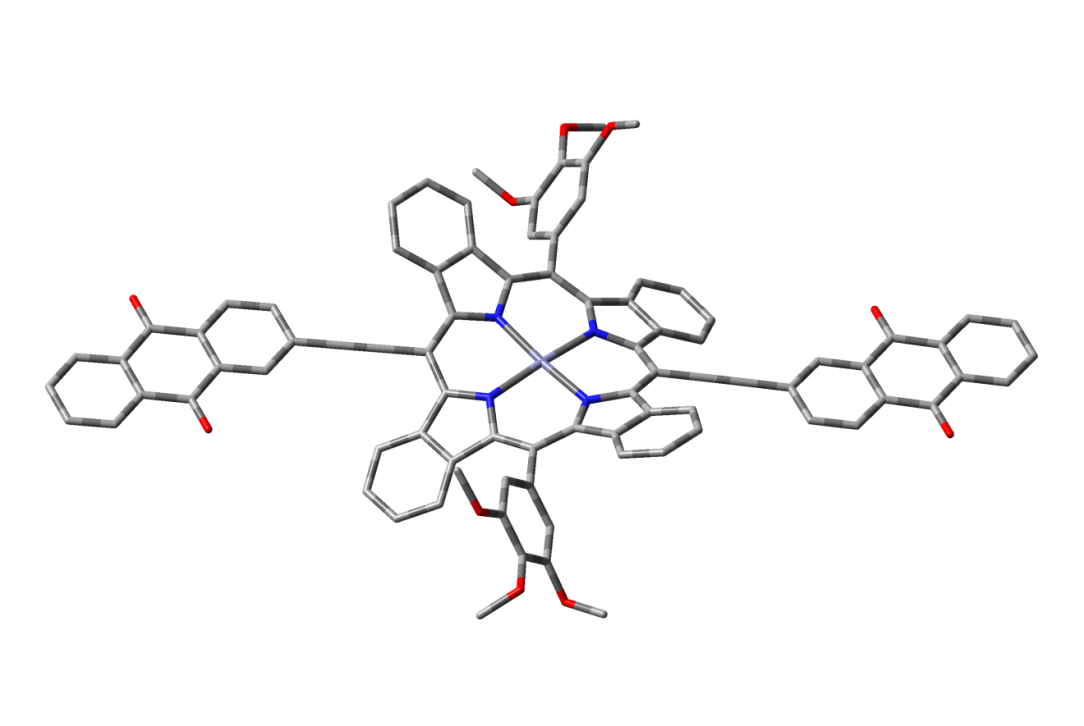
**

# Figure S10. Image of the optimized structure ofAQ-[B-Zn]-AQ.


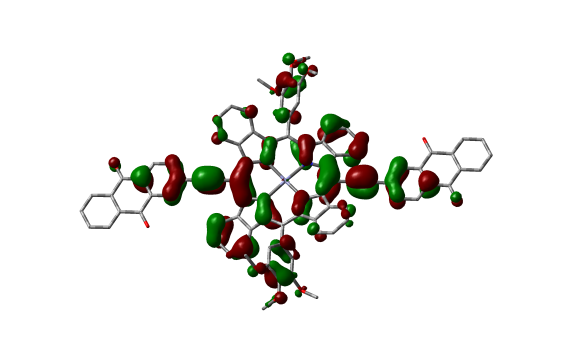


H-4 (-0.21947)


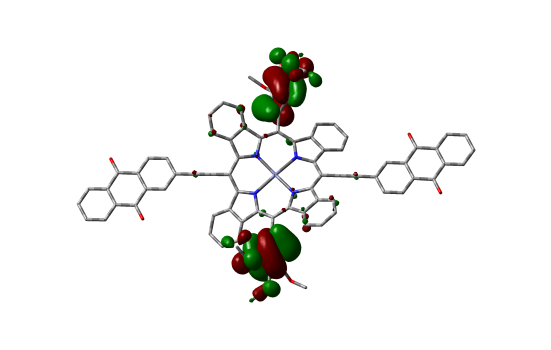


H-3 (-0.21622)


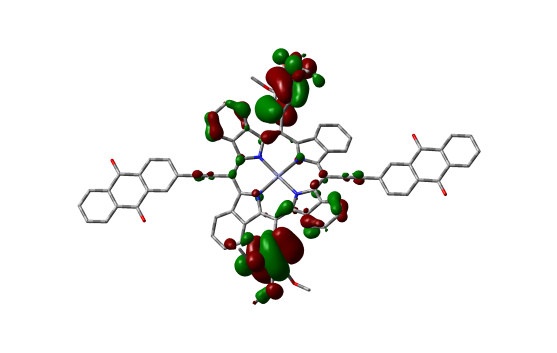


H-2 (-0.21491)


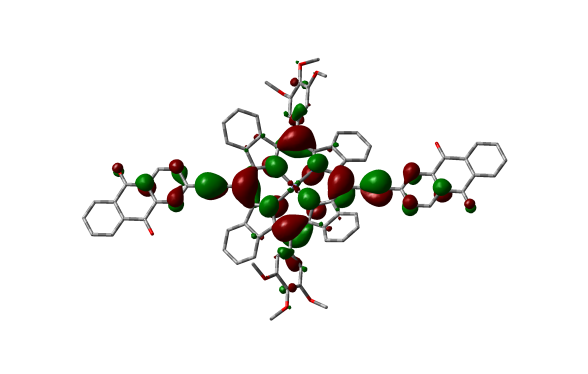


H-1 (-0.18536)


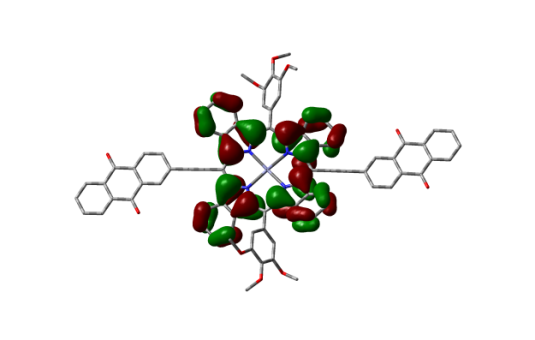


HOMO (-0.17511)


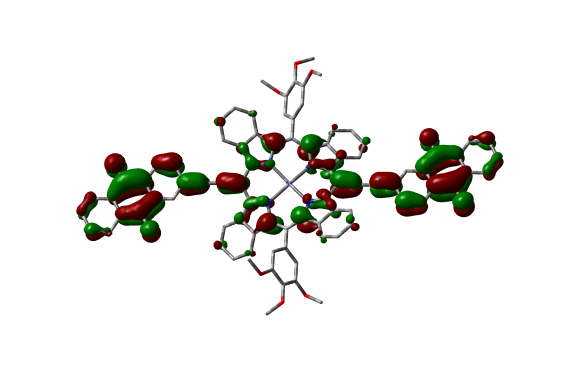


LUMO (-0.10783)


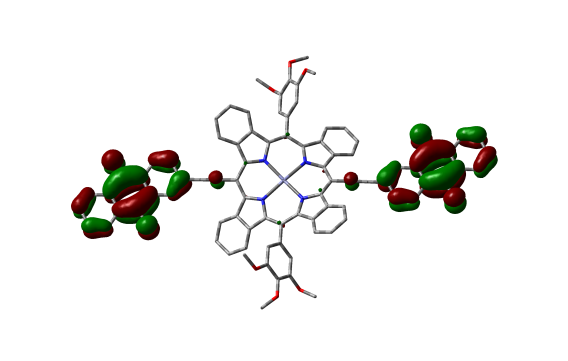


L+1 (-0.10160)


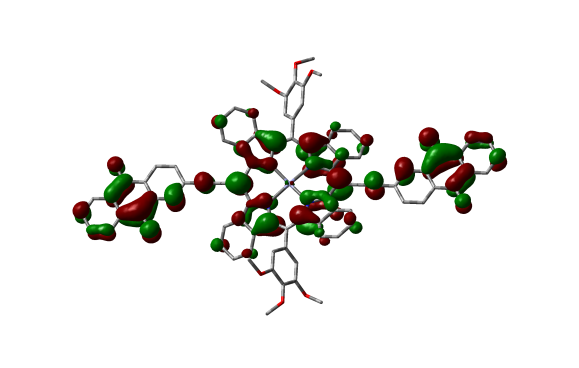


L+2 (-0.09095)


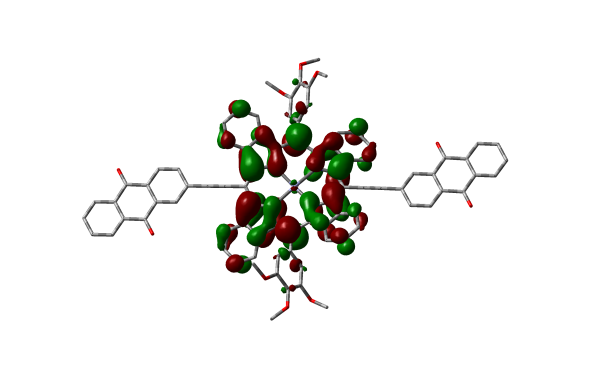


L+3 (-0.08292)


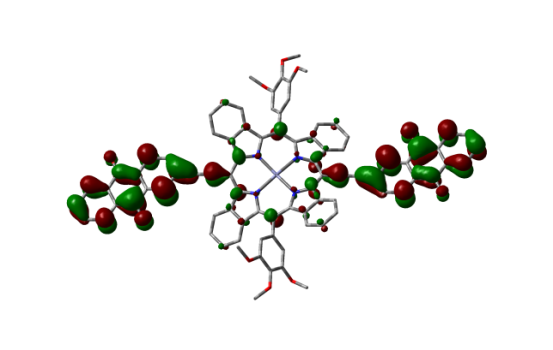


L+4 (-0.06099)

# Figure S11.Representations of the frontier MOs of AQ-[B-Zn]-AQ.The MO energies are inHartree.

# Table S5.Computed positions of the electronic transitions, oscillator strength (f), and major contributions of the AQ-[B-Zn]-AQ.

| Wavelength (nm) | Osc. Strength | Major contribs |
| --- | --- | --- |
| 794.3 | 0.1243 | HOMO→LUMO (96%) |
| 723.1 | 0.0008 | HOMO→L+1 (100%) |
| 700.4 | 0.7058 | H-1→LUMO (84%), HOMO→L+3 (13%) |
| 633.4 | 0.0962 | H-1→L+3 (13%), HOMO→L+2 (86%) |
| 618.3 | 0.0003 | H-1→L+1 (99%) |
| 593.3 | 0.4669 | H-1→LUMO (13%), H-1→L+2 (44%), HOMO→L+3 (-43%) |
| 501.2 | 2.0363 | H-1→L+2 (53%), HOMO→L+3 (41%) |
| 483.7 | 0.9802 | H-1→L+3 (77%), HOMO→L+2 (-14%) |
| 478.9 | 0.0011 | H-2→LUMO (95%) |
| 471.3 | 0.0734 | H-3→LUMO (92%) |
| 460.8 | 0.0001 | H-4→LUMO (94%) |
| 447.5 | 0.012 | HOMO→L+4 (98%) |
| 438.0 | 0.0034 | H-2→L+1 (90%) |
| 430.8 | 0.0004 | H-3→L+1 (95%) |
| 427.0 | 0.0085 | H-6→LUMO (-14%), H-4→L+1 (71%) |
| 423.2 | 0.0033 | H-5→LUMO (89%) |
| 421.1 | 0.0001 | H-15→LUMO (31%), H-14→L+1 (34%), H-6→LUMO (19%) |
| 420.9 | 0 | H-15→L+1 (43%), H-14→LUMO (40%), H-14→L+2 (10%) |
| 420.1 | 0.0016 | H-14→L+1 (-10%), H-6→LUMO (59%), H-4→L+1 (13%) |
| 417.0 | 0.1747 | HOMO→L+5 (95%) |
| 410.2 | 0.0007 | H-2→L+2 (10%), H-1→L+4 (83%) |
| 407.2 | 0.0004 | H-2→L+2 (79%), H-1→L+4 (-11%) |
| 405.4 | 0.0002 | H-7→LUMO (76%) |
| 401.9 | 0.0068 | H-3→L+2 (93%) |
| 397.3 | 0.0033 | H-4→L+2 (28%), H-2→L+3 (60%) |
| 394.9 | 0.0015 | H-4→L+2 (50%), H-2→L+3 (-32%) |
| 392.6 | 0.0003 | H-9→LUMO (12%), H-8→LUMO (-14%), H-3→L+3 (65%) |
| 391.6 | 0 | H-9→LUMO (-30%), H-8→LUMO (28%), H-3→L+3 (31%) |
| 389.9 | 0.0052 | H-5→L+1 (88%) |
| 387.7 | 0.0003 | H-6→L+1 (85%) |
| 386.0 | 0 | H-10→LUMO (69%) |
| 385.5 | 0.0031 | H-25→LUMO (19%), H-24→L+1 (20%), H-9→LUMO (15%), H-8→LUMO (12%), H-1→L+5 (-16%) |
| 384.9 | 0 | H-25→L+1 (35%), H-24→LUMO (26%), H-10→LUMO (13%) |
| 384.9 | 0.0097 | H-25→LUMO (17%), H-24→L+1 (16%), H-1→L+5 (31%) |
| 382.2 | 0.0353 | H-9→LUMO (20%), H-8→LUMO (20%), H-1→L+5 (39%) |
| 381.9 | 0.0015 | H-4→L+3 (75%), HOMO→L+6 (-14%) |
| 377.6 | 0.0044 | H-11→LUMO (49%), HOMO→L+6 (38%) |
| 373.8 | 0.0264 | H-13→LUMO (72%), H-13→L+2 (-16%) |
| 372.7 | 0.0571 | H-11→LUMO (-32%), HOMO→L+6 (38%) |
| 369.1 | 0.037 | H-12→LUMO (30%), H-7→L+1 (54%) |
| 368.1 | 0.0548 | H-12→LUMO (43%), H-7→L+1 (-40%) |
| 365.6 | 0.0147 | H-5→L+2 (86%) |
| 365.0 | 0.0046 | H-6→L+2 (92%) |
| 358.2 | 0.0003 | H-8→L+1 (27%), H-5→L+3 (58%) |
| 357.8 | 0.0137 | H-6→L+3 (95%) |
| 357.3 | 0.0005 | H-8→L+1 (48%), H-5→L+3 (-28%) |
| 356.1 | 0.0016 | H-9→L+1 (72%) |
| 354.2 | 0 | H-1→L+6 (36%), HOMO→L+8 (49%) |
| 351.8 | 0.0143 | H-10→L+1 (77%) |
| 348.0 | 0.0094 | H-7→L+2 (71%) |
| 347.3 | 0.0066 | H-11→L+1 (78%), H-9→L+2 (12%) |
| 346.0 | 0.0001 | H-1→L+6 (44%), HOMO→L+8 (-32%) |
| 343.3 | 0.0013 | H-17→LUMO (46%), H-16→L+1 (14%) |
| 342.9 | 0 | H-17→L+1 (15%), H-16→LUMO (52%) |
| 342.2 | 0.0111 | HOMO→L+7 (89%) |
| 341.8 | 0 | H-12→L+1 (27%), H-10→L+2 (32%), H-7→L+3 (26%) |
| 337.7 | 0.0011 | H-10→L+3 (11%), H-1→L+8 (-10%), HOMO→L+9 (69%) |
| 337.4 | 0.005 | H-11→L+1 (13%), H-9→L+2 (-20%), H-8→L+2 (49%) |
| 336.9 | 0.0002 | H-12→L+1 (48%), H-7→L+3 (-34%) |
| 336.4 | 0.0063 | H-9→L+2 (52%), H-8→L+2 (24%) |
| 334.8 | 0.0444 | H-9→L+3 (-30%), H-8→L+3 (37%) |
| 332.9 | 0.0344 | H-13→L+3 (81%) |
| 332.1 | 0.0003 | H-22→LUMO (-20%), H-18→LUMO (-14%), H-13→L+1 (29%) |
| 331.7 | 0.1556 | H-23→LUMO (12%), H-19→LUMO (41%), H-18→L+1 (25%) |
| 331.3 | 0.0003 | H-19→L+1 (15%), H-18→LUMO (26%), H-13→L+1 (36%) |
| 330.6 | 0.0065 | H-11→L+2 (43%), H-10→L+3 (34%), HOMO→L+9 (-10%) |
| 330.3 | 0.0002 | H-13→L+1 (14%), H-12→L+1 (-16%), H-10→L+2 (32%) |
| 328.9 | 0.0247 | H-23→LUMO (19%), H-19→LUMO (-12%), H-17→LUMO (14%) |
| 328.8 | 0 | H-22→LUMO (13%), H-18→LUMO (-13%), H-16→LUMO (17%) |
| 326.7 | 0.0577 | H-11→L+2 (17%), H-1→L+8 (59%) |
| 326.2 | 0.0004 | H-26→LUMO (14%), H-14→LUMO (16%), H-13→L+1 (-13%), H-10→L+2 (12%) |
| 324.6 | 0.0012 | H-30→LUMO (16%), H-15→LUMO (-15%), HOMO→L+10 (21%) |
| 323.6 | 0.0132 | H-11→L+2 (-28%), H-10→L+3 (47%), H-1→L+8 (19%) |
| 323.1 | 0.0009 | HOMO→L+10 (61%) |
| 322.6 | 0.0049 | H-13→L+3 (-11%), H-9→L+3 (29%), H-8→L+3 (43%) |
| 320.5 | 0.0017 | H-12→L+2 (53%), H-1→L+7 (-18%) |
| 320.1 | 0.0006 | H-11→L+3 (63%), H-1→L+9 (-15%) |
| 319.5 | 0.0231 | H-15→LUMO (-11%), H-13→LUMO (10%), H-13→L+2 (54%) |
| 319.0 | 0.0914 | H-12→L+2 (26%), H-2→L+4 (-10%), H-1→L+7 (37%) |
| 318.6 | 0.0001 | H-26→LUMO (13%), H-17→L+1 (19%), H-16→LUMO (-11%), H-15→L+1 (15%), H-14→LUMO (-17%) |
| 317.9 | 0.0728 | H-2→L+4 (60%) |
| 317.0 | 0.0154 | H-20→LUMO (44%) |
| 316.5 | 0 | H-29→LUMO (12%), H-21→LUMO (11%), H-17→L+1 (20%), H-14→LUMO (14%) |
| 315.9 | 0.0411 | H-16→L+1 (20%), H-1→L+7 (18%) |
| 315.0 | 0.0001 | H-21→LUMO (78%) |
| 314.7 | 0.1035 | H-12→L+3 (87%) |
| 314.5 | 0 | H-3→L+4 (92%) |
| 313.3 | 0.0338 | HOMO→L+11 (83%) |
| 312.8 | 0.0285 | H-30→LUMO (-22%), H-20→LUMO (38%) |
| 312.2 | 0.0503 | HOMO→L+12 (85%) |
| 311.5 | 0.1595 | H-4→L+4 (64%) |
| 310.8 | 0.0003 | H-11→L+3 (16%), H-1→L+9 (66%) |
| 305.6 | 0.0059 | H-27→LUMO (86%) |
| 303.4 | 0.0001 | H-2→L+5 (79%) |
| 302.2 | 0.0917 | H-28→LUMO (-15%), H-1→L+10 (45%) |
| 302.0 | 0.0002 | H-29→LUMO (22%), H-19→L+1 (11%), H-17→L+1 (-14%) |
| 301.9 | 0.0089 | H-19→LUMO (-10%), H-18→L+1 (14%), H-1→L+10 (44%) |
| 301.7 | 0 | H-19→L+1 (37%), H-18→LUMO (-29%) |
| 301.6 | 0.0248 | H-28→LUMO (-14%), H-19→LUMO (-22%), H-18→L+1 (28%) |

**
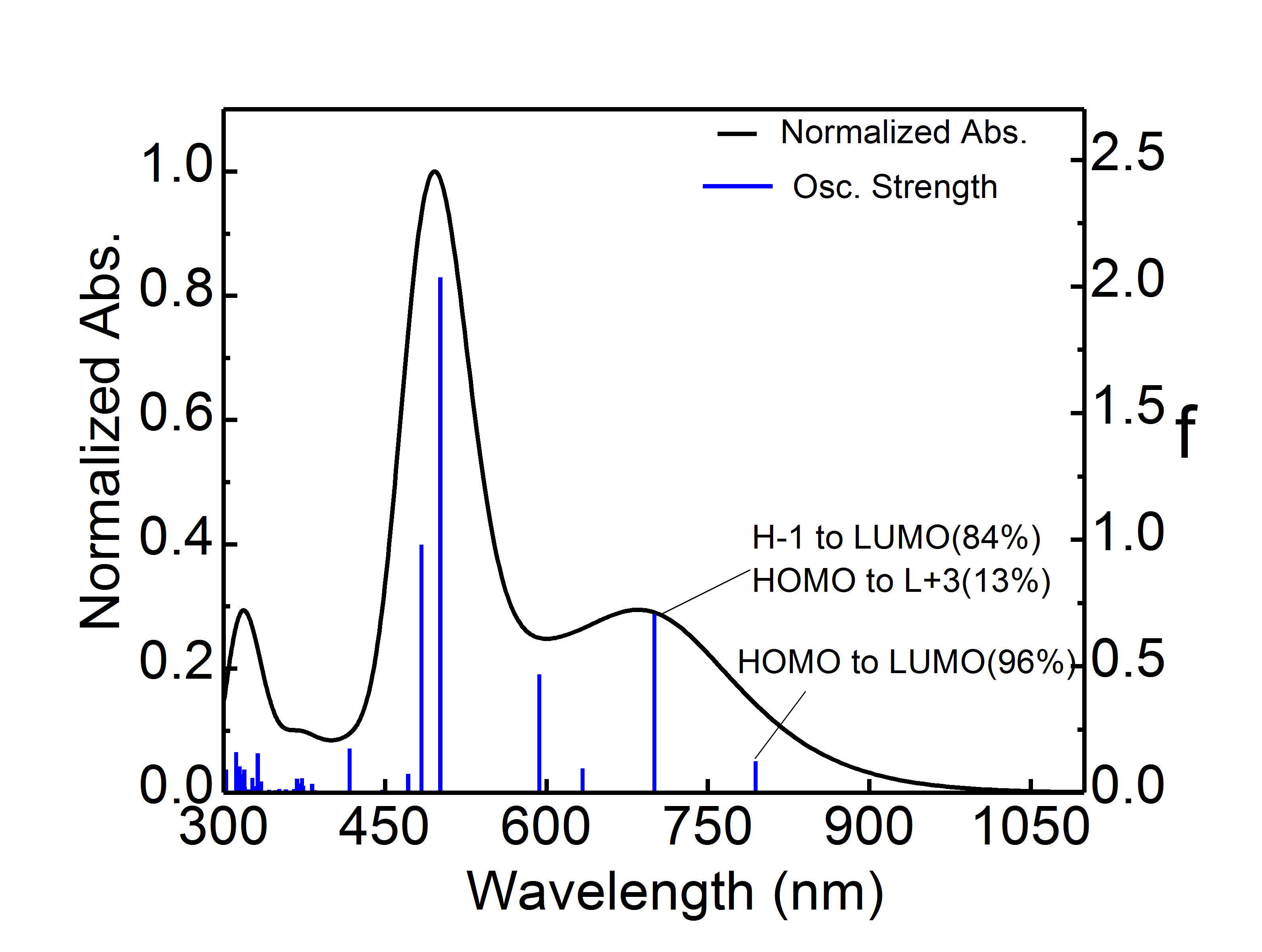
**

# Figure S12.Bar graph (blue) reporting the position of the electronic transitions vs the oscillator strength (f) for AQ-[B-Zn]-AQ.The black line represents a spectrum where 1000 cm-1 is applied to each transition.

# Table S6.Atomic contributions for the frontier MO for AQ-[B-Zn]-AQ.

|  | H-4 | H-3 | H-2 | H-1 | H | L | L+1 | L+2 | L+3 | L+4 |
| --- | --- | --- | --- | --- | --- | --- | --- | --- | --- | --- |
| Ethyne-benzo-porphyrins | ~1.00 | 0.96 | 0.86 | ~1.00 | 0.96 | 0.60 | 0.09 | 0.43 | ~1.00 | 0.61 |
| 2*anthranquinone | ~0.0 | 0 04 | 0.14 | ~0.0 | 0.04 | 0.40 | 0.91 | 0.57 | ~0.0 | 0.39 |

**AQ-[S]-[B-Zn]-[S]-AQ**

# Figure S13.Structure of AQ-[S]-[B-Zn]-[S]-AQ.


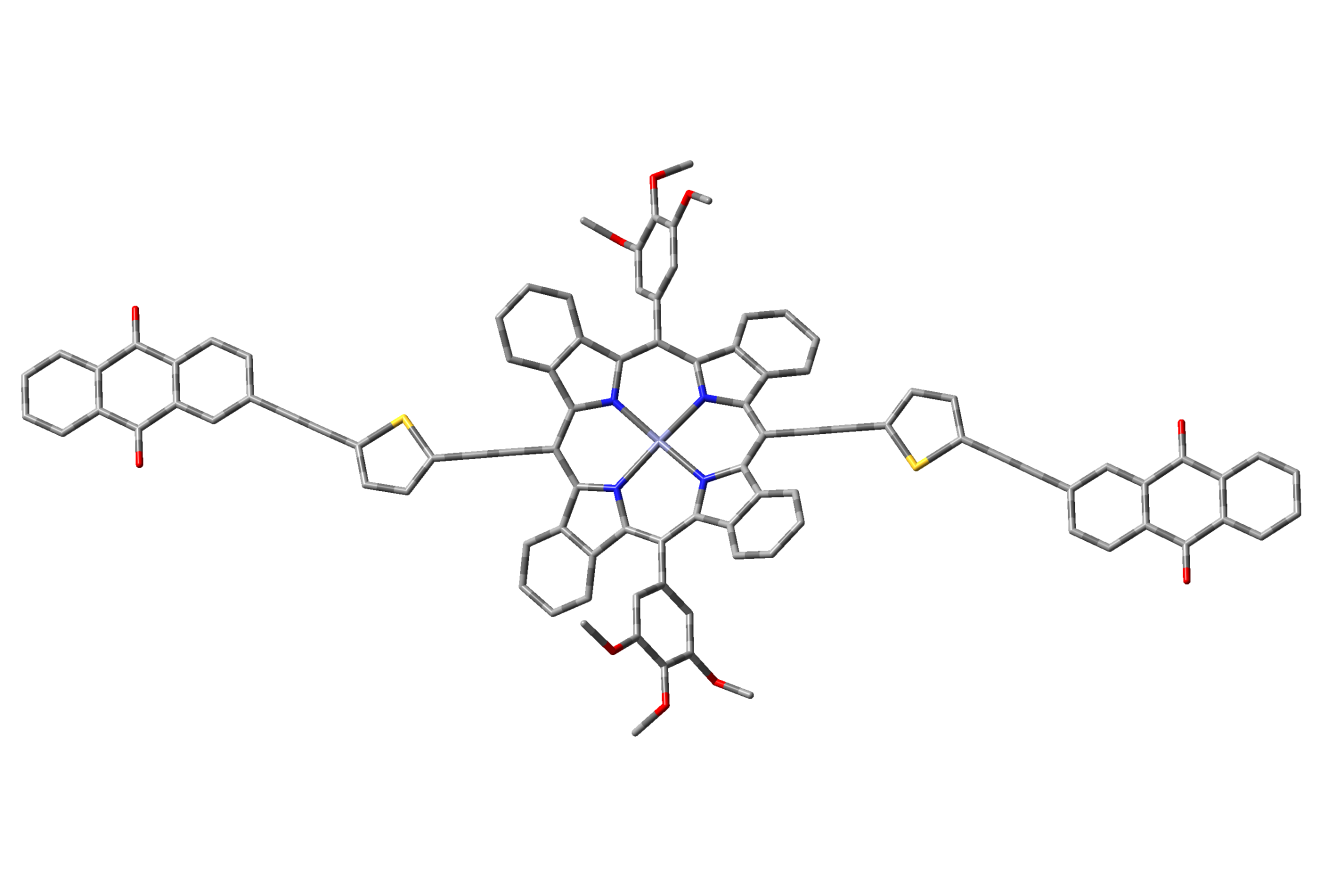


# Figure S14.Image of the optimized structure ofAQ-[S]-[B-Zn]-[S]-AQ.

**
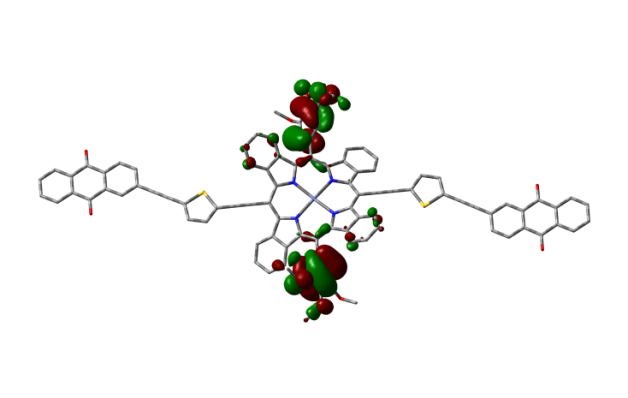
**

H-4 (-0.21537)

**
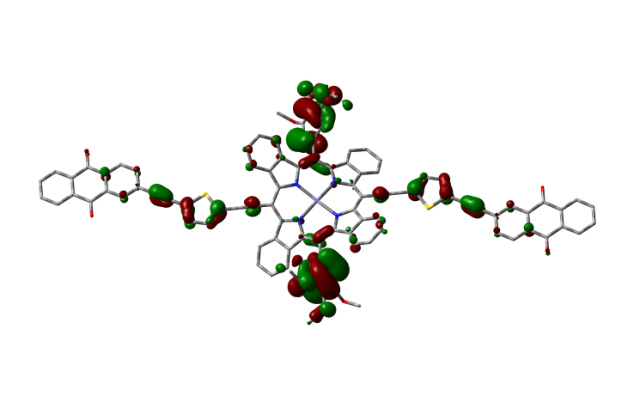
**

H-3 (-0.21448)

**
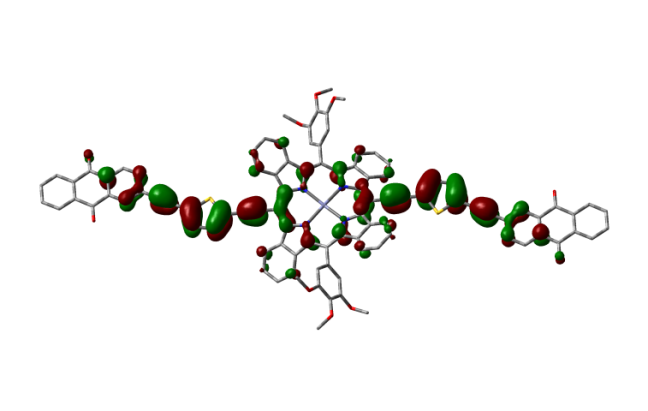
**

H-2 (-0.20338)

**
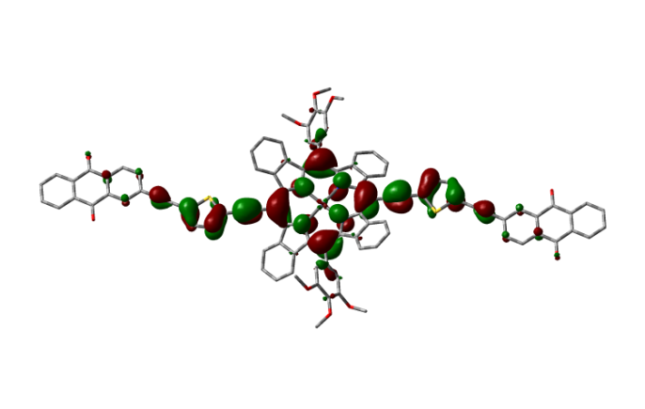
**

H-1 (-0.18002)


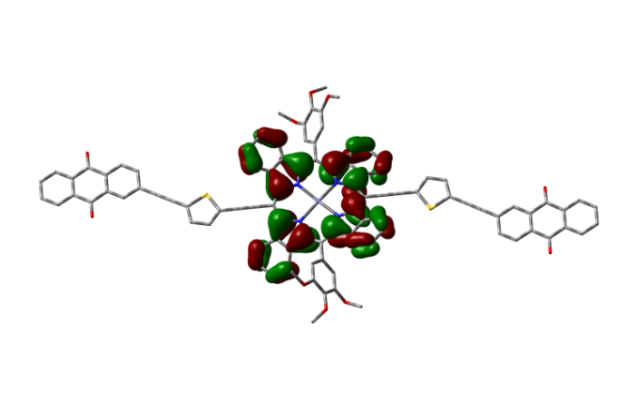


HOMO (-0.17506)


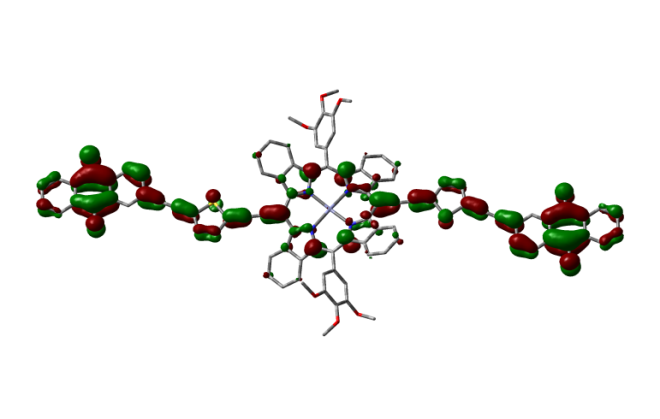


LUMO (-0.10683)

**
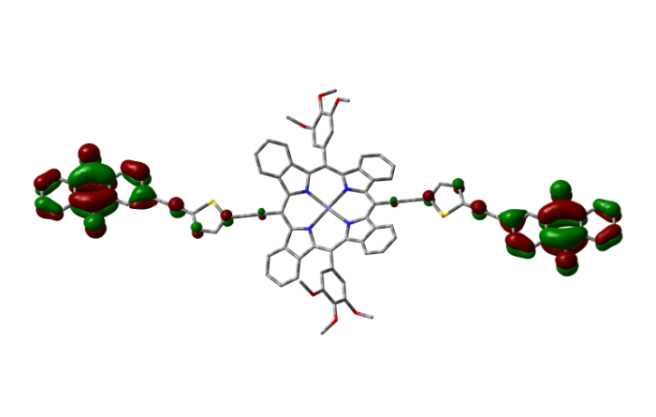
**

L+1 (-0.10370)

**
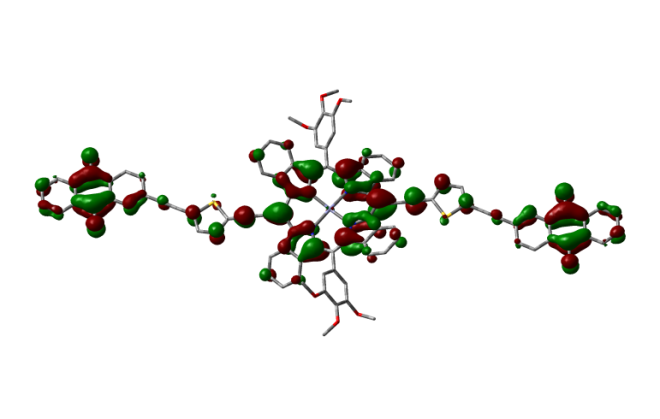
**

L+2 (-0.09665)

**
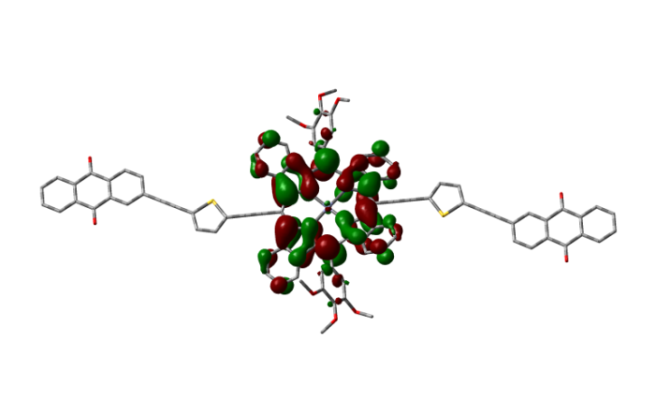
**

L+3 (-0.08283)

**
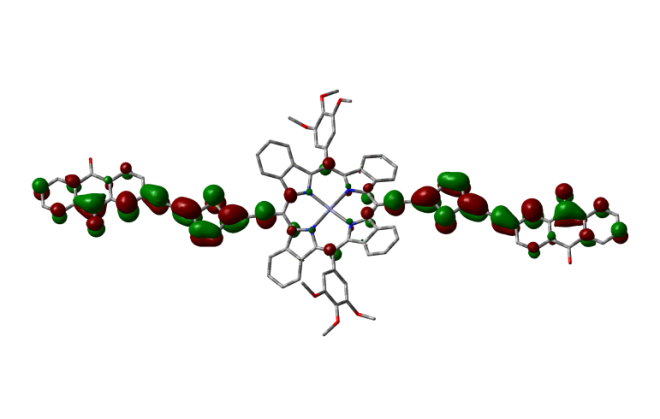
**

L+4 (-0.07356)

# Figure S15.Representations of the frontier MOs of AQ-[S]-[B-Zn]-[S]-AQ.The MO energies are in Hartree.

# Table S7.Computed positions of the electronic transitions, oscillator strength (f), and major contributions of the AQ-[S]-[B-Zn]-[S]-AQ.

| Wavelength (nm) | Osc. Strength | Major contribs |
| --- | --- | --- |
| 763.9 | 0.1239 | HOMO→LUMO (89%) |
| 732.1 | 1.6409 | H-1→LUMO (84%) |
| 708.0 | 0.0004 | HOMO→L+1 (100%) |
| 663.3 | 0.0645 | H-1→L+3 (10%), HOMO→L+2 (81%) |
| 660.3 | 0.0002 | H-1→L+1 (99%) |
| 631.1 | 0.0821 | H-1→LUMO (13%), H-1→L+2 (64%), HOMO→L+3 (-22%) |
| 545.9 | 2.8724 | H-1→L+2 (26%), HOMO→L+3 (58%) |
| 529.3 | 0.0004 | H-2→LUMO (93%) |
| 515.2 | 0.6619 | H-1→L+3 (75%), HOMO→L+2 (-14%) |
| 503.7 | 0.007 | HOMO→L+4 (98%) |
| 496.6 | 0.0566 | H-2→L+1 (88%) |
| 476.7 | 0.0002 | H-1→L+4 (93%) |
| 471.8 | 0 | H-2→L+2 (86%) |
| 467.4 | 0.0063 | H-3→LUMO (81%), H-3→L+2 (-12%) |
| 465.4 | 0.0007 | H-4→LUMO (81%), H-4→L+2 (-15%) |
| 454.7 | 0.379 | HOMO→L+5 (89%) |
| 442.9 | 0.008 | H-5→LUMO (50%), H-1→L+5 (-39%) |
| 437.8 | 0.0001 | H-3→L+1 (94%) |
| 432.5 | 0.0025 | H-4→L+1 (98%) |
| 429.4 | 0.0046 | H-2→L+3 (85%) |
| 426.6 | 0.0461 | H-5→LUMO (20%), H-3→L+2 (28%), H-1→L+5 (34%) |
| 420.7 | 0 | H-17→L+1 (46%), H-16→LUMO (35%), H-16→L+2 (13%) |
| 420.7 | 0 | H-17→LUMO (35%), H-17→L+2 (13%), H-16→L+1 (46%) |
| 419.6 | 0.0111 | H-5→LUMO (-17%), H-3→L+2 (55%), H-1→L+5 (-13%) |
| 418.5 | 0.0012 | H-4→LUMO (13%), H-4→L+2 (76%) |
| 417.8 | 0.0058 | H-6→LUMO (69%), H-6→L+2 (-12%) |
| 413.9 | 0.001 | H-5→L+1 (76%) |
| 407.7 | 0.0353 | H-7→LUMO (70%), H-7→L+2 (-10%) |
| 398.0 | 0.0063 | H-8→LUMO (-19%), HOMO→L+6 (69%) |
| 397.5 | 0.0076 | H-5→L+2 (71%) |
| 396.3 | 0.0189 | H-8→LUMO (50%), H-8→L+2 (-14%), HOMO→L+6 (23%) |
| 395.8 | 0.0526 | H-3→L+3 (91%) |
| 395.0 | 0 | H-4→L+3 (93%) |
| 390.5 | 0.0031 | H-6→L+1 (93%) |
| 387.9 | 0 | H-9→LUMO (62%), H-7→L+1 (12%) |
| 385.1 | 0.0006 | H-10→LUMO (70%), H-10→L+2 (-23%) |
| 385.1 | 0 | H-28→LUMO (17%), H-28→L+1 (20%), H-27→LUMO (14%), H-27→L+1 (23%) |
| 385.1 | 0 | H-28→LUMO (-14%), H-28→L+1 (24%), H-27→LUMO (16%), H-27→L+1 (-19%) |
| 383.5 | 0 | H-7→L+1 (-22%), H-1→L+6 (57%) |
| 382.2 | 0.0875 | HOMO→L+7 (86%) |
| 382.1 | 0.0003 | H-7→L+1 (61%), H-1→L+6 (31%) |
| 381.9 | 0.5007 | H-2→L+4 (82%) |
| 379.3 | 0.0016 | H-6→LUMO (16%), H-6→L+2 (79%) |
| 372.8 | 0.0146 | H-7→LUMO (11%), H-7→L+2 (67%) |
| 370.2 | 0.0019 | H-12→LUMO (22%), H-11→LUMO (39%) |
| 369.9 | 0.0497 | H-15→LUMO (33%), H-15→L+2 (-22%), H-1→L+7 (-32%) |
| 368.7 | 0.0054 | H-12→LUMO (46%), H-12→L+2 (-12%), H-11→LUMO (-17%) |
| 367.8 | 0.1032 | H-8→L+1 (41%), H-5→L+3 (31%) |
| 366.7 | 0.0652 | H-13→LUMO (-15%), H-5→L+3 (32%), H-1→L+7 (-22%) |
| 366.3 | 0.0192 | H-15→LUMO (-13%), H-8→L+1 (19%), H-5→L+3 (-11%), H-1→L+7 (-17%) |
| 364.3 | 0.0824 | H-14→LUMO (-11%), H-9→L+1 (32%), H-8→L+1 (-31%) |
| 360.9 | 0.0051 | H-6→L+3 (85%) |
| 360.7 | 0.0245 | H-14→LUMO (44%), H-14→L+2 (-12%) |
| 358.4 | 0.0001 | H-8→L+2 (16%), H-2→L+5 (56%) |
| 358.3 | 0.0717 | H-13→LUMO (36%), H-9→L+1 (-33%) |
| 357.9 | 0.0028 | H-8→LUMO (12%), H-8→L+2 (50%), H-2→L+5 (-12%) |
| 356.6 | 0.033 | HOMO→L+8 (65%) |
| 353.3 | 0.0041 | H-10→L+1 (19%), H-9→L+2 (34%), HOMO→L+9 (-26%) |
| 352.4 | 0.0041 | H-10→L+1 (19%), H-1→L+8 (14%), HOMO→L+9 (47%) |
| 351.3 | 0.0053 | H-10→L+1 (41%), H-9→L+2 (-27%) |
| 351.2 | 0.0185 | H-7→L+3 (81%) |
| 346.6 | 0.0013 | H-12→L+1 (-18%), H-11→L+1 (25%), H-10→LUMO (10%), H-10→L+2 (35%) |
| 345.9 | 0 | H-3→L+4 (75%) |
| 345.7 | 0.0004 | H-11→L+1 (54%), H-10→L+2 (-18%) |
| 343.7 | 0 | H-13→L+1 (45%), H-11→L+2 (-20%) |
| 343.2 | 0.0064 | H-4→L+4 (93%) |
| 342.0 | 0.0032 | H-12→L+1 (73%), H-10→L+2 (15%) |
| 339.6 | 0.003 | H-8→L+3 (13%), H-1→L+8 (40%) |
| 338.6 | 0.0163 | H-9→L+3 (-20%), H-1→L+9 (29%) |
| 338.0 | 0.0022 | H-13→L+1 (20%), H-8→L+3 (36%) |
| 335.9 | 0.0031 | H-14→L+1 (31%), H-8→L+3 (20%) |
| 335.8 | 0.119 | H-26→L+1 (-12%), H-23→LUMO (24%), H-22→L+1 (-14%) |
| 334.9 | 0.0186 | H-12→LUMO (15%), H-12→L+2 (51%), H-1→L+9 (13%) |
| 334.3 | 0.0001 | H-23→L+1 (18%), H-22→LUMO (-11%), H-14→L+1 (28%) |
| 334.1 | 0.0003 | H-13→L+2 (-12%), H-10→L+3 (60%) |
| 332.7 | 0.0063 | H-9→L+3 (28%), H-1→L+9 (28%), HOMO→L+11 (-17%) |
| 332.4 | 0.0002 | H-14→L+1 (22%), H-11→L+2 (23%), H-5→L+4 (-12%) |
| 332.2 | 0.0549 | H-13→L+2 (50%), H-10→L+3 (13%) |
| 331.3 | 0.1277 | H-21→LUMO (40%), H-20→L+1 (-39%) |
| 331.0 | 0.0001 | H-21→L+1 (-32%), H-20→LUMO (33%) |
| 330.1 | 0.0001 | H-9→L+3 (19%), HOMO→L+11 (56%) |
| 329.9 | 0.0658 | H-15→L+3 (91%) |
| 329.3 | 0.0005 | H-5→L+4 (63%) |
| 326.9 | 0.0824 | H-14→L+2 (-23%), H-3→L+5 (-23%), HOMO→L+10 (27%) |
| 326.7 | 0.0556 | H-3→L+5 (22%), HOMO→L+10 (46%), HOMO→L+14 (-11%) |
| 326.5 | 0.0214 | H-14→LUMO (10%), H-14→L+2 (39%), H-3→L+5 (-28%) |
| 326.1 | 0 | H-15→L+1 (94%) |
| 324.1 | 0 | H-4→L+5 (95%) |
| 323.7 | 0.0394 | H-10→L+3 (13%), HOMO→L+12 (76%) |
| 322.6 | 0.0137 | H-3→L+5 (-13%), H-2→L+6 (35%) |
| 320.6 | 0.0024 | H-15→LUMO (32%), H-15→L+2 (49%) |
| 320.3 | 0.0011 | H-12→L+3 (29%), H-1→L+11 (28%) |
| 318.4 | 0.0009 | H-30→LUMO (17%), H-29→L+1 (-10%), H-22→LUMO (10%) |
| 317.7 | 0.054 | H-11→L+3 (80%) |
| 317.3 | 0.0071 | H-6→L+4 (75%) |
| 317.1 | 0.0002 | H-30→LUMO (24%), H-1→L+11 (-13%) |
| 316.2 | 0.0146 | H-14→L+3 (40%), H-13→L+3 (-21%), H-1→L+12 (29%) |
| 315.8 | 0.0259 | H-6→L+4 (12%), H-1→L+10 (22%) |
| 315.2 | 0.0004 | H-12→L+3 (44%), H-1→L+11 (-31%) |

**
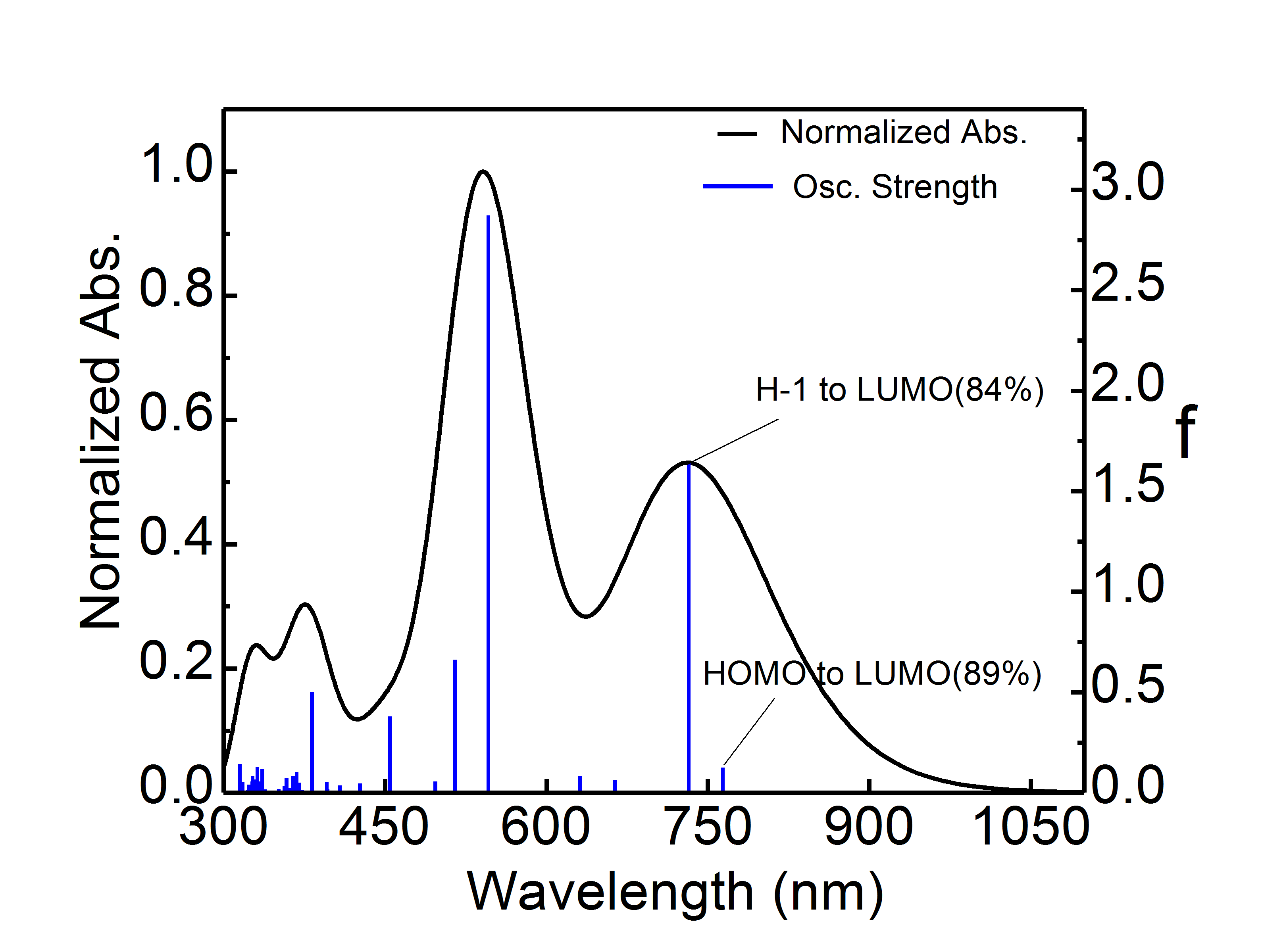
**

# Figure S16. Bar graph (blue) reporting the position of the electronic transitions vs the oscillator strength (f) for AQ-[S]-[B-Zn]-[S]-AQ.The black line represents a spectrum where 1000 cm-1 is applied to each transition.

# Table S8.Atomic contributions for the frontier MO for AQ-[S]-[B-Zn]-[S]-AQ.

|  | H-4 | H-3 | H-2 | H-1 | H | L | L+1 | L+2 | L+3 | L+4 |
| --- | --- | --- | --- | --- | --- | --- | --- | --- | --- | --- |
| 2*thiophene-benzo-porphyrins | ~1.00 | 0.96 | 0.88 | 0.96 | ~1.00 | 0.42 | 0.10 | 0.63 | ~1.00 | 0.62 |
| 2*anthranquinone | ~0.0 | 0.04 | 0.12 | 0.04 | ~0.0 | 0.58 | 0.90 | 0.37 | ~0.0 | 0.38 |

**NQ-[Zn]-NQ**

# Figure S17.Structure of NQ-[Zn]-NQ.


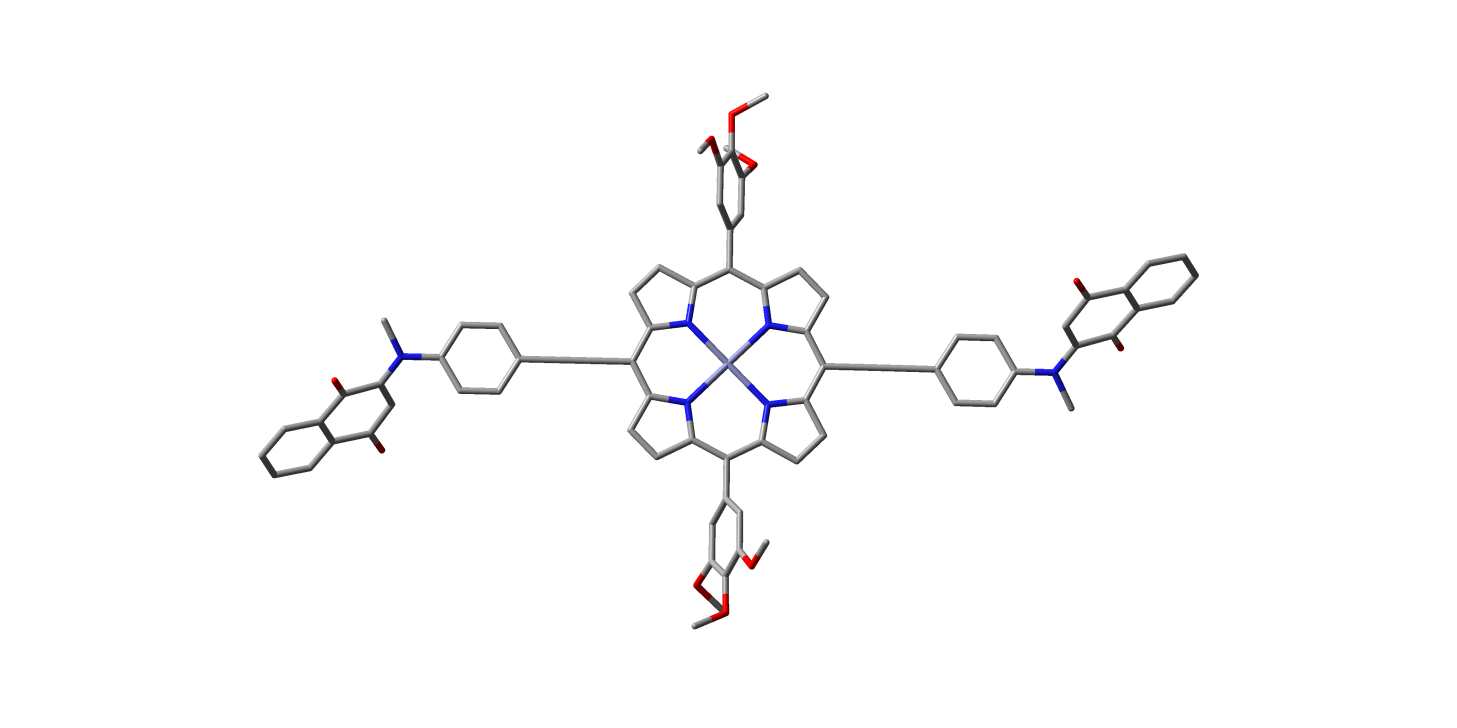


# Figure S18.Image of the optimized structure ofNQ-[Zn]-NQ.


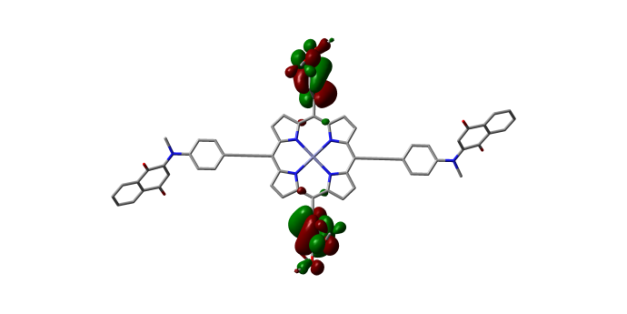


H-4 (-0.22199)


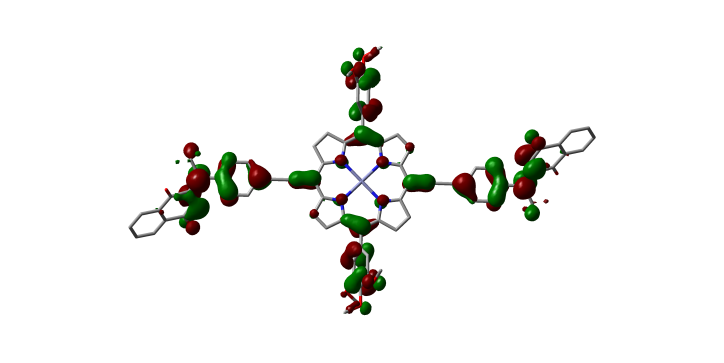


H-3 (-0.21712)


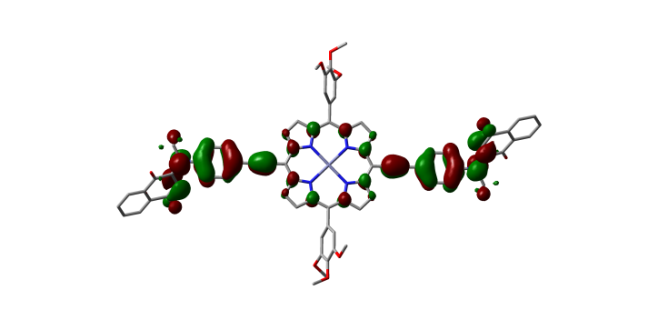


H-2 (-0.20605)


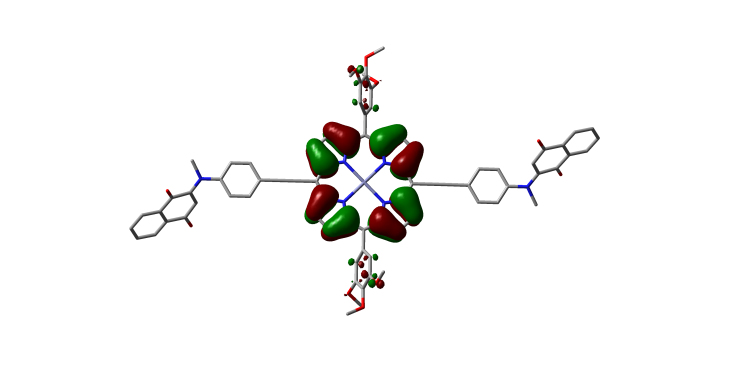


H-1 (-0.19991)


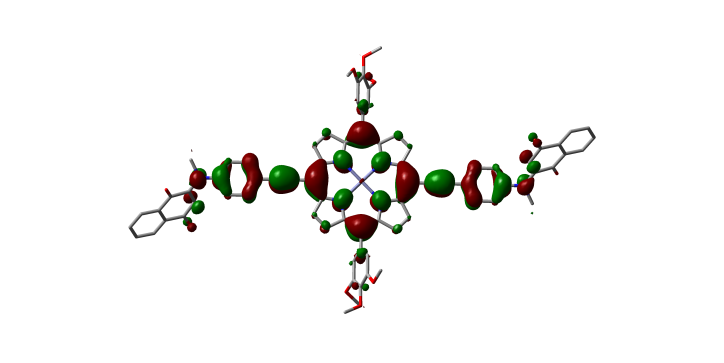


HOMO (-0.18054)


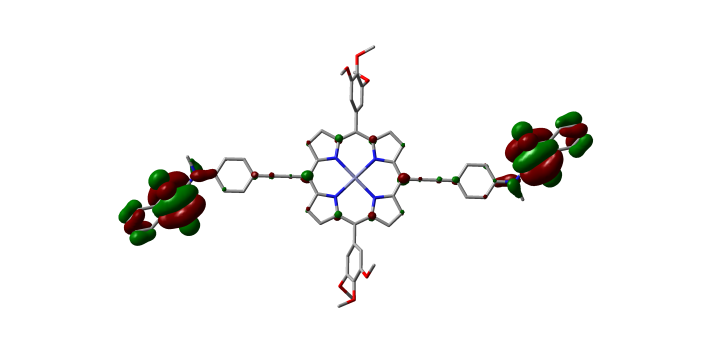


LUMO (-0.11329)


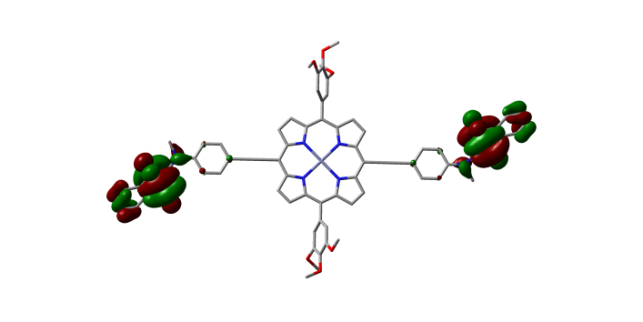


L+1 (-0.11213)


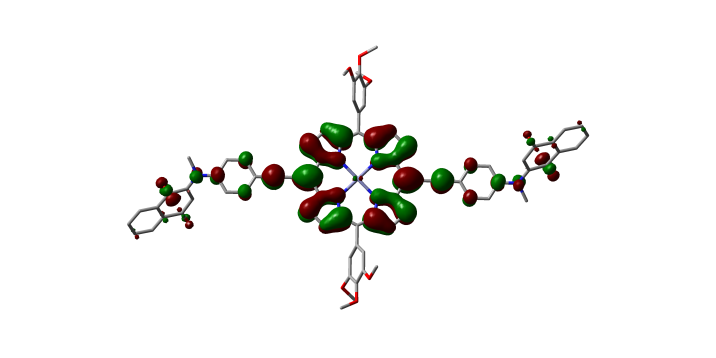


L+2 (-0.09887)


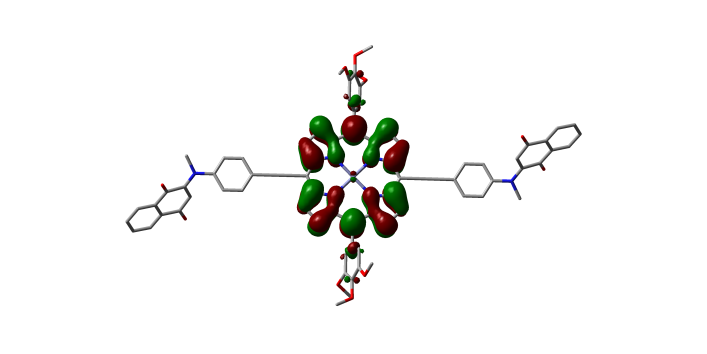


L+3 (-0.08713)


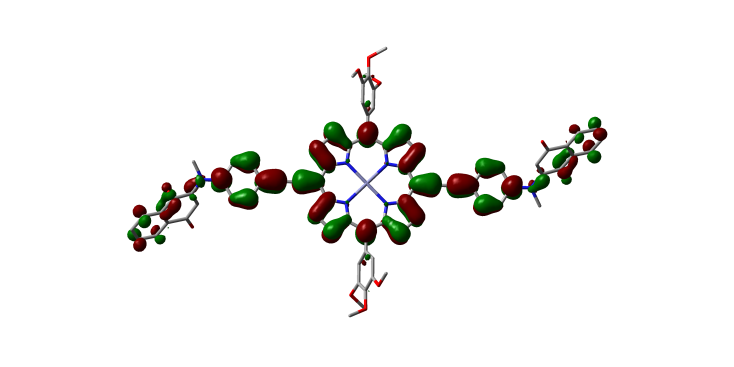


L+4 (-0.05050)

# Figure S19.Representations of the frontier MOs ofNQ-[Zn]-NQ.The MO energies are in Hartree.

# Table S9.Computed positions of the electronic transitions, oscillator strength (f), andmajor contributions of the NQ-[Zn]-NQ.

| Wavelength (nm) | Osc. Strength | Major contribs |
| --- | --- | --- |
| 793.5 | 0.7098 | HOMO→LUMO (97%) |
| 768.0 | 0 | HOMO→L+1 (98%) |
| 638.1 | 0.8064 | HOMO→L+2 (90%) |
| 610.5 | 0.0053 | H-1→LUMO (32%), H-1→L+2 (18%), HOMO→L+3 (50%) |
| 571.5 | 0 | H-1→L+1 (99%) |
| 570.8 | 0 | H-3→L+1 (-11%), H-2→LUMO (83%) |
| 565.9 | 0.033 | H-1→LUMO (67%), H-1→L+2 (-15%), HOMO→L+3 (-17%) |
| 561.3 | 0.0977 | H-3→LUMO (-14%), H-2→L+1 (78%) |
| 484.7 | 0.1518 | H-3→LUMO (71%), H-2→L+1 (17%) |
| 478.6 | 0.0001 | H-3→L+1 (51%), H-2→L+2 (36%) |
| 471.7 | 0 | H-3→L+1 (-29%), H-2→L+2 (63%) |
| 456.0 | 0.826 | H-1→L+2 (55%), HOMO→L+3 (-27%) |
| 453.1 | 0.0041 | H-4→LUMO (93%) |
| 452.6 | 1.1733 | H-3→L+2 (31%), H-1→L+3 (46%) |
| 444.5 | 0.1196 | H-5→LUMO (73%) |
| 443.5 | 0.0044 | H-14→LUMO (11%), H-13→LUMO (-30%), H-13→L+1 (41%) |
| 443.3 | 0.004 | H-4→L+1 (80%) |
| 443.2 | 0.0038 | H-14→LUMO (27%), H-14→L+1 (32%), H-4→L+1 (-18%) |
| 436.5 | 0 | H-5→L+1 (91%) |
| 430.8 | 0 | H-2→L+3 (96%) |
| 415.9 | 0.1747 | H-5→L+2 (34%), H-3→L+2 (39%), H-1→L+3 (-13%) |
| 415.8 | 0.0011 | H-4→L+2 (92%) |
| 408.0 | 0.0005 | H-8→LUMO (11%), H-6→LUMO (36%) |
| 407.4 | 0.0022 | H-27→L+1 (-10%), H-8→L+1 (-10%), H-7→LUMO (29%) |
| 404.8 | 0.0001 | H-8→LUMO (-11%), H-6→LUMO (55%) |
| 398.9 | 0.103 | H-7→LUMO (10%), H-5→L+2 (36%) |
| 397.9 | 0.034 | H-7→LUMO (-17%), H-6→L+1 (30%) |
| 397.2 | 0.0022 | H-7→LUMO (22%), H-6→L+1 (63%) |
| 393.3 | 0.0445 | H-9→LUMO (25%), H-9→L+2 (41%), H-3→L+3 (18%) |
| 392.2 | 0 | H-7→L+1 (-28%), H-4→L+3 (-18%), HOMO→L+4 (42%) |
| 391.3 | 0.0005 | H-7→L+1 (49%), H-4→L+3 (-26%), HOMO→L+4 (13%) |
| 389.3 | 0.4088 | H-5→L+2 (13%), H-3→L+3 (35%) |
| 388.5 | 0.0006 | H-4→L+3 (52%), HOMO→L+4 (35%) |
| 384.7 | 0.0002 | H-27→LUMO (-10%), H-8→LUMO (53%) |
| 380.3 | 0.0157 | H-27→L+1 (-10%), H-8→L+1 (63%) |
| 376.4 | 0.0009 | H-6→L+2 (85%) |
| 373.5 | 0.1025 | H-10→LUMO (39%), H-10→L+2 (21%), H-5→L+3 (25%) |
| 371.4 | 0.0859 | H-7→L+2 (48%), H-5→L+3 (14%) |
| 370.7 | 0.0158 | H-11→LUMO (51%), H-11→L+2 (23%) |
| 367.0 | 0.2066 | H-10→LUMO (25%), H-7→L+2 (31%), H-5→L+3 (-20%) |
| 364.0 | 0.0001 | H-9→L+1 (67%), H-8→L+2 (15%) |
| 362.7 | 0.0003 | H-12→LUMO (14%), H-9→L+1 (-32%), H-8→L+2 (35%) |
| 358.7 | 0.0248 | H-9→L+3 (90%) |
| 358.7 | 0.0042 | H-9→LUMO (59%), H-9→L+2 (-37%) |
| 357.6 | 0.0001 | H-10→L+1 (88%) |
| 355.7 | 0.0078 | H-11→L+1 (88%) |
| 354.6 | 0.0012 | H-6→L+3 (94%) |
| 351.3 | 0.0177 | H-12→L+1 (-27%), HOMO→L+5 (33%) |
| 350.0 | 0.0002 | H-12→LUMO (42%), H-8→L+2 (-29%) |
| 349.1 | 0.052 | H-12→L+1 (-13%), H-10→L+2 (-12%), H-7→L+3 (27%), HOMO→L+5 (-18%) |
| 347.5 | 0.0059 | H-12→L+1 (10%), H-10→LUMO (12%), H-10→L+2 (-23%), HOMO→L+5 (32%) |
| 347.3 | 0.0001 | H-11→LUMO (-35%), H-11→L+2 (49%) |
| 346.5 | 0 | HOMO→L+6 (80%) |
| 345.1 | 0.0461 | H-10→L+2 (26%), H-7→L+3 (44%) |
| 343.6 | 0.0297 | H-21→LUMO (17%), H-18→LUMO (-26%), H-17→L+1 (28%) |
| 343.4 | 0.0008 | H-21→L+1 (11%), H-18→L+1 (-25%), H-17→LUMO (34%) |
| 341.7 | 0.0015 | H-21→L+1 (-21%), H-19→LUMO (24%), H-18→L+1 (-11%) |
| 341.4 | 0.1484 | H-21→LUMO (20%), H-19→L+1 (-17%), H-18→LUMO (15%) |
| 335.7 | 0.0001 | H-1→L+4 (93%) |
| 335.6 | 0.0562 | H-16→L+2 (-10%), H-15→LUMO (35%), H-10→L+3 (23%) |
| 334.2 | 0.0001 | H-15→L+1 (15%), H-12→L+2 (14%), H-8→L+3 (15%) |
| 334.0 | 0.0001 | H-8→L+3 (62%) |
| 332.4 | 0.0379 | H-16→LUMO (17%), H-15→LUMO (15%), H-12→L+1 (33%) |
| 328.0 | 0.0002 | H-15→L+1 (13%), H-12→LUMO (10%), H-12→L+2 (-27%), H-11→L+3 (33%) |
| 327.9 | 0.0323 | H-16→LUMO (24%), H-15→LUMO (-10%), H-10→L+3 (52%) |
| 324.3 | 0.0661 | HOMO→L+7 (71%) |
| 323.7 | 0 | H-16→L+1 (-32%), H-15→L+1 (34%), H-11→L+3 (-23%) |
| 321.5 | 0 | H-16→L+1 (-19%), H-12→L+2 (32%), H-11→L+3 (32%) |
| 319.8 | 0.0014 | H-13→LUMO (43%), H-13→L+1 (38%) |
| 319.5 | 0.0021 | H-14→LUMO (-40%), H-14→L+1 (41%) |
| 318.2 | 0.2709 | H-2→L+4 (76%) |
| 315.4 | 0.0905 | H-24→LUMO (-16%), H-23→L+1 (18%), H-16→L+2 (12%), H-15→LUMO (16%) |
| 314.8 | 0.0002 | H-23→LUMO (-12%), H-16→L+1 (-11%), HOMO→L+8 (43%) |
| 314.5 | 0.007 | H-25→LUMO (38%), H-25→L+2 (23%) |
| 314.1 | 0.0017 | H-26→LUMO (33%), H-26→L+2 (19%), HOMO→L+8 (17%) |
| 314.0 | 0.0003 | H-26→LUMO (-16%), H-26→L+2 (-10%), H-23→LUMO (12%), HOMO→L+8 (22%) |
| 312.8 | 0.0014 | H-16→LUMO (-16%), H-16→L+2 (23%), H-15→L+2 (-21%) |
| 310.7 | 0.0001 | H-20→LUMO (60%), H-19→LUMO (17%) |
| 309.4 | 0.0079 | H-18→LUMO (31%), H-17→L+1 (30%) |
| 309.0 | 0.0006 | H-22→LUMO (47%), H-18→L+1 (17%), H-17→LUMO (14%) |
| 308.9 | 0.0007 | H-22→LUMO (32%), H-18→L+1 (-26%), H-17→LUMO (-18%) |
| 306.7 | 0 | H-21→L+1 (-11%), H-20→L+1 (65%), H-19→L+1 (17%) |
| 306.3 | 0.0001 | H-12→L+3 (79%), H-8→L+3 (-10%) |
| 305.1 | 0.0006 | H-22→L+1 (85%) |
| 305.0 | 0.0044 | H-16→L+2 (17%), H-15→L+2 (19%), H-13→L+2 (18%) |
| 302.6 | 0.0001 | H-21→L+1 (17%), H-19→LUMO (33%), H-19→L+1 (20%) |
| 302.3 | 0.0005 | H-21→LUMO (33%), H-21→L+1 (-10%), H-20→L+1 (-13%), H-19→L+1 (19%) |
| 302.0 | 0 | H-21→L+1 (14%), H-14→L+2 (29%) |
| 301.9 | 0.0026 | H-1→L+5 (82%) |
| 300.7 | 0.0011 | H-26→L+1 (34%), H-25→L+2 (-26%), H-13→L+2 (13%) |
| 300.6 | 0.0008 | H-26→L+2 (-36%), H-25→L+1 (50%) |
| 300.3 | 0.0011 | H-15→L+2 (-11%), H-13→L+2 (24%), H-3→L+4 (12%), H-2→L+5 (-18%) |
| 300.2 | 0.0011 | H-14→L+2 (14%), H-3→L+4 (-18%), H-2→L+5 (23%) |
| 298.4 | 0.1178 | H-16→L+3 (46%), H-15→L+3 (-35%), H-1→L+5 (-12%) |
| 298.4 | 0.0024 | H-17→L+2 (-24%), H-14→L+2 (31%), HOMO→L+10 (23%) |
| 298.1 | 0.0012 | H-1→L+6 (93%) |
| 297.7 | 0.0506 | H-18→L+2 (27%), H-13→L+2 (15%), HOMO→L+9 (29%) |
| 293.9 | 0.1712 | H-3→L+5 (-10%), H-2→L+6 (70%) |
| 293.1 | 0.0002 | H-3→L+4 (49%), H-2→L+5 (23%) |


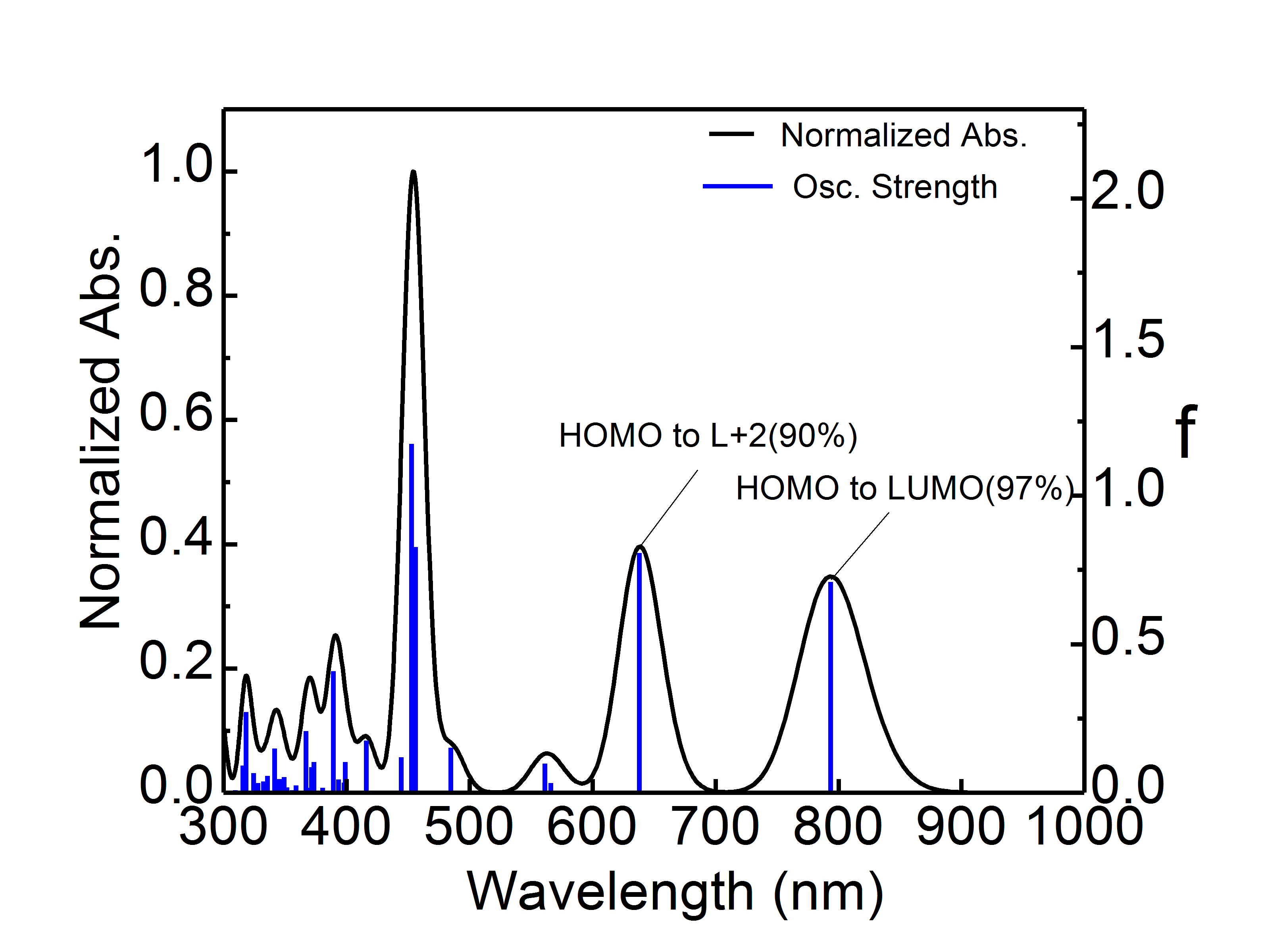


# Figure S20.Bar graph (blue) reporting the position of the electronic transitions vs the oscillator strength (f) for NQ-[Zn]-NQ.The black line represents a spectrum where 1000 cm-1 is applied to each transition.

# Table S10.Atomic contributions for the frontier MO for NQ-[Zn]-NQ.

|  | H-4 | H-3 | H-2 | H-1 | H | L | L+1 | L+2 | L+3 | L+4 |
| --- | --- | --- | --- | --- | --- | --- | --- | --- | --- | --- |
| Ethyne-porphyrins | 1.00 | 0.39 | 0.19 | ~1.00 | 0.72 | 0.07 | 0.01 | 0.80 | ~1.00 | 0.52 |
| 2*naphtoquinone | 0.00 | 0.61 | 0.81 | ~0.00 | 0.28 | 0.93 | 0.99 | 0.20 | ~0.00 | 0.48 |

**NQ-[S]-[Zn]-[S]-NQ**

# Figure S21.Structure of NQ-[S]-[Zn]-[S]-NQ.


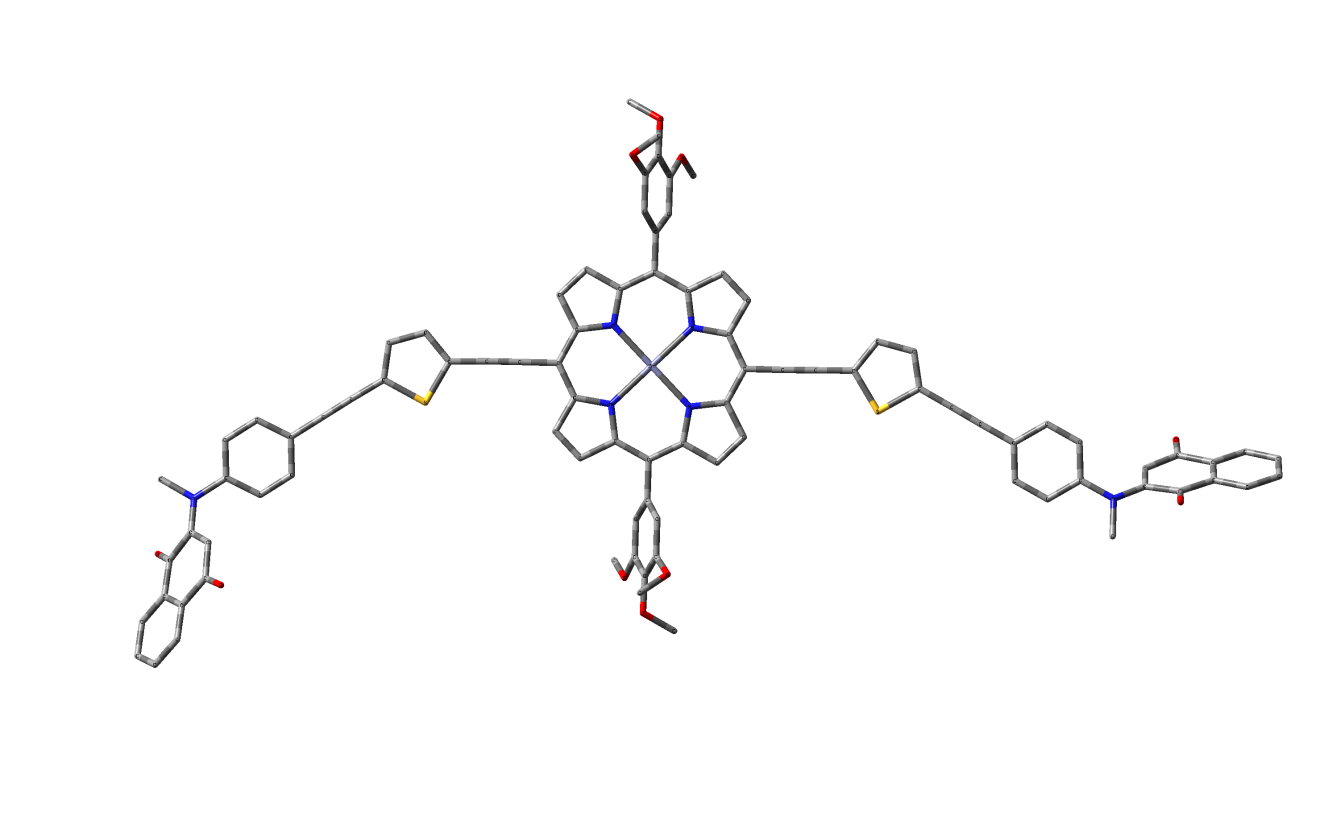


# Figure S22.Image of the optimized structure ofNQ-[S]-[Zn]-[S]-NQ.


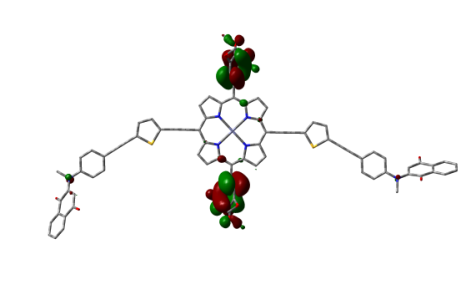


H-4 (-0.21540)


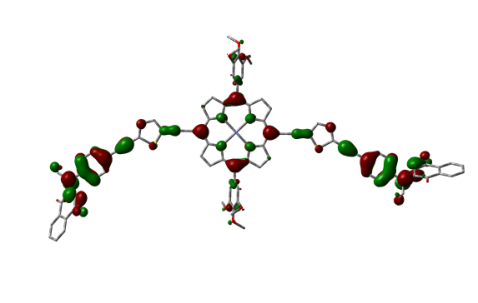


H-3 (-0.20079)


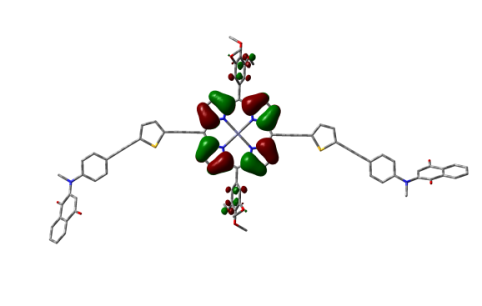


H-2 (-0.19737)


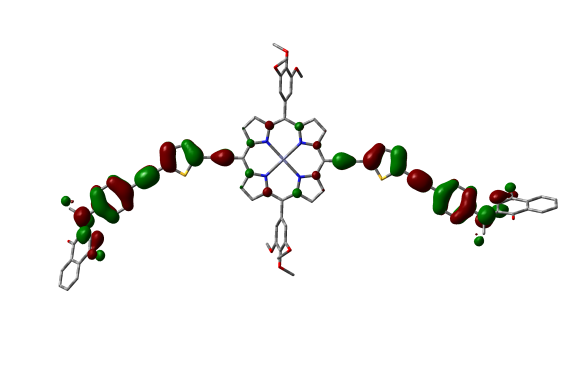


H-1 (-0.19110)


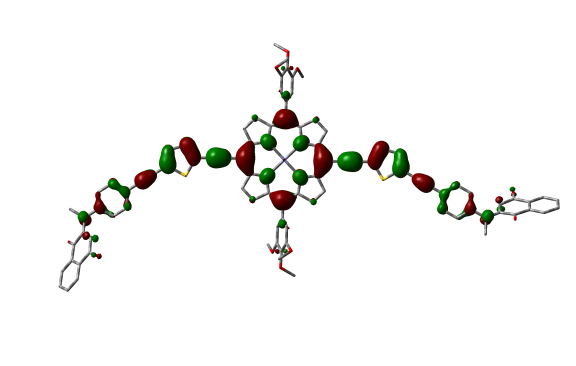


HOMO (-0.17450)


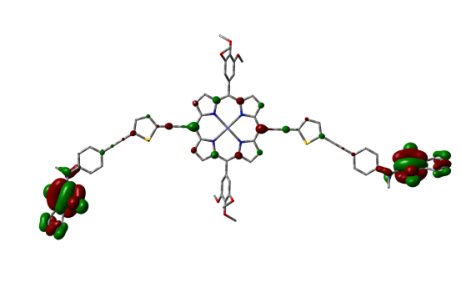


LUMO (-0.10696)


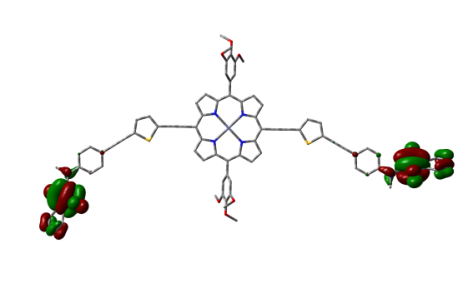


L+1 (-0.10610)


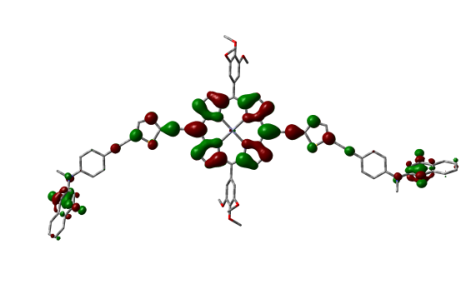


L+2 (-0.10103)


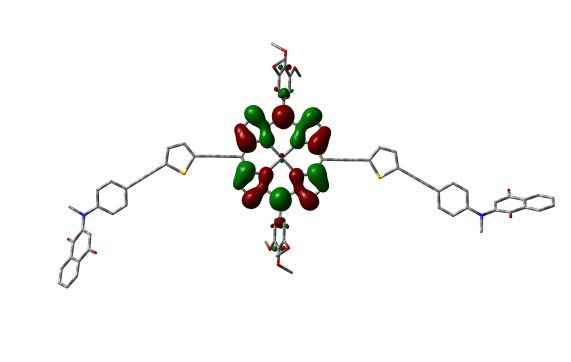


L+3 (-0.08409)


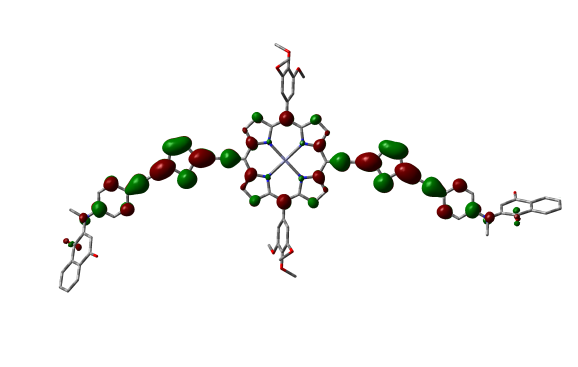


L+4 (-0.06708)

#

# Figure S23. Representations of the frontier MOs ofNQ-[S]-[Zn]-[S]-NQ.The MO energies are in Hartree.

# Table S11.Computed positions of the electronic transitions, oscillator strength (f), and major contributions of the NQ-[S]-[Zn]-[S]-NQ.

| Wavelength (nm) | Osc. Strength | Major contribs |
| --- | --- | --- |
| 778.1 | 1.7224 | HOMO→LUMO (86%) |
| 746.6 | 0.0671 | HOMO→L+1 (93%) |
| 702.6 | 0.8658 | HOMO→L+2 (89%) |
| 621.6 | 0.0911 | H-3→L+1 (13%), H-1→LUMO (77%) |
| 620.6 | 0.0049 | H-2→LUMO (-10%), H-2→L+2 (26%), HOMO→L+3 (62%) |
| 612.7 | 0.0927 | H-3→LUMO (15%), H-1→L+1 (73%) |
| 562.6 | 0.0225 | H-1→L+2 (93%) |
| 534.0 | 0.0437 | H-3→LUMO (26%), H-2→LUMO (45%), H-2→L+1 (10%) |
| 533.2 | 0.0574 | H-3→LUMO (39%), H-2→LUMO (-14%), H-2→L+1 (-30%) |
| 530.9 | 0.023 | H-2→LUMO (-28%), H-2→L+1 (60%) |
| 524.2 | 0.0088 | H-3→L+1 (73%), H-1→LUMO (-16%) |
| 508.3 | 0.2583 | H-3→L+2 (73%), H-2→L+3 (-12%) |
| 487.4 | 0.595 | H-2→L+2 (56%), HOMO→L+3 (-31%) |
| 472.5 | 0.0722 | H-1→L+3 (-41%), HOMO→L+4 (55%) |
| 472.4 | 0.0585 | H-1→L+3 (55%), HOMO→L+4 (41%) |
| 461.8 | 0.0008 | H-4→LUMO (52%), H-4→L+2 (-43%) |
| 455.5 | 0.0803 | H-5→LUMO (46%), H-5→L+2 (-29%) |
| 451.6 | 1.6608 | H-3→L+2 (12%), H-2→L+3 (42%), HOMO→L+5 (-24%) |
| 450.4 | 0.0478 | H-6→LUMO (51%), H-5→L+1 (11%) |
| 447.1 | 0.0799 | H-6→L+1 (26%), H-5→L+2 (18%), H-4→L+1 (-10%) |
| 445.4 | 0.0018 | H-15→LUMO (36%), H-15→L+1 (37%) |
| 444.9 | 0.0097 | H-14→LUMO (-26%), H-14→L+1 (40%) |
| 437.6 | 0.0023 | H-6→L+1 (15%), H-4→L+1 (66%) |
| 434.1 | 0.002 | H-4→LUMO (39%), H-4→L+2 (43%) |
| 431.0 | 0.0008 | H-5→L+1 (75%) |
| 426.8 | 0.0083 | H-6→L+1 (-10%), H-5→LUMO (42%), H-5→L+2 (37%) |
| 420.2 | 0.0632 | H-6→L+2 (71%) |
| 419.5 | 0.4276 | H-6→L+2 (-10%), H-3→L+3 (65%) |
| 413.2 | 0.0043 | H-7→LUMO (34%), H-7→L+2 (-33%), HOMO→L+5 (17%) |
| 411.1 | 0.0018 | H-8→LUMO (-39%), H-8→L+2 (45%) |
| 406.4 | 0.0161 | H-9→L+2 (12%), H-7→LUMO (-15%), HOMO→L+5 (30%) |
| 404.1 | 0.0089 | H-10→LUMO (-20%), H-10→L+2 (64%) |
| 402.4 | 0.0074 | H-29→LUMO (-12%), H-7→L+1 (12%) |
| 401.8 | 0.0031 | H-4→L+3 (85%) |
| 401.7 | 0.004 | H-29→L+1 (15%) |
| 397.5 | 0.3545 | H-5→L+3 (25%), H-1→L+4 (54%) |
| 395.1 | 0.0145 | H-7→L+1 (54%) |
| 393.1 | 0.0213 | H-5→L+3 (38%), H-1→L+4 (-10%) |
| 390.8 | 0.0113 | H-8→L+1 (38%), H-7→L+1 (-11%), H-7→L+2 (14%) |
| 390.2 | 0.0046 | H-8→L+1 (51%), H-7→LUMO (-15%), H-7→L+2 (-19%) |
| 387.6 | 0.0052 | H-8→LUMO (43%), H-8→L+2 (36%) |
| 386.5 | 0.0001 | H-2→L+4 (91%) |
| 376.9 | 0.0111 | H-9→LUMO (19%), H-9→L+1 (52%) |
| 375.8 | 0.0081 | H-9→LUMO (38%), H-9→L+1 (-21%) |
| 375.6 | 0.0005 | H-13→LUMO (-21%), H-13→L+2 (50%), H-6→L+3 (-16%) |
| 373.6 | 0.0116 | H-11→LUMO (-27%), H-11→L+2 (58%) |
| 369.7 | 0.1004 | H-12→LUMO (-14%), H-12→L+2 (30%), H-3→L+4 (39%) |
| 366.7 | 0.0017 | H-13→L+2 (10%), H-8→L+3 (14%), H-6→L+3 (60%) |
| 366.2 | 0.0762 | H-12→LUMO (14%), H-12→L+2 (-23%), H-3→L+4 (34%), H-1→L+5 (12%) |
| 365.6 | 0.0189 | H-9→L+2 (37%), H-7→L+3 (31%) |
| 362.7 | 0.0021 | H-8→L+3 (71%), H-6→L+3 (-11%) |
| 361.5 | 0.0015 | H-10→L+3 (94%) |
| 358.7 | 0.0571 | H-9→L+2 (-19%), H-7→L+3 (44%) |
| 358.1 | 0.0934 | H-3→L+4 (-12%), H-1→L+5 (62%), HOMO→L+6 (14%) |
| 354.4 | 0.0134 | H-1→L+5 (-12%), HOMO→L+6 (58%), HOMO→L+8 (-15%) |
| 351.0 | 0.0011 | H-10→LUMO (17%), H-10→L+1 (75%) |
| 349.6 | 0.0005 | H-10→LUMO (52%), H-10→L+1 (-21%), H-10→L+2 (18%) |
| 349.2 | 0.1517 | H-2→L+5 (91%) |
| 347.0 | 0.0065 | H-12→L+1 (34%) |
| 345.7 | 0.0056 | H-12→LUMO (20%), H-12→L+2 (12%) |
| 343.7 | 0.0005 | H-13→L+1 (-10%), H-11→LUMO (13%), H-11→L+1 (48%) |
| 342.4 | 0.002 | H-13→L+1 (30%), H-11→LUMO (-14%), H-11→L+1 (17%), HOMO→L+7 (-16%) |
| 342.2 | 0.0078 | H-12→L+1 (14%), HOMO→L+7 (33%) |
| 341.5 | 0.0176 | H-23→LUMO (25%), H-22→L+1 (17%), HOMO→L+8 (11%) |
| 340.9 | 0.023 | H-23→L+1 (-16%), H-21→L+1 (-11%), H-18→LUMO (22%), HOMO→L+7 (-12%) |
| 340.4 | 0.0528 | H-23→L+1 (11%), H-22→LUMO (14%), H-21→LUMO (-14%), H-18→L+1 (11%) |
| 340.1 | 0.0144 | H-21→LUMO (-10%), H-21→L+1 (12%), H-18→L+1 (12%), HOMO→L+8 (10%) |
| 340.0 | 0.0086 | H-13→LUMO (18%), H-13→L+1 (28%), H-11→LUMO (23%) |
| 339.5 | 0.0187 | H-23→LUMO (-10%), H-22→L+1 (-12%), H-12→LUMO (-10%), HOMO→L+8 (30%) |
| 338.9 | 0.0004 | H-13→LUMO (37%), H-13→L+1 (-20%), H-13→L+2 (16%), H-11→L+1 (11%) |
| 338.1 | 0.0179 | H-17→LUMO (-16%), H-17→L+2 (36%), H-11→L+3 (16%) |
| 334.5 | 0.0282 | H-12→L+1 (10%) |
| 333.9 | 0.0122 | H-16→LUMO (10%) |
| 332.3 | 0.2312 | H-3→L+5 (68%) |
| 330.2 | 0.0174 | H-4→L+4 (87%) |
| 327.2 | 0.0543 | H-11→L+3 (34%), H-9→L+3 (-12%), H-5→L+4 (31%) |
| 326.7 | 0.0334 | H-11→L+3 (-14%), H-5→L+4 (64%) |
| 325.8 | 0.187 | H-11→L+3 (19%), H-9→L+3 (61%) |
| 325.1 | 0.002 | H-12→L+3 (81%) |
| 324.8 | 0.0019 | H-13→L+3 (92%) |
| 319.6 | 0.0329 | H-6→L+4 (79%) |
| 319.0 | 0.0054 | H-16→LUMO (-12%), H-16→L+2 (18%), H-15→LUMO (-13%), H-15→L+1 (12%), H-14→LUMO (-13%) |
| 318.5 | 0.0003 | H-15→LUMO (27%), H-15→L+1 (-14%), H-14→LUMO (-26%), H-14→L+1 (-19%) |
| 317.9 | 0.0046 | H-16→LUMO (-15%), H-16→L+2 (17%), H-15→L+1 (-11%), H-14→LUMO (20%) |
| 315.0 | 0.0775 | H-1→L+6 (50%) |
| 314.0 | 0.0007 | H-19→LUMO (54%), H-19→L+2 (-25%) |
| 313.3 | 0.0303 | H-25→LUMO (-13%), H-24→LUMO (38%) |
| 312.9 | 0.0082 | H-25→LUMO (40%), H-25→L+1 (-12%), H-24→LUMO (11%) |
| 312.7 | 0.0005 | H-20→LUMO (38%), H-20→L+2 (-28%) |
| 312.4 | 0.0039 | H-17→L+1 (36%), H-15→L+2 (-11%) |
| 312.0 | 0.0059 | H-17→L+1 (30%), H-15→L+2 (39%) |
| 311.9 | 0.0015 | H-17→L+1 (10%), H-15→L+2 (-18%), H-14→L+1 (11%), H-14→L+2 (47%) |
| 311.4 | 0.0422 | H-17→L+1 (15%), H-16→L+1 (24%), H-1→L+6 (11%) |
| 311.2 | 0.009 | H-16→L+2 (20%), H-14→L+2 (-21%), H-1→L+7 (21%) |
| 310.2 | 0.0239 | H-17→LUMO (41%), H-17→L+2 (25%) |
| 308.7 | 0.0035 | HOMO→L+9 (52%) |
| 308.3 | 0.0103 | HOMO→L+10 (38%) |
| 308.0 | 0.0001 | H-36→L+2 (10%), H-32→L+2 (-11%), H-31→LUMO (-10%), H-31→L+2 (13%), HOMO→L+9 (-11%) |
| 307.7 | 0.0044 | H-31→LUMO (-13%), H-31→L+2 (18%) |

**
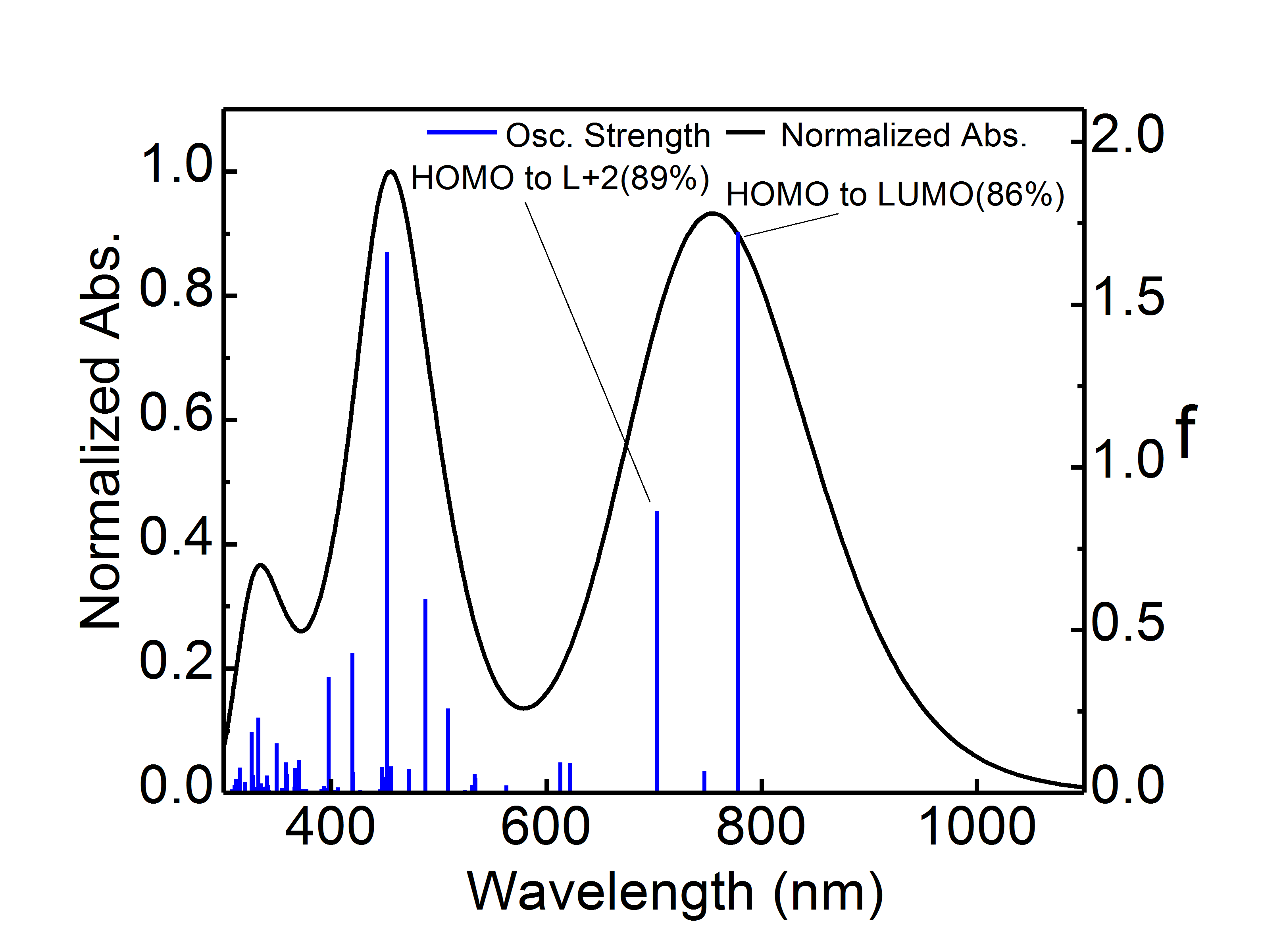
**

# Figure S24.Bar graph (blue) reporting the position of the electronic transitions vs the oscillator strength (f) for NQ-[S]-[Zn]-[S]-NQ.The black line represents a spectrum where 1000 cm-1 is applied to each transition.

# Table S12.Atomic contributions for the frontier MO for NQ-[S]-[Zn]-[S]-NQ.

|  | H-4 | H-3 | H-2 | H-1 | H | L | L+1 | L+2 | L+3 | L+4 |
| --- | --- | --- | --- | --- | --- | --- | --- | --- | --- | --- |
| 2*thiopene-porphyrins | 0.97 | 0.40 | ~1.00 | 0.47 | 0.87 | 0.16 | 0.02 | 0.81 | ~1.00 | 0.81 |
| 2*naphtoquinone | 0.03 | 0.60 | ~0.0 | 0.53 | 0.13 | 0.84 | 0.98 | 0.19 | ~0.0 | 0.19 |

**NQ-[B-Zn]-NQ**

|  |  |
| --- | --- |

**Figure S25.**Structure of **NQ-[B-Zn]-NQ**.


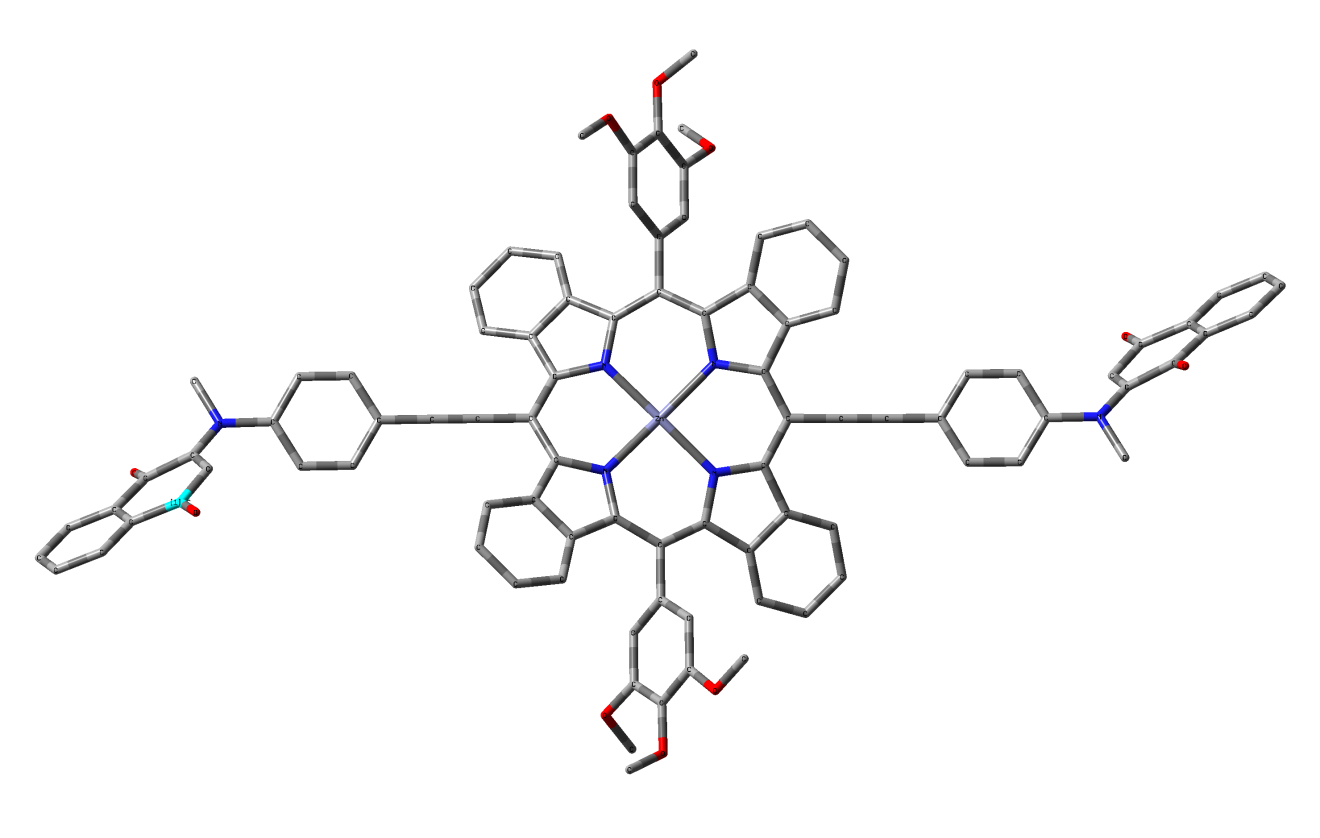


# Figure S26.Image of the optimized structure of NQ-[B-Zn]-NQ.


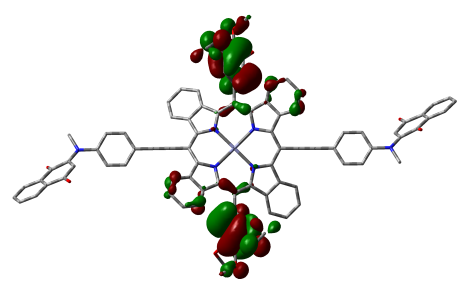


H-4 (-0.21413)


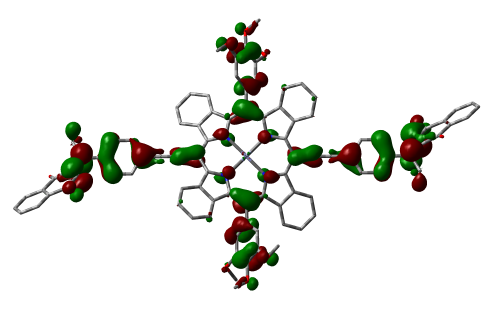


H-3 (-0.20874)


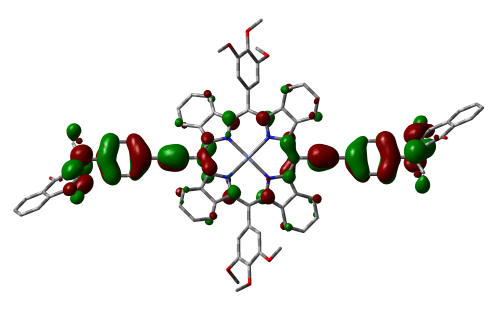


H-2 (-0.19662)


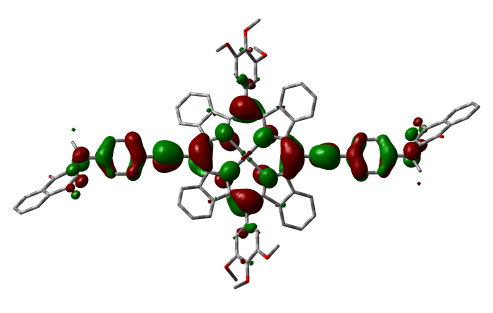


H-1 (-0.17524)


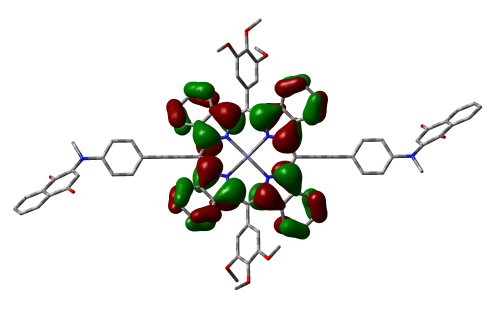


HOMO (-0.17222)


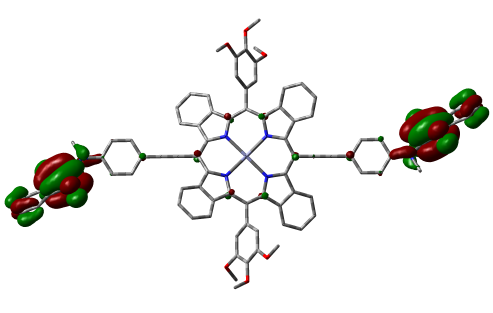


LUMO (-0.10608)


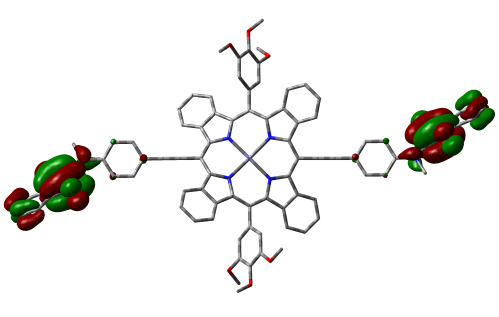


L+1 (-0.10519)


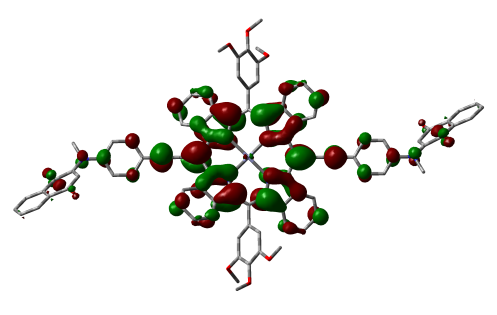


L+2 (-0.09055)


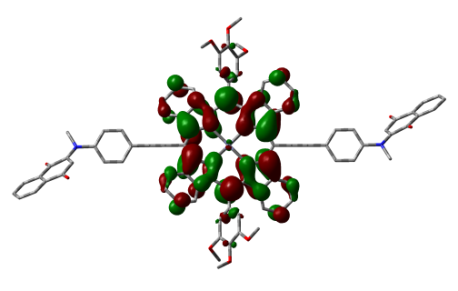


L+3 (-0.08010)


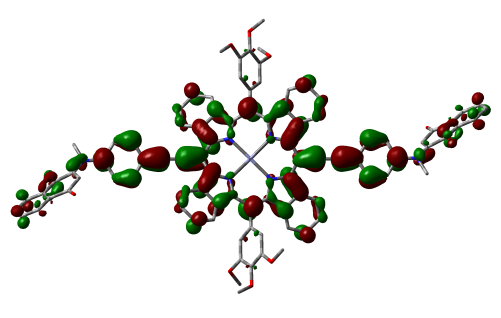


L+4 (-0.04596)

# Figure S27. Representations of the frontier MOs of NQ-[B-Zn]-NQ.The MO energies are inHartree.

# Table S13.Computed positions of the electronic transitions, oscillator strength (f), and major contributions of the NQ-[B-Zn]-NQ.

| Wavelength (nm) | Osc. Strength | Major contribs |
| --- | --- | --- |
| 786.9 | 0.0331 | HOMO→LUMO (97%) |
| 774.4 | 0.6035 | H-1→LUMO (90%) |
| 773.3 | 0.015 | HOMO→L+1 (97%) |
| 751.6 | 0.0004 | H-1→L+1 (96%) |
| 671.7 | 0.1108 | H-1→L+3 (24%), HOMO→L+2 (74%) |
| 655.1 | 0.0913 | H-1→L+2 (66%), HOMO→L+3 (-31%) |
| 583.9 | 0.0047 | H-3→L+1 (-10%), H-2→LUMO (83%) |
| 578.3 | 0.3697 | H-3→LUMO (-13%), H-2→L+1 (74%) |
| 526.7 | 2.0594 | H-1→L+2 (28%), HOMO→L+3 (55%) |
| 510.8 | 1.0643 | H-1→L+3 (74%), HOMO→L+2 (-25%) |
| 488.2 | 0.1133 | H-3→LUMO (69%), H-2→L+1 (11%) |
| 487.4 | 0.0076 | H-3→L+1 (42%), H-2→L+2 (-38%) |
| 481.7 | 0.0012 | H-3→L+1 (32%), H-2→L+2 (58%) |
| 456.1 | 0.0001 | H-4→LUMO (92%) |
| 450.3 | 0.0022 | H-5→LUMO (-45%), H-4→L+1 (46%) |
| 446.0 | 0.001 | H-5→LUMO (33%), H-4→L+1 (49%) |
| 445.5 | 0.0005 | H-17→LUMO (32%), H-16→LUMO (-13%), H-16→L+1 (-32%) |
| 445.2 | 0.0008 | H-17→LUMO (-13%), H-17→L+1 (32%), H-16→LUMO (-28%), H-16→L+1 (14%) |
| 442.7 | 0.0042 | H-2→L+3 (89%) |
| 440.6 | 0.0003 | H-5→L+1 (82%) |
| 426.1 | 0.0481 | H-3→L+2 (83%) |
| 420.2 | 0.0006 | H-6→LUMO (73%) |
| 417.2 | 0.0002 | H-4→L+2 (91%) |
| 416.8 | 0.0025 | H-8→L+1 (-14%), H-7→LUMO (14%), H-6→L+1 (41%) |
| 411.2 | 0 | H-8→LUMO (35%), H-7→L+1 (-13%), H-6→LUMO (15%) |
| 408.8 | 0.0001 | H-7→LUMO (-22%), H-6→L+1 (53%) |
| 406.1 | 0.0462 | HOMO→L+4 (88%) |
| 404.8 | 0.0407 | H-5→L+2 (77%) |
| 400.4 | 0.0012 | H-31→L+1 (-10%), H-7→LUMO (52%) |
| 397.7 | 0.0002 | H-1→L+4 (79%) |
| 396.2 | 0.0013 | H-7→L+1 (69%), H-1→L+4 (11%) |
| 396.0 | 0.1718 | H-5→L+3 (-14%), H-3→L+3 (81%) |
| 393.8 | 0.001 | H-31→LUMO (11%), H-8→LUMO (43%) |
| 391.0 | 0.0055 | H-31→L+1 (11%), H-8→L+1 (57%) |
| 389.8 | 0.0005 | H-4→L+3 (97%) |
| 386.4 | 0.0077 | H-9→LUMO (26%), H-6→L+2 (54%) |
| 384.5 | 0.0012 | H-9→LUMO (53%), H-6→L+2 (-35%) |
| 380.2 | 0.0038 | H-10→LUMO (42%), H-9→L+1 (-37%) |
| 377.9 | 0.0539 | H-9→L+1 (15%), H-5→L+3 (68%), H-3→L+3 (10%) |
| 373.2 | 0.0148 | H-7→L+2 (75%) |
| 372.9 | 0.002 | H-11→LUMO (49%), H-11→L+2 (-10%), H-8→L+2 (14%) |
| 372.6 | 0.0027 | H-10→LUMO (35%), H-9→L+1 (35%) |
| 368.2 | 0.0001 | H-10→L+1 (80%) |
| 366.8 | 0.0003 | H-12→LUMO (-32%), H-11→L+1 (46%) |
| 365.2 | 0.0003 | H-11→LUMO (-12%), H-9→L+2 (-18%), H-8→L+2 (38%), H-6→L+3 (-10%) |
| 361.8 | 0.0918 | H-15→LUMO (-36%), H-15→L+2 (53%) |
| 361.4 | 0.0052 | H-8→L+2 (11%), H-6→L+3 (69%) |
| 360.8 | 0.0026 | H-13→LUMO (-26%), H-9→L+2 (29%), H-8→L+2 (16%) |
| 359.2 | 0.0088 | H-12→LUMO (50%), H-11→L+1 (30%) |
| 357.9 | 0.0575 | HOMO→L+5 (67%), HOMO→L+7 (-20%) |
| 357.3 | 0.0041 | H-13→LUMO (50%), H-10→L+1 (10%), H-9→L+2 (25%) |
| 356.7 | 0.0006 | H-12→L+1 (70%) |
| 354.9 | 0.0064 | H-13→L+1 (44%), H-10→L+2 (-35%) |
| 354.0 | 0.2165 | H-14→LUMO (24%), H-1→L+5 (39%) |
| 352.5 | 0.0002 | HOMO→L+8 (70%) |
| 352.0 | 0.0065 | H-14→LUMO (13%), H-13→L+1 (35%), H-10→L+2 (22%), H-7→L+3 (-11%) |
| 351.7 | 0.0257 | H-14→LUMO (-21%), H-7→L+3 (-25%), H-1→L+5 (29%) |
| 350.8 | 0.0014 | H-14→LUMO (-12%), H-10→L+2 (15%), H-7→L+3 (50%) |
| 349.4 | 0.0019 | H-1→L+8 (24%), HOMO→L+6 (50%), HOMO→L+9 (11%) |
| 347.9 | 0.0001 | H-14→L+1 (32%), H-11→L+2 (-24%), H-1→L+6 (-12%) |
| 346.3 | 0.0002 | H-14→L+1 (42%), H-1→L+6 (21%) |
| 345.3 | 0.0011 | H-8→L+3 (-27%), H-1→L+8 (-16%), HOMO→L+6 (27%) |
| 343.9 | 0.0495 | H-20→L+1 (-12%), H-19→LUMO (34%), H-18→L+1 (-22%) |
| 343.6 | 0.0031 | H-14→L+1 (10%), H-11→L+2 (20%) |
| 342.4 | 0.0012 | H-15→L+1 (20%), H-1→L+6 (32%) |
| 341.7 | 0.0026 | H-20→LUMO (-10%), H-15→L+1 (53%) |
| 341.2 | 0.0603 | H-25→LUMO (17%), H-24→LUMO (-14%), H-24→L+1 (25%) |
| 340.8 | 0.0077 | HOMO→L+5 (23%), HOMO→L+7 (70%) |
| 340.6 | 0.0001 | H-25→L+1 (14%), H-24→LUMO (23%), H-24→L+1 (-15%), H-15→L+1 (15%) |
| 339.8 | 0.1458 | H-21→LUMO (20%), H-20→L+1 (11%), H-1→L+7 (11%) |
| 339.0 | 0.0034 | H-13→L+2 (20%), H-8→L+3 (39%), H-1→L+8 (-17%) |
| 338.6 | 0.0026 | H-14→L+2 (10%), H-12→L+2 (52%) |
| 337.9 | 0.0031 | H-13→L+2 (10%), H-1→L+6 (10%), HOMO→L+9 (11%) |
| 337.9 | 0.0017 | H-13→L+2 (37%), HOMO→L+9 (13%) |
| 336.9 | 0.0139 | H-15→LUMO (50%), H-15→L+2 (32%) |
| 336.3 | 0.028 | H-12→L+2 (-13%), H-10→L+3 (26%), H-1→L+7 (10%) |
| 335.2 | 0.0472 | H-13→L+2 (-12%), H-1→L+8 (-23%), HOMO→L+9 (42%), HOMO→L+10 (-11%) |
| 334.1 | 0.0102 | H-14→L+2 (-21%), H-1→L+7 (38%) |
| 333.5 | 0.0359 | H-15→L+3 (71%), H-9→L+3 (17%) |
| 333.1 | 0.0102 | H-15→L+3 (-23%), H-9→L+3 (51%) |
| 331.0 | 0.2514 | H-2→L+4 (70%) |
| 329.0 | 0.0001 | H-1→L+9 (73%) |
| 327.9 | 0.007 | H-14→L+2 (-32%), H-10→L+3 (34%), H-2→L+4 (11%) |
| 324.1 | 0.0429 | H-10→L+3 (15%), HOMO→L+11 (73%) |
| 322.5 | 0.0497 | H-11→L+3 (80%) |
| 321.0 | 0.0005 | H-26→LUMO (27%), H-22→LUMO (-13%), H-20→LUMO (-11%) |
| 319.6 | 0 | H-17→LUMO (34%), H-17→L+1 (17%), H-16→LUMO (18%), H-16→L+1 (28%) |
| 319.4 | 0 | H-17→LUMO (-16%), H-17→L+1 (30%), H-16→LUMO (30%), H-16→L+1 (-21%) |
| 318.2 | 0.0021 | H-14→L+3 (16%), H-1→L+11 (74%) |
| 317.4 | 0.0047 | H-27→LUMO (21%), H-26→L+1 (16%), H-18→L+1 (11%) |
| 316.6 | 0.0298 | H-14→L+3 (11%), H-12→L+3 (73%), HOMO→L+12 (-10%) |
| 315.5 | 0.0333 | HOMO→L+9 (15%), HOMO→L+10 (73%) |
| 314.4 | 0 | H-18→LUMO (14%), H-13→L+3 (51%) |
| 314.2 | 0.1218 | H-14→L+3 (16%), HOMO→L+12 (62%) |
| 313.1 | 0.0004 | H-19→L+1 (13%), H-18→LUMO (30%), H-13→L+3 (-22%) |
| 311.4 | 0.0142 | H-19→LUMO (21%), H-18→L+1 (42%) |
| 310.9 | 0.0222 | H-14→L+3 (50%), H-12→L+3 (-17%), H-1→L+11 (-18%) |
| 310.8 | 0.0394 | H-20→L+1 (12%) |
| 310.5 | 0.0011 | H-1→L+10 (60%) |

**
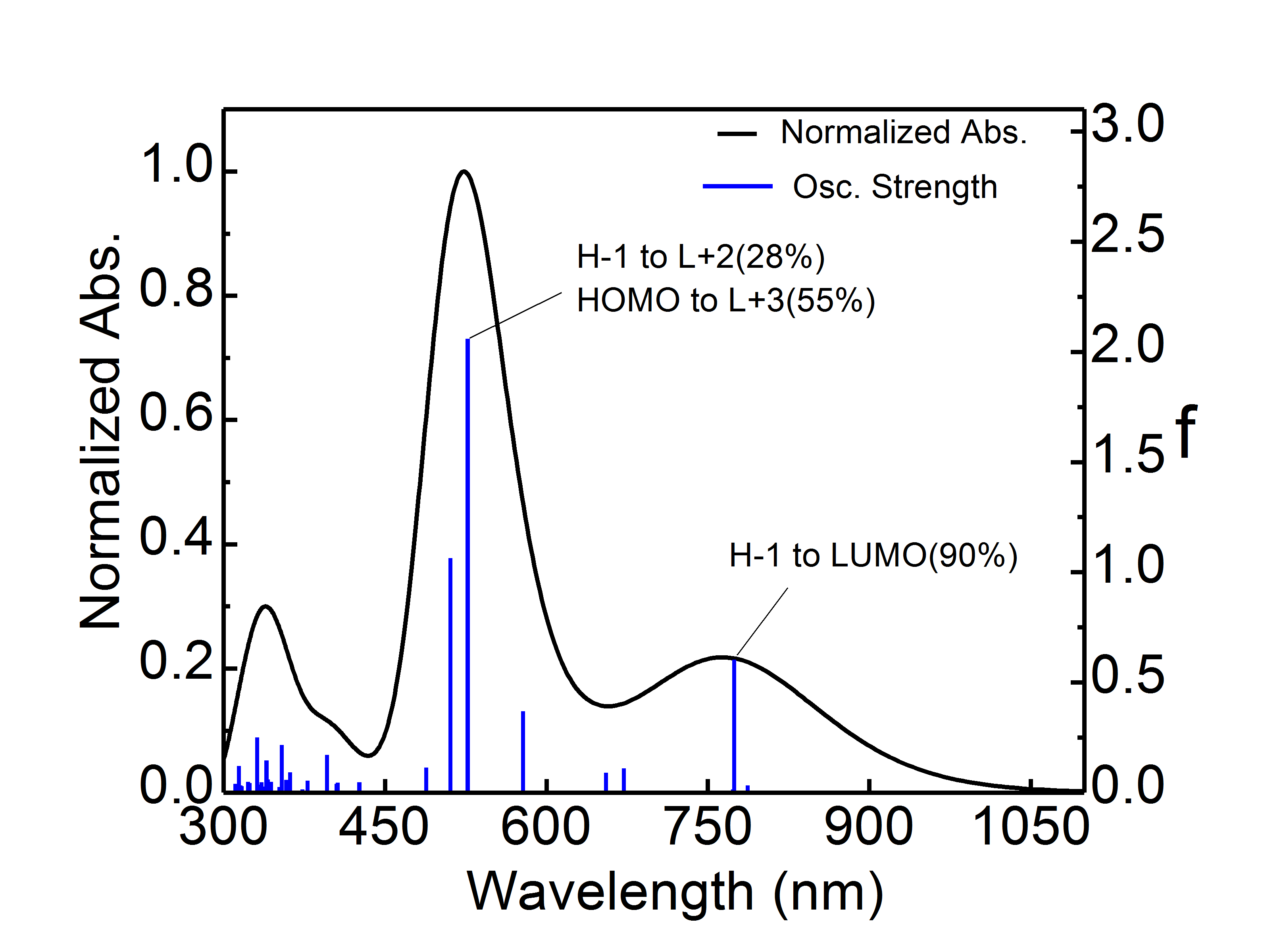
**

# Figure S28.Bar graph (blue) reporting the position of the electronic transitions vs the oscillator strength (f) for NQ-[B-Zn]-NQ.The black line represents a spectrum where 1000 cm-1 is applied to each transition.

# Table S14.Atomic contributions for the frontier MO for NQ-[B-Zn]-NQ.

|  | H-4 | H-3 | H-2 | H-1 | H | L | L+1 | L+2 | L+3 | L+4 |
| --- | --- | --- | --- | --- | --- | --- | --- | --- | --- | --- |
| benzo-porphyrins | ~1.00 | 0.43 | 0.29 | 0.74 | 0.99 | 0.06 | 0.01 | 0.85 | 0.99 | 0.61 |
| 2*naphtoquinone | ~0.0 | 0.57 | 0.71 | 0.26 | 0.01 | 0.94 | 0.99 | 0.15 | 0.01 | 0.39 |

**NQ-[S]-[B-Zn]-[S]-NQ**

# Figure S29.Structure of NQ-[S]-[B-Zn]-[S]-NQ.


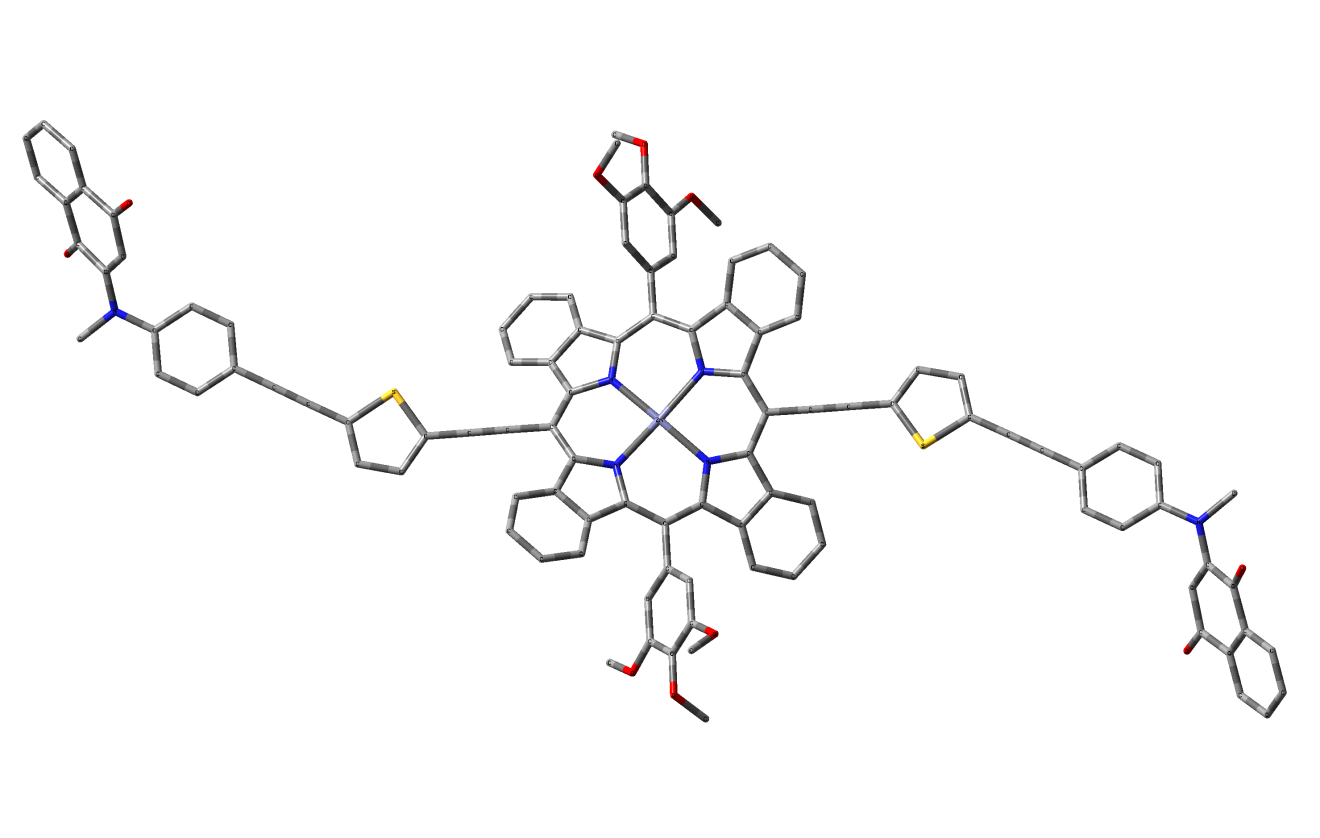


# Figure S30.Image of the optimized structure of NQ-[S]-[B-Zn]-[S]-NQ.


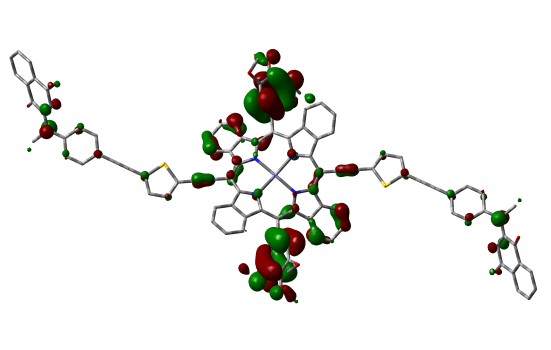


H-4 (-0.21401)


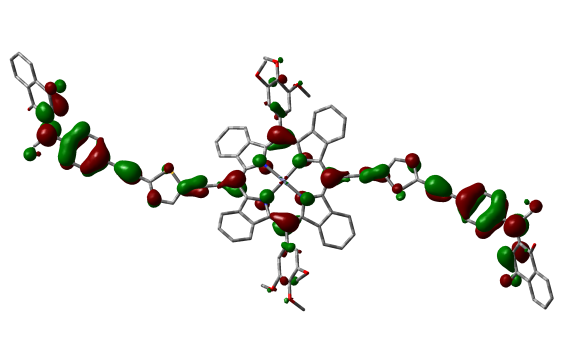


H-3 (-0.19968)


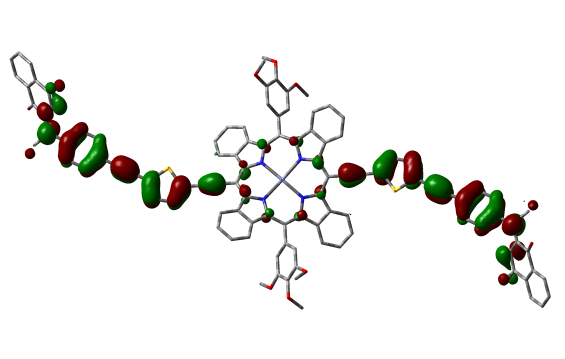


H-2 (-0.18995)


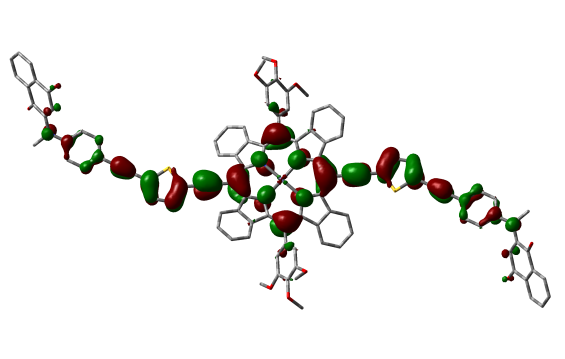


H-1 (-0.17539)


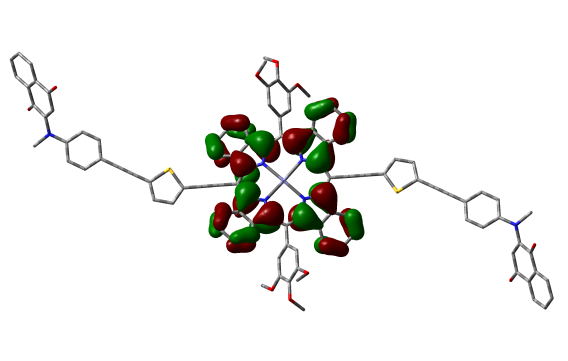


HOMO (-0.17348)


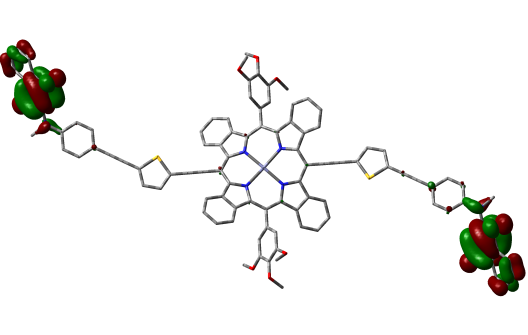


LUMO (-0.10621)


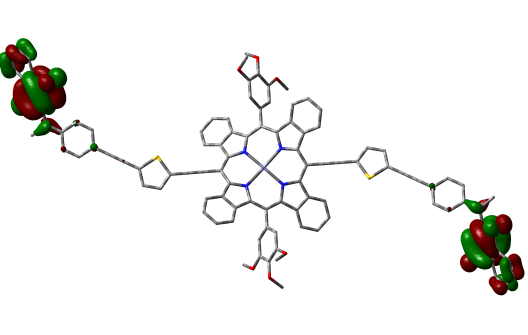


L+1 (-0.10582)


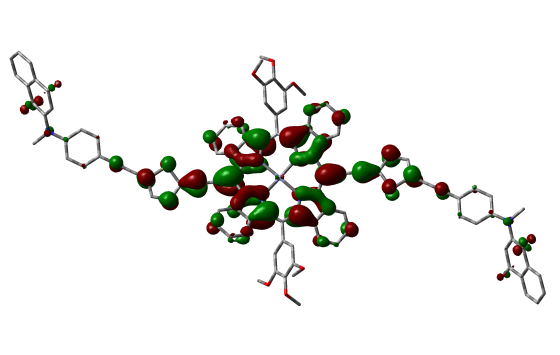


L+2 (-0.09694)


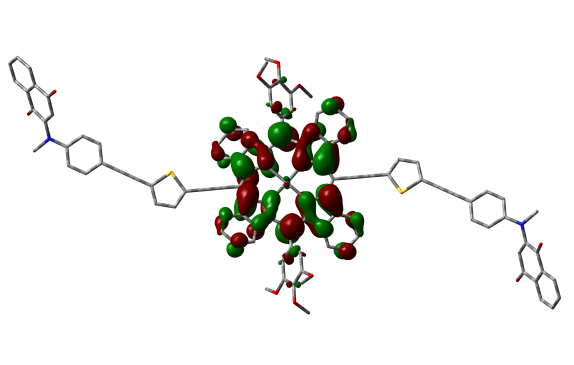


L+3 (-0.08130)


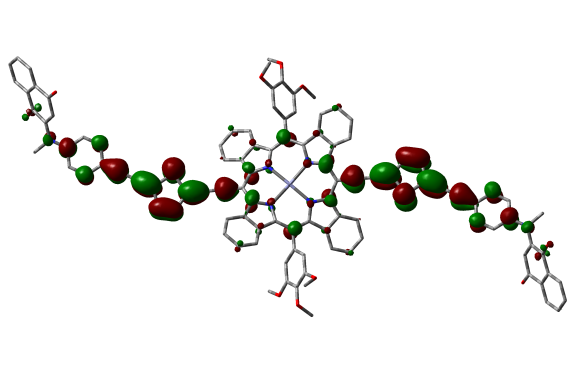


L+4 (-0.06764)

# Figure S31. Representations of the frontier MOs of NQ-[S]-[B-Zn]-[S]-NQ.The MO energies are in Hartree.

# Table S15.Computed positions of the electronic transitions,oscillator strength (f), and major contributions of the NQ-[S]-[B-Zn]-[S]-NQ.

| Wavelength (nm) | Osc. Strength | Major contribs |
| --- | --- | --- |
| 754.8 | 1.0425 | H-1→LUMO (76%) |
| 747.3 | 0.1286 | HOMO→LUMO (83%) |
| 736.8 | 0.0017 | H-1→L+1 (19%), HOMO→L+1 (79%) |
| 733.7 | 0.003 | H-1→L+1 (72%), HOMO→L+1 (-21%) |
| 700.9 | 0.125 | H-1→L+3 (14%), HOMO→LUMO (10%), HOMO→L+2 (74%) |
| 688.3 | 0.4467 | H-1→LUMO (11%), H-1→L+2 (73%), HOMO→L+3 (-14%) |
| 621.7 | 0.0032 | H-3→L+1 (14%), H-2→LUMO (74%) |
| 617.7 | 0.3036 | H-3→LUMO (16%), H-2→L+1 (70%) |
| 560.8 | 2.2122 | H-3→L+2 (-12%), H-1→L+2 (14%), HOMO→L+3 (61%) |
| 548.8 | 0.0016 | H-2→L+2 (94%) |
| 531.1 | 0.1683 | H-3→LUMO (70%), H-2→L+1 (-16%) |
| 530.0 | 0.0054 | H-3→L+1 (71%), H-2→LUMO (-17%) |
| 527.2 | 0.7148 | H-1→L+3 (79%), HOMO→L+2 (-17%) |
| 488.8 | 0.009 | HOMO→L+4 (97%) |
| 478.1 | 0.3699 | H-3→L+2 (78%) |
| 475.4 | 0.0028 | H-1→L+4 (96%) |
| 466.6 | 0.006 | H-2→L+3 (93%) |
| 460.7 | 0.0002 | H-6→LUMO (-27%), H-4→LUMO (41%) |
| 459.3 | 0.0158 | H-6→L+1 (-30%), H-5→LUMO (16%), H-4→L+1 (30%) |
| 450.1 | 0.0013 | H-6→LUMO (33%), H-4→LUMO (24%), H-4→L+2 (-31%) |
| 445.5 | 0.0029 | H-18→LUMO (-34%), H-18→L+1 (48%) |
| 445.2 | 0.0017 | H-19→LUMO (46%), H-19→L+1 (35%) |
| 444.6 | 0.0185 | H-5→LUMO (40%), H-5→L+2 (-31%) |
| 440.6 | 0.0035 | H-6→L+1 (37%), H-4→L+1 (58%) |
| 437.6 | 0.095 | H-3→L+3 (12%), HOMO→L+5 (83%) |
| 436.9 | 0 | H-6→LUMO (16%), H-5→L+1 (69%), H-4→L+2 (13%) |
| 433.9 | 0.0005 | H-5→L+1 (-20%), H-4→LUMO (29%), H-4→L+2 (43%) |
| 429.5 | 0.0183 | H-5→LUMO (34%), H-5→L+2 (57%) |
| 421.6 | 0.0018 | H-6→L+2 (69%) |
| 418.4 | 0.1202 | H-1→L+5 (75%) |
| 416.0 | 0.4988 | H-3→L+3 (77%), HOMO→L+5 (-12%) |
| 412.6 | 0.0116 | H-7→LUMO (49%), H-6→L+1 (10%) |
| 411.8 | 0.0014 | H-8→LUMO (39%), H-8→L+2 (-11%), H-7→L+1 (12%), H-6→L+2 (16%) |
| 408.2 | 0.0018 | H-8→LUMO (-12%), H-8→L+2 (26%), H-7→L+1 (26%) |
| 405.1 | 0.3639 | H-7→L+2 (17%), H-2→L+4 (54%) |
| 403.5 | 0.3962 | H-7→L+2 (-13%), H-2→L+4 (33%) |
| 401.1 | 0.0039 | H-8→L+1 (64%) |
| 399.1 | 0.0005 | H-7→L+1 (33%) |
| 396.3 | 0.0017 | H-8→LUMO (37%), H-8→L+2 (43%) |
| 394.4 | 0.034 | H-7→LUMO (21%), H-7→L+2 (46%) |
| 393.4 | 0.0021 | H-6→L+3 (12%), H-4→L+3 (84%) |
| 388.0 | 0.0061 | H-5→L+3 (90%) |
| 385.2 | 0.001 | H-10→LUMO (-25%), H-10→L+2 (54%) |
| 385.0 | 0.0196 | H-9→LUMO (61%) |
| 383.5 | 0.0003 | H-9→L+1 (65%) |
| 376.6 | 0.0032 | H-12→LUMO (-25%), H-12→L+2 (56%) |
| 375.9 | 0.0019 | H-11→L+2 (22%), H-3→L+4 (45%), H-2→L+5 (-12%) |
| 374.8 | 0.0043 | H-11→LUMO (-14%), H-11→L+2 (26%), H-3→L+4 (-15%), H-2→L+5 (17%) |
| 373.2 | 0.002 | H-9→L+2 (59%) |
| 372.7 | 0.0026 | H-6→L+3 (62%) |
| 369.5 | 0.0013 | H-10→L+1 (90%) |
| 367.3 | 0.0035 | H-10→LUMO (55%), H-10→L+2 (25%) |
| 366.2 | 0.001 | H-3→L+4 (29%), H-2→L+5 (58%) |
| 365.5 | 0.0271 | HOMO→L+6 (47%), HOMO→L+8 (-23%) |
| 363.5 | 0.0192 | H-17→L+2 (61%) |
| 363.1 | 0.0029 | H-11→L+1 (63%) |
| 362.2 | 0.0005 | H-12→L+1 (-27%), H-11→LUMO (29%), H-11→L+2 (13%) |
| 360.5 | 0.0011 | H-8→L+3 (59%) |
| 359.1 | 0.0179 | H-7→L+3 (76%) |
| 358.9 | 0.0015 | H-12→L+1 (45%), H-11→LUMO (12%) |
| 358.3 | 0.0012 | H-1→L+6 (42%), H-1→L+8 (-15%), HOMO→L+9 (-26%) |
| 357.1 | 0.0011 | H-12→LUMO (64%), H-12→L+2 (24%) |
| 355.9 | 0.0322 | H-13→LUMO (-26%), H-13→L+2 (34%), H-12→L+1 (-10%) |
| 353.2 | 0.0325 | H-16→LUMO (-17%), H-16→L+2 (24%), H-15→LUMO (-11%), H-14→L+1 (22%) |
| 352.7 | 0.0019 | H-14→LUMO (38%), H-11→LUMO (-12%), H-11→L+2 (-13%), HOMO→L+9 (-12%) |
| 352.3 | 0.067 | H-16→LUMO (-10%), H-16→L+2 (31%), H-15→LUMO (17%), H-14→L+1 (-15%), H-11→L+1 (10%) |
| 349.4 | 0.0003 | H-14→L+2 (11%), H-1→L+6 (14%), HOMO→L+9 (45%) |
| 347.3 | 0.0001 | H-13→L+1 (90%) |
| 346.6 | 0.2062 | H-15→LUMO (-14%), H-15→L+2 (29%), H-14→L+1 (-10%), H-3→L+5 (22%) |
| 344.9 | 0.0003 | H-13→LUMO (51%), H-13→L+2 (29%) |
| 343.2 | 0.1897 | H-15→LUMO (10%), H-14→L+1 (12%), H-3→L+5 (40%) |
| 343.0 | 0.0419 | H-15→L+1 (-15%), H-14→L+2 (15%), H-1→L+6 (-13%) |
| 342.2 | 0.0286 | H-1→L+9 (44%), HOMO→L+6 (-29%) |
| 341.4 | 0.0494 | H-25→L+1 (-11%), H-20→L+1 (10%), H-1→L+7 (27%) |
| 341.0 | 0.0029 | H-25→LUMO (15%), H-24→L+1 (18%), H-21→L+1 (13%), H-20→LUMO (-13%) |
| 340.6 | 0.0017 | HOMO→L+7 (81%) |
| 340.4 | 0.0003 | H-16→L+1 (69%), H-15→L+1 (-11%) |
| 340.1 | 0.0021 | H-21→LUMO (-15%), H-20→LUMO (11%), H-20→L+1 (18%), H-1→L+7 (-12%) |
| 339.9 | 0.0014 | H-21→LUMO (-10%), H-21→L+1 (26%), H-20→LUMO (-10%) |
| 339.7 | 0.0545 | H-25→L+1 (14%), H-24→LUMO (14%), H-1→L+7 (21%) |
| 339.4 | 0.0022 | H-16→L+1 (19%), H-15→L+1 (28%), H-1→L+8 (-14%) |
| 338.7 | 0.0106 | H-16→LUMO (43%), H-16→L+2 (11%), H-15→LUMO (-11%), H-15→L+2 (-10%) |
| 338.1 | 0.0371 | H-1→L+9 (26%), HOMO→L+8 (55%) |
| 337.5 | 0.0145 | H-16→LUMO (21%), H-16→L+2 (10%), H-15→LUMO (19%), H-15→L+2 (23%), H-14→L+1 (10%) |
| 336.9 | 0.0013 | H-17→L+3 (42%), H-9→L+3 (-41%) |
| 336.1 | 0.001 | H-14→L+2 (33%), H-1→L+8 (31%) |
| 334.6 | 0.0097 | H-4→L+4 (69%) |
| 334.4 | 0.0019 | H-10→L+3 (70%) |
| 332.5 | 0.0048 | H-17→L+1 (62%) |
| 332.2 | 0.0247 | H-17→LUMO (-21%), H-17→L+1 (12%), H-12→L+3 (21%) |
| 331.9 | 0.0001 | H-11→L+3 (26%), HOMO→L+11 (41%), HOMO→L+13 (-13%) |
| 331.5 | 0.0276 | H-12→L+3 (36%) |
| 331.2 | 0.0001 | H-5→L+4 (91%) |
| 330.6 | 0.0009 | H-29→L+1 (-19%), H-28→LUMO (14%), H-17→L+1 (24%) |
| 330.2 | 0.0388 | H-29→LUMO (-11%), H-17→LUMO (47%) |
| 329.6 | 0.027 | H-6→L+4 (71%) |
| 328.6 | 0.0155 | H-11→L+3 (46%), HOMO→L+11 (-23%) |
| 327.4 | 0.0494 | H-17→L+3 (31%), H-9→L+3 (24%), HOMO→L+10 (12%) |
| 326.1 | 0.0012 | HOMO→L+10 (52%), HOMO→L+14 (23%) |
| 323.5 | 0.0168 | H-12→L+3 (14%), HOMO→L+12 (71%) |

**
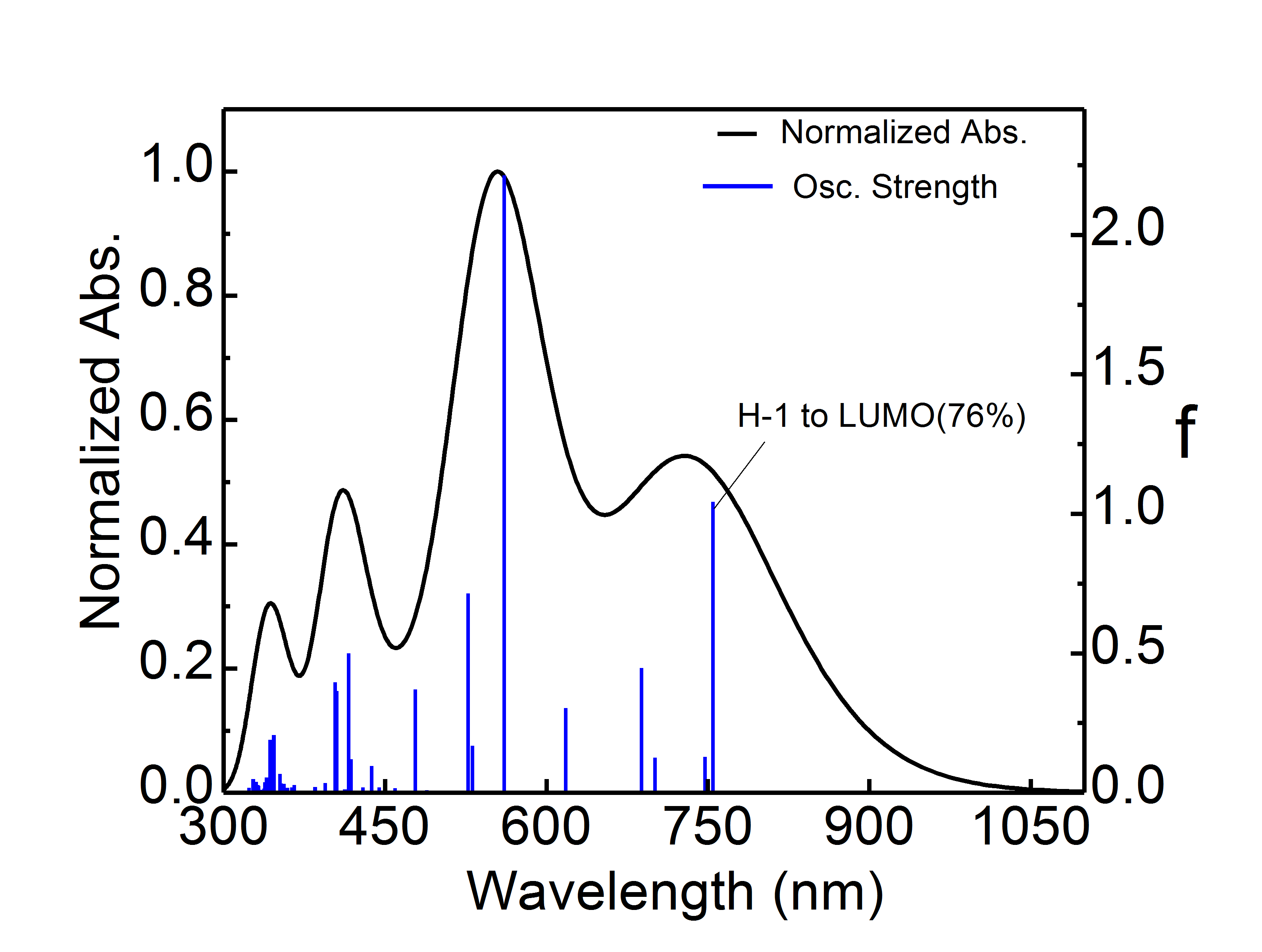
**

# Figure S32. Bar graph (blue) reporting the position of the electronic transitions vs the oscillator strength (f) for NQ-[S]-[B-Zn]-[S]-NQ.The black line represents a spectrum where 1000 cm-1 is applied to each transition.

# Table S16.Atomic contributions for the frontier MO for NQ-[S]-[B-Zn]-[S]-NQ.

|  | H-4 | H-3 | H-2 | H-1 | H | L | L+1 | L+2 | L+3 | L+4 |
| --- | --- | --- | --- | --- | --- | --- | --- | --- | --- | --- |
| 2*thiophene-  benzo-porphyrins | 0.55 | 0.71 | 0.82 | 0.85 | 0.81 | 0.79 | 0.80 | 0.80 | 0.89 | 0.84 |
| 2*naphtoquinone | 0.45 | 0.29 | 0.18 | 0.15 | 0.19 | 0.21 | 0.20 | 0.20 | 0.11 | 0.16 |

**ID-[Zn]-ID**

# Figure S33.Structure of ID-[Zn]-ID.

**
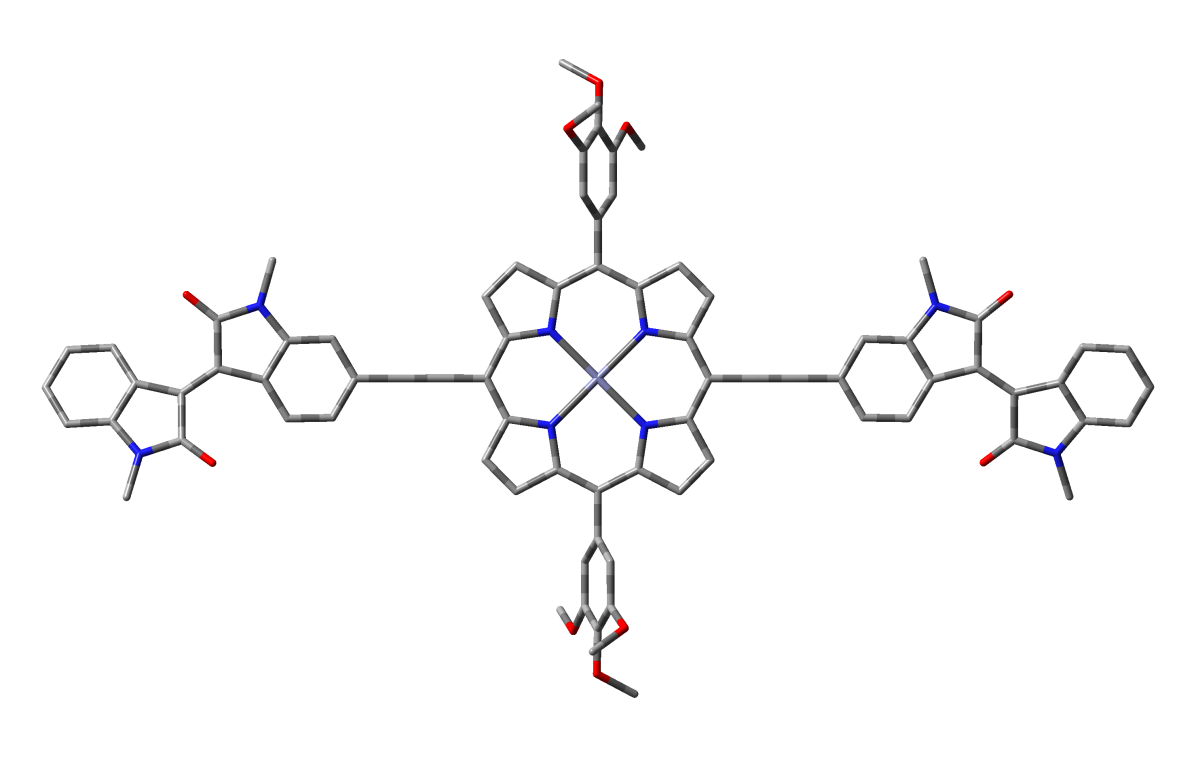
**

# Figure S34.Image of the optimized structure of ID-[Zn]-ID.


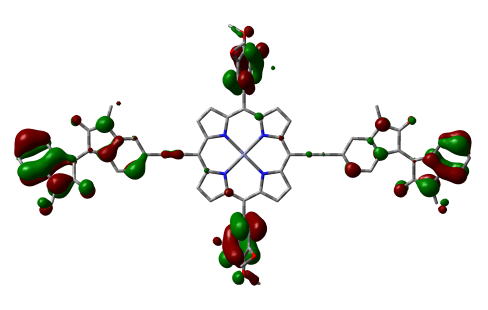


H-4 (-0.21555)


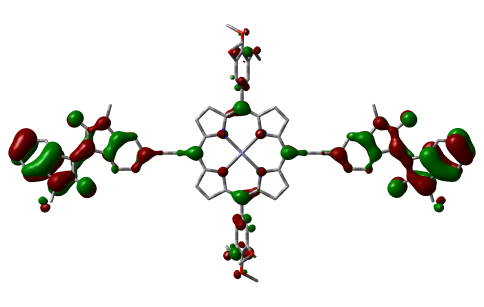


H-3 (-0.20831)


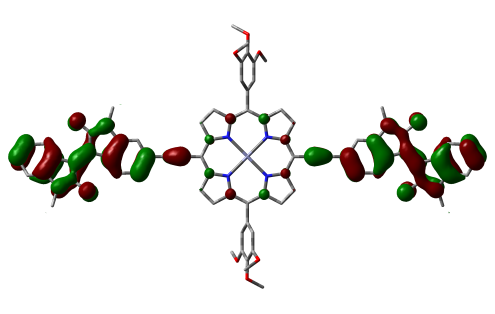


H-2 (-0.20195)

H-1 (-0.19791)

HOMO (-0.18020)

LUMO (-0.11063)

L+1 (-0.10122)

L+2 (-0.08816)

L+3 (-0.08473)

L+4 (-0.04211)

# Figure S35. Representations of the frontier MOs ofID-[Zn]-ID.The MO energies are in Hartree.

# Table S17.Computed positions of the electronic transitions, oscillator strength (f), andmajor contributions of the ID-[Zn]-ID.

| Wavelength (nm) | Osc. Strength | Major contribs |
| --- | --- | --- |
| 768.2 | 2.3488 | HOMO→LUMO (97%) |
| 647.4 | 0.0076 | HOMO→L+1 (98%) |
| 620.4 | 0.0039 | H-1→LUMO (60%), HOMO→L+3 (-36%) |
| 574.9 | 0.0287 | H-3→L+1 (-11%), H-2→LUMO (85%) |
| 561.4 | 0.0001 | H-1→L+3 (-10%), HOMO→L+2 (85%) |
| 552.1 | 0.1152 | H-3→LUMO (59%), H-2→L+1 (-20%) |
| 527.6 | 0.0002 | H-7→L+1 (-10%), H-6→L+1 (-10%), H-5→LUMO (-18%), H-4→LUMO (39%) |
| 525.0 | 0.0002 | H-1→L+1 (100%) |
| 522.1 | 0.2425 | H-1→LUMO (33%), H-1→L+2 (18%), HOMO→L+3 (39%) |
| 518.5 | 0.0762 | H-7→LUMO (20%), H-6→LUMO (19%), H-4→L+1 (-13%), H-2→L+1 (-11%) |
| 495.5 | 0.0002 | H-3→LUMO (28%), H-2→L+1 (56%) |
| 484.9 | 0.0028 | H-6→LUMO (22%), H-5→LUMO (38%), H-4→LUMO (31%) |
| 476.6 | 0.0169 | H-3→L+1 (46%) |
| 475.7 | 0.0553 | H-7→LUMO (46%), H-6→LUMO (-27%) |
| 450.7 | 1.759 | H-11→LUMO (13%), H-1→L+3 (43%) |
| 445.4 | 0.0001 | H-8→LUMO (17%), H-4→LUMO (-12%), H-3→L+1 (18%) |
| 439.7 | 0.6742 | H-1→L+2 (64%), HOMO→L+3 (-18%) |
| 438.3 | 0.0089 | H-7→LUMO (18%), H-6→LUMO (11%), H-5→L+1 (-10%), H-4→L+1 (41%) |
| 436.2 | 0.0014 | H-8→LUMO (-17%), H-2→L+2 (72%) |
| 432.5 | 0.0004 | H-8→LUMO (24%), H-6→L+1 (17%), H-5→L+1 (-16%), H-2→L+3 (18%) |
| 431.2 | 0.003 | H-9→LUMO (24%), H-5→L+1 (10%), H-2→L+3 (38%) |
| 428.4 | 0.0038 | H-9→LUMO (-25%), H-8→LUMO (-21%), H-2→L+3 (35%) |
| 427.9 | 0.0016 | H-6→L+1 (33%), H-5→L+1 (26%), H-4→L+1 (21%) |
| 425.3 | 0.018 | H-10→LUMO (72%) |
| 419.9 | 0.0001 | H-7→L+1 (64%), H-6→L+1 (-13%) |
| 414.6 | 0.0114 | H-13→LUMO (45%), H-11→LUMO (21%) |
| 411.6 | 0.016 | H-13→LUMO (35%), H-11→LUMO (-16%), H-3→L+2 (24%) |
| 407.2 | 0.0908 | H-6→L+3 (10%), H-3→L+2 (28%), H-3→L+3 (19%) |
| 401.2 | 0.0541 | H-6→L+3 (13%), H-5→L+3 (30%), H-4→L+3 (29%) |
| 399.9 | 0.1104 | H-12→LUMO (22%), H-3→L+3 (-16%) |
| 399.8 | 0.1157 | H-12→LUMO (19%), H-6→L+3 (11%), H-3→L+3 (16%) |
| 391.6 | 0.0456 | H-11→LUMO (21%), H-9→L+1 (-10%), H-8→L+1 (40%) |
| 390.9 | 0.0027 | H-6→L+2 (13%), H-5→L+2 (31%), H-4→L+2 (50%) |
| 388.1 | 0.1246 | H-14→LUMO (25%), H-7→L+3 (16%), H-6→L+3 (-11%), H-3→L+3 (13%) |
| 386.7 | 0.0091 | H-6→L+2 (53%), H-5→L+2 (-18%) |
| 385.6 | 0.0026 | H-15→LUMO (31%), H-9→L+1 (15%), H-5→L+2 (11%), H-4→L+2 (-15%) |
| 384.9 | 0.0094 | H-15→LUMO (49%) |
| 384.7 | 0.0183 | H-14→LUMO (22%), H-9→L+1 (-14%), H-8→L+1 (-13%), H-5→L+2 (16%), H-4→L+2 (-14%) |
| 382.8 | 0.0865 | H-14→LUMO (20%), H-9→L+1 (22%), H-3→L+3 (-11%) |
| 382.0 | 0.0166 | H-10→L+1 (64%) |
| 378.5 | 0.0036 | H-19→LUMO (-11%), H-16→LUMO (20%), H-12→LUMO (32%) |
| 377.4 | 0.0603 | H-7→L+2 (65%) |
| 374.4 | 0.0143 | H-19→L+1 (17%), H-18→LUMO (-16%), H-11→LUMO (-14%) |
| 372.0 | 0.0001 | H-5→L+3 (-32%), H-4→L+3 (54%) |
| 371.0 | 0.0014 | H-11→L+1 (-18%), HOMO→L+4 (49%) |
| 368.5 | 0.0494 | H-7→L+3 (58%), H-6→L+3 (20%) |
| 364.1 | 0.0044 | H-16→LUMO (17%), H-11→L+1 (20%), H-8→L+3 (12%), HOMO→L+4 (29%) |
| 362.6 | 0.0008 | H-11→L+1 (-14%), H-9→L+3 (17%), H-8→L+3 (44%) |
| 361.5 | 0.0088 | H-13→L+3 (84%) |
| 357.1 | 0.0094 | H-10→L+3 (29%), H-9→L+3 (-10%) |
| 357.0 | 0.0019 | H-23→LUMO (-12%), H-10→L+3 (20%) |
| 355.7 | 0.0385 | H-24→LUMO (27%), H-23→L+1 (-26%) |
| 355.0 | 0.0001 | H-9→L+2 (14%), H-8→L+2 (69%) |
| 352.0 | 0.0059 | H-10→L+2 (-18%), H-9→L+2 (56%) |
| 351.3 | 0.0049 | H-10→L+2 (64%) |
| 349.7 | 0.0007 | H-10→L+3 (11%), H-9→L+3 (41%), H-8→L+3 (-31%) |
| 349.1 | 0.0006 | H-13→L+1 (95%) |
| 348.8 | 0.1346 | H-18→LUMO (14%), H-17→LUMO (23%), H-12→L+1 (34%) |
| 347.9 | 0.0107 | H-24→L+1 (11%), H-23→LUMO (-13%), H-16→LUMO (33%) |
| 344.6 | 0.0055 | H-17→LUMO (32%), H-12→L+1 (-32%) |
| 339.7 | 0.0002 | H-14→L+1 (96%) |
| 337.7 | 0.0009 | H-15→L+1 (96%) |
| 335.0 | 0.0606 | H-13→L+2 (10%), H-11→L+2 (74%) |
| 331.6 | 0.0838 | H-20→LUMO (-16%), H-17→LUMO (21%), H-16→L+1 (-11%) |
| 331.3 | 0.0268 | H-13→LUMO (12%), H-13→L+2 (74%), H-11→L+2 (-10%) |
| 328.3 | 0.0132 | H-14→L+3 (73%), H-11→L+3 (12%) |
| 327.0 | 0.0262 | H-15→L+3 (20%), H-12→L+3 (-17%), H-11→L+3 (28%) |
| 326.9 | 0.0059 | H-16→L+3 (11%), H-15→L+3 (21%), H-12→L+3 (26%), H-1→L+4 (10%) |
| 326.7 | 0.0283 | H-15→L+3 (-29%), H-11→L+3 (43%) |
| 324.9 | 0.0002 | H-22→LUMO (14%), H-21→LUMO (26%), H-20→LUMO (45%) |
| 324.2 | 0.0003 | H-22→LUMO (-34%), H-21→LUMO (52%) |
| 321.8 | 0.0007 | H-12→L+3 (-12%), H-1→L+4 (65%) |
| 321.6 | 0.0062 | H-22→LUMO (10%), H-18→LUMO (-12%), H-16→L+1 (64%) |
| 321.0 | 0 | H-25→LUMO (56%) |
| 320.8 | 0.0004 | H-26→LUMO (55%), H-24→LUMO (-11%) |
| 320.1 | 0.0015 | H-15→L+3 (19%), H-12→L+2 (56%) |
| 318.7 | 0.0249 | H-14→L+2 (86%) |
| 316.8 | 0 | H-15→L+2 (78%), H-12→L+3 (10%) |
| 313.5 | 0.0006 | H-19→LUMO (17%), H-18→L+1 (37%), H-16→L+2 (11%) |
| 310.1 | 0.0184 | H-20→LUMO (-16%), H-19→L+1 (40%), H-18→LUMO (15%) |
| 309.4 | 0 | H-20→L+1 (-15%), H-19→LUMO (16%), H-18→L+1 (10%), H-17→L+1 (33%) |
| 309.0 | 0.0001 | H-19→LUMO (-14%), H-17→L+1 (47%) |
| 305.3 | 0.0101 | H-2→L+4 (89%) |
| 301.9 | 0 | H-27→LUMO (25%), H-16→L+3 (55%), H-12→L+3 (-11%) |
| 300.1 | 0 | H-21→L+1 (35%), H-20→L+1 (48%) |
| 299.6 | 0 | H-22→L+1 (-31%), H-21→L+1 (49%) |
| 298.9 | 0.0445 | H-22→L+1 (13%), H-17→L+3 (-20%), H-16→L+2 (44%) |
| 298.7 | 0.0883 | H-18→L+3 (14%), H-17→L+3 (51%), H-16→L+2 (18%) |
| 297.5 | 0.0025 | H-28→LUMO (-19%), H-25→LUMO (17%), H-23→LUMO (21%), H-22→L+1 (16%) |
| 294.4 | 0.1884 | H-31→LUMO (-10%), H-17→L+2 (11%), HOMO→L+5 (55%) |
| 293.6 | 0.0692 | H-31→LUMO (29%), H-28→L+1 (-13%), H-18→L+2 (10%), HOMO→L+5 (19%) |
| 293.1 | 0.0086 | H-24→LUMO (29%), H-23→L+1 (37%) |
| 292.4 | 0.0001 | H-3→L+4 (65%) |
| 291.4 | 0.0004 | H-27→LUMO (50%), H-16→L+3 (-17%) |
| 291.3 | 0.0037 | H-18→L+2 (13%), H-17→L+2 (64%) |
| 290.8 | 0.0011 | H-28→LUMO (18%), H-24→L+1 (26%), H-3→L+4 (-18%) |
| 289.2 | 0.0017 | HOMO→L+6 (83%) |
| 286.5 | 0.0001 | H-29→LUMO (90%) |
| 285.9 | 0.0001 | H-30→LUMO (87%) |
| 285.4 | 0.0012 | H-6→L+4 (19%), H-5→L+4 (33%), H-4→L+4 (42%) |

# Figure S36.Bar graph (blue) reporting the position of the electronic transitions vs the oscillator strength (f) for ID-[Zn]-ID.The black line represents a spectrum where 1000 cm-1 is applied to each transition.

# Table S18.Atomic contributions for the frontier MO for ID-[Zn]-ID.

|  | H-4 | H-3 | H-2 | H-1 | H | L | L+1 | L+2 | L+3 | L+4 |
| --- | --- | --- | --- | --- | --- | --- | --- | --- | --- | --- |
| 2* porphyrins | 0.43 | 0.19 | 0.14 | ~1.00 | 0.74 | 0.40 | 0.07 | 0.54 | ~1.00 | 0.78 |
| 2*isoindigo | 0.57 | 0.81 | 0.86 | ~0.0 | 0.26 | 0.60 | 0.93 | 0.46 | ~0.0 | 0.22 |

**NQ-[S]-[Zn]-[S]-NQ**

# Figure S37.Structure of NQ-[S]-[Zn]-[S]-NQ.

# Figure S38.Image of the optimized structure of NQ-[S]-[Zn]-[S]-NQ.

H-4 (-0.21270)

H-3 (-0.20301)

H-2 (-0.19815)

H-1 (-0.19590)

HOMO (-0.17723)

LUMO (-0.10913)

L+1 (-0.10404)

L+2 (-0.09719)

L+3 (-0.08485)

L+4 (-0.06476)

#

# Figure S39. Representations of the frontier MOs of NQ-[S]-[Zn]-[S]-NQ.The MO energies are in Hartree.

# Table S19.Computed positions of the electronic transitions, oscillator strength (f), and major contributions of the NQ-[S]-[Zn]-[S]-NQ.

| Wavelength (nm) | Osc. Strength | Major contribs |
| --- | --- | --- |
| 785.6 | 3.1605 | HOMO→LUMO (95%) |
| 688.2 | 0.0081 | HOMO→L+1 (98%) |
| 628.8 | 0.0873 | HOMO→L+2 (93%) |
| 620.0 | 0.0013 | H-2→LUMO (-39%), HOMO→L+3 (51%) |
| 594.9 | 0.0309 | H-3→L+1 (-10%), H-1→LUMO (86%) |
| 567.7 | 0.6192 | H-3→LUMO (-37%), H-1→L+1 (45%) |
| 535.5 | 0.0159 | H-5→L+1 (20%), H-4→LUMO (33%), H-3→L+1 (-18%) |
| 531.7 | 0.0744 | H-5→LUMO (-13%), H-4→L+1 (-18%), H-3→LUMO (17%), H-1→L+1 (31%) |
| 525.8 | 0.1653 | H-2→LUMO (55%), H-2→L+2 (-17%), HOMO→L+3 (22%) |
| 522.4 | 0.0008 | H-2→L+1 (98%) |
| 521.1 | 0.078 | H-5→LUMO (18%), H-3→LUMO (33%), H-1→L+1 (11%) |
| 515.9 | 0.0155 | H-8→LUMO (11%), H-3→L+1 (18%), H-1→L+2 (39%) |
| 491.7 | 0.0008 | H-3→L+1 (-34%), H-1→L+2 (50%) |
| 484.9 | 0.733 | H-8→L+1 (-13%), H-7→LUMO (15%), H-3→L+2 (26%), H-2→L+3 (-14%) |
| 477.0 | 0.0031 | H-6→LUMO (66%), H-4→LUMO (-16%) |
| 473.0 | 0.5046 | H-7→LUMO (-16%), H-2→L+2 (38%), HOMO→L+3 (12%) |
| 469.7 | 0.0029 | H-8→LUMO (28%), H-6→LUMO (-19%), H-4→LUMO (-25%), H-3→L+1 (-11%) |
| 463.8 | 0.3149 | H-7→LUMO (27%), H-5→LUMO (17%), H-2→L+2 (25%), HOMO→L+3 (12%) |
| 452.8 | 0.0008 | H-1→L+3 (95%) |
| 451.8 | 0.3624 | H-9→LUMO (-13%), H-8→L+1 (10%), H-4→L+1 (-21%), H-3→L+2 (38%) |
| 450.3 | 0.0011 | HOMO→L+4 (83%) |
| 447.2 | 0.3463 | H-5→LUMO (-20%), H-4→L+1 (24%), H-2→L+3 (17%) |
| 438.6 | 0.001 | H-8→LUMO (-16%), H-5→L+1 (41%), H-4→LUMO (-17%), H-4→L+2 (-10%) |
| 433.4 | 0.2686 | H-7→LUMO (-12%), H-6→L+1 (-13%), H-5→LUMO (10%), H-2→L+3 (24%), HOMO→L+5 (10%) |
| 430.9 | 0.0362 | H-6→L+1 (80%) |
| 426.1 | 0.0002 | H-8→LUMO (21%), H-7→L+1 (63%) |
| 423.5 | 0.0002 | H-10→LUMO (80%) |
| 421.2 | 0.0048 | H-11→LUMO (-21%), H-9→LUMO (42%), H-8→L+1 (15%) |
| 420.0 | 0 | H-5→L+1 (14%), H-4→L+2 (49%) |
| 416.2 | 0.0059 | H-6→L+2 (43%) |
| 415.7 | 0.1631 | H-11→LUMO (-20%), H-5→L+2 (29%) |
| 413.4 | 0.2258 | H-3→L+3 (63%) |
| 412.8 | 0.0118 | H-6→L+2 (34%), H-4→L+2 (16%) |
| 412.2 | 0.0873 | H-11→LUMO (12%), H-7→L+2 (22%), H-5→L+2 (30%) |
| 410.0 | 0.0302 | H-14→LUMO (50%), H-14→L+2 (22%), H-13→LUMO (12%) |
| 404.7 | 0.151 | H-14→LUMO (12%), H-11→LUMO (18%), H-7→L+2 (-11%), HOMO→L+5 (30%) |
| 402.7 | 0.0006 | H-6→L+3 (85%) |
| 401.7 | 0.0039 | H-8→L+1 (-11%), H-7→L+2 (42%), H-5→L+2 (-10%) |
| 399.9 | 0.0001 | H-12→LUMO (-15%), H-9→L+1 (17%), H-8→L+2 (29%) |
| 395.9 | 0.047 | H-7→L+3 (38%), H-5→L+3 (21%), H-3→L+3 (-15%) |
| 391.0 | 0 | H-9→L+1 (44%), H-8→L+2 (-43%) |
| 387.5 | 0.0074 | H-10→L+1 (89%) |
| 385.6 | 0.0259 | H-9→L+2 (15%), HOMO→L+5 (19%) |
| 383.0 | 0.0005 | H-17→LUMO (25%), H-12→LUMO (33%) |
| 382.3 | 0 | H-16→LUMO (40%), H-16→L+2 (11%), H-4→L+3 (-33%) |
| 380.0 | 0.0148 | H-15→LUMO (70%), H-15→L+2 (17%) |
| 379.2 | 0.0018 | H-11→L+1 (52%), H-4→L+3 (-11%) |
| 378.5 | 0.2524 | H-9→L+2 (31%), H-1→L+4 (37%) |
| 378.2 | 0.0088 | H-16→LUMO (-19%), H-11→L+1 (-18%), H-4→L+3 (-18%), H-2→L+4 (28%) |
| 376.3 | 0.2238 | H-9→L+2 (26%), H-1→L+4 (-23%) |
| 375.3 | 0.0001 | H-16→LUMO (13%), H-10→L+2 (-14%), H-4→L+3 (15%), H-2→L+4 (47%) |
| 373.9 | 0.0003 | H-10→L+2 (71%), H-2→L+4 (14%) |
| 369.4 | 0.0069 | H-20→L+1 (13%), H-17→LUMO (42%), H-12→LUMO (-11%) |
| 368.3 | 0.0829 | H-11→L+3 (-14%), H-9→L+3 (24%), H-7→L+3 (-12%), H-5→L+3 (34%) |
| 367.6 | 0.0202 | H-13→LUMO (35%), H-12→L+1 (-23%), H-11→L+2 (28%) |
| 364.7 | 0.0003 | H-10→L+3 (90%) |
| 363.3 | 0.0124 | H-12→L+1 (33%), H-11→L+2 (41%) |
| 362.2 | 0.002 | H-14→L+3 (69%) |
| 360.9 | 0.0032 | H-13→L+1 (25%), H-12→L+2 (-12%), H-3→L+4 (21%) |
| 359.7 | 0.0016 | H-14→L+3 (-22%), H-9→L+3 (14%), H-7→L+3 (24%), H-5→L+3 (-16%) |
| 357.1 | 0 | H-8→L+3 (84%) |
| 355.9 | 0.0247 | H-9→L+3 (13%), H-1→L+4 (10%) |
| 353.6 | 0.0002 | H-28→L+1 (21%), H-27→LUMO (24%), H-27→L+2 (-11%), H-26→L+1 (13%), H-3→L+4 (12%) |
| 352.8 | 0.0073 | H-28→LUMO (13%), H-27→L+1 (28%) |
| 350.2 | 0.0065 | H-12→L+2 (27%), H-3→L+4 (43%), HOMO→L+6 (10%) |
| 348.9 | 0.0005 | H-14→L+1 (10%), H-13→L+1 (38%), H-12→L+2 (36%) |
| 347.0 | 0.0005 | H-14→L+1 (81%) |
| 343.0 | 0.0207 | H-11→L+3 (19%), H-9→L+3 (14%), H-2→L+5 (57%) |
| 342.8 | 0.0021 | HOMO→L+6 (70%) |
| 342.4 | 0.0716 | H-18→LUMO (51%), H-18→L+2 (11%), H-17→L+1 (-10%) |
| 341.4 | 0.0106 | H-14→LUMO (-22%), H-14→L+2 (40%), H-13→L+2 (29%) |
| 339.4 | 0.0177 | H-17→L+1 (30%), H-14→L+2 (-23%), H-13→L+2 (21%) |
| 338.8 | 0.0003 | H-15→L+1 (92%) |
| 337.4 | 0.0003 | H-1→L+5 (76%) |
| 337.2 | 0.001 | H-16→L+1 (90%) |
| 335.4 | 0.2546 | H-11→L+3 (-34%), H-9→L+3 (-21%), H-2→L+5 (35%) |
| 333.6 | 0.0239 | H-17→L+1 (47%), H-13→L+2 (-27%) |
| 331.4 | 0.0002 | H-17→L+3 (28%), H-16→LUMO (-14%), H-16→L+2 (31%), H-12→L+3 (20%) |
| 330.3 | 0.0174 | H-15→LUMO (-16%), H-15→L+2 (63%) |
| 330.1 | 0.0106 | H-17→LUMO (-13%), H-17→L+2 (37%), H-16→L+3 (11%) |
| 328.0 | 0.0178 | H-15→L+3 (73%), H-4→L+4 (11%) |
| 327.5 | 0.0003 | H-17→L+3 (-20%), H-16→L+2 (43%), H-12→L+3 (-23%) |
| 326.0 | 0.002 | H-15→L+3 (-12%), H-4→L+4 (63%) |
| 325.0 | 0.0008 | H-21→LUMO (11%), H-19→LUMO (28%), H-16→L+3 (41%) |
| 323.2 | 0.0007 | H-6→L+4 (50%), H-5→L+4 (-33%) |
| 322.9 | 0.0003 | H-6→L+4 (39%), H-5→L+4 (42%) |
| 322.4 | 0 | H-19→LUMO (15%), H-17→L+2 (35%), H-16→L+3 (-35%) |
| 321.4 | 0.0354 | H-19→L+1 (10%), H-4→L+4 (-11%), H-3→L+5 (37%) |
| 318.8 | 0.0005 | H-22→LUMO (82%) |
| 318.5 | 0.0016 | H-7→L+4 (76%) |
| 318.2 | 0.0002 | H-23→LUMO (80%) |
| 317.1 | 0.0569 | H-24→LUMO (12%), H-19→L+1 (-14%), H-3→L+5 (43%) |
| 316.3 | 0.0229 | H-26→LUMO (10%), H-24→LUMO (69%) |
| 316.1 | 0.0003 | H-17→L+3 (-40%), H-12→L+3 (51%) |
| 315.9 | 0.0014 | H-25→LUMO (83%) |
| 313.1 | 0.002 | H-29→LUMO (-31%), H-8→L+4 (37%) |
| 312.7 | 0.0013 | H-30→LUMO (23%), H-29→LUMO (19%), H-8→L+4 (25%) |
| 312.4 | 0.0013 | H-30→LUMO (39%), H-8→L+4 (-17%) |
| 310.2 | 0.0768 | H-13→L+3 (86%) |
| 309.1 | 0.0001 | H-21→LUMO (-16%), H-20→L+1 (12%), H-19→LUMO (15%), H-19→L+2 (21%) |

# Figure S40.Bar graph (blue) reporting the position of the electronic transitions vs the oscillator strength (f) for NQ-[S]-[Zn]-[S]-NQ.The black line represents a spectrum where 1000 cm-1 is applied to each transition.

# Table S20.Atomic contributions for the frontier MO for NQ-[S]-[Zn]-[S]-NQ.

|  | H-4 | H-3 | H-2 | H-1 | H | L | L+1 | L+2 | L+3 | L+4 |
| --- | --- | --- | --- | --- | --- | --- | --- | --- | --- | --- |
| 2* thiopene-porphyrins | 0.23 | 0.28 | ~1.00 | 0.46 | 0.90 | 0.52 | 0.12 | 0.54 | ~1.00 | 0.82 |
| 2*isoindigo | 0.77 | 0.72 | ~0.0 | 0.54 | 0.10 | 0.48 | 0.88 | 0.46 | ~0.0 | 0.18 |

**ID-[B-Zn]-ID**

# Figure S41.Structure of ID-[B-Zn]-ID.

# Figure S42.Image of the optimized structure of ID-[B-Zn]-ID.

H-4 (-0.21462)

H-3 (-0.20780)

H-2 (-0.20043)

H-1 (-0.18018)

HOMO (-0.17426)

LUMO (-0.10838)

L+1 (-0.10147)

L+2 (-0.08638)

L+3 (-0.08205)

L+4 (-0.04297)

# Figure S43.Representations of the frontier MOs of ID-[B-Zn]-ID.The MO energies are in Hartree.

# Table S21.Computed positions of the electronic transitions, oscillator strength (f), and major contributions of the ID-[B-Zn]-ID.

| Wavelength (nm) | Osc. Strength | Major contribs |
| --- | --- | --- |
| 806.4 | 0.1133 | HOMO→LUMO (96%) |
| 755.5 | 1.7423 | H-1→LUMO (93%) |
| 724.8 | 0.0011 | HOMO→L+1 (100%) |
| 653.0 | 0.0006 | H-1→L+1 (98%) |
| 614.8 | 0.0458 | H-1→L+3 (-23%), HOMO→L+2 (75%) |
| 602.2 | 0.7821 | H-1→L+2 (35%), HOMO→L+3 (55%) |
| 571.1 | 0.0002 | H-3→L+1 (-11%), H-2→LUMO (84%) |
| 550.6 | 0.2305 | H-3→LUMO (47%), H-2→L+1 (-26%), H-1→L+2 (11%) |
| 526.2 | 0.0008 | H-7→L+1 (-15%), H-6→L+1 (-11%), H-5→LUMO (25%), H-4→LUMO (30%) |
| 519.6 | 0.2018 | H-7→LUMO (24%), H-6→LUMO (17%), H-5→L+1 (-13%), H-4→L+1 (-13%), H-2→L+1 (-15%) |
| 500.7 | 0.629 | H-2→L+1 (29%), H-1→L+2 (31%), HOMO→L+3 (-22%) |
| 494.7 | 1.0347 | H-1→L+3 (68%), HOMO→L+2 (23%) |
| 490.7 | 0.6947 | H-3→LUMO (35%), H-2→L+1 (18%), HOMO→L+3 (10%) |
| 481.5 | 0 | H-8→LUMO (13%), H-5→LUMO (-15%), H-3→L+1 (34%) |
| 474.9 | 0.0011 | H-5→LUMO (-32%), H-4→LUMO (41%), H-3→L+1 (-14%) |
| 469.7 | 0.0519 | H-7→LUMO (-36%), H-6→LUMO (56%) |
| 448.7 | 0.0011 | H-8→LUMO (34%), H-5→LUMO (14%), H-3→L+1 (-26%) |
| 438.7 | 0 | H-8→LUMO (-10%), H-2→L+2 (85%) |
| 436.3 | 0.0152 | H-4→L+1 (70%) |
| 432.1 | 0.0097 | H-7→LUMO (10%), H-5→L+1 (77%) |
| 431.3 | 0 | H-6→L+1 (78%) |
| 430.8 | 0.0022 | H-9→LUMO (-14%), H-2→L+3 (74%) |
| 425.1 | 0.0634 | H-10→LUMO (58%), H-8→L+1 (-10%) |
| 424.8 | 0.0031 | H-9→LUMO (41%), H-8→LUMO (22%), H-7→L+1 (-14%), H-2→L+3 (14%) |
| 420.7 | 0 | H-9→LUMO (27%), H-7→L+1 (47%) |
| 414.6 | 0.0681 | H-11→LUMO (33%), H-10→LUMO (-32%), H-8→L+1 (-10%) |
| 407.4 | 0.042 | H-3→L+2 (85%) |
| 406.5 | 0.0056 | H-13→LUMO (18%), H-12→LUMO (47%) |
| 403.5 | 0 | H-13→LUMO (-27%), H-12→LUMO (40%) |
| 401.2 | 0.0028 | H-11→LUMO (14%), H-8→L+1 (58%) |
| 397.8 | 0.1122 | H-6→L+3 (27%), H-3→L+3 (66%) |
| 393.1 | 0.0048 | H-5→L+2 (-10%), H-5→L+3 (-33%), H-4→L+2 (12%), H-4→L+3 (36%) |
| 392.7 | 0.0015 | H-14→LUMO (81%) |
| 392.6 | 0.0023 | H-5→L+2 (-20%), H-5→L+3 (13%), H-4→L+2 (32%), H-4→L+3 (-15%), HOMO→L+4 (16%) |
| 390.3 | 0.0472 | H-4→L+2 (-14%), HOMO→L+4 (71%) |
| 388.2 | 0.0101 | H-9→L+1 (76%) |
| 387.1 | 0.0001 | H-15→LUMO (-22%), H-13→LUMO (12%), H-10→L+1 (52%) |
| 387.0 | 0.0053 | H-6→L+2 (73%) |
| 386.8 | 0.0003 | H-15→LUMO (38%), H-10→L+1 (37%) |
| 384.7 | 0.0209 | H-21→LUMO (13%), H-16→LUMO (26%), H-11→LUMO (-18%) |
| 383.1 | 0.0476 | H-7→L+3 (-21%), H-6→L+3 (46%), H-3→L+3 (-27%) |
| 381.5 | 0.0001 | H-5→L+2 (54%), H-4→L+2 (34%) |
| 376.2 | 0.0071 | H-19→LUMO (20%), H-7→L+2 (55%) |
| 375.8 | 0.0174 | H-17→LUMO (81%) |
| 375.3 | 0.0003 | H-1→L+4 (63%) |
| 374.9 | 0.0396 | H-19→LUMO (45%), H-16→LUMO (10%), H-7→L+2 (-21%) |
| 371.7 | 0 | H-15→LUMO (14%), H-13→LUMO (24%), H-11→L+1 (40%) |
| 369.4 | 0.1054 | H-18→LUMO (55%), H-12→L+1 (19%) |
| 368.4 | 0.0023 | H-18→LUMO (-13%), H-12→L+1 (72%) |
| 368.0 | 0.0074 | H-8→L+3 (10%), H-5→L+3 (41%), H-4→L+3 (36%) |
| 367.2 | 0.1895 | H-21→LUMO (-17%), H-16→LUMO (42%) |
| 365.2 | 0.0018 | H-21→L+1 (-12%), H-20→LUMO (15%), H-11→L+1 (11%), H-1→L+4 (23%) |
| 361.8 | 0.0155 | H-7→L+3 (69%), H-6→L+3 (21%) |
| 361.5 | 0.0033 | H-11→L+1 (-10%), H-8→L+2 (77%) |
| 359.2 | 0.0016 | H-9→L+3 (21%), H-8→L+3 (57%) |
| 357.8 | 0.0534 | H-13→L+1 (65%) |
| 356.0 | 0.0025 | H-14→L+1 (86%) |
| 354.9 | 0.0001 | H-30→L+1 (11%), H-29→LUMO (-16%), H-27→L+1 (-14%), H-26→LUMO (24%) |
| 354.3 | 0.0029 | H-10→L+2 (25%), H-10→L+3 (55%) |
| 354.1 | 0.0019 | H-30→LUMO (-10%), H-29→L+1 (17%), H-27→LUMO (15%), H-26→L+1 (-14%), H-24→LUMO (-10%) |
| 353.9 | 0.0019 | H-9→L+2 (64%), HOMO→L+5 (11%) |
| 352.8 | 0.0033 | H-10→L+2 (67%), H-10→L+3 (-24%) |
| 350.2 | 0.0055 | H-9→L+2 (-18%), H-9→L+3 (42%), H-8→L+3 (-19%) |
| 348.8 | 0.0001 | H-9→L+3 (-15%), HOMO→L+5 (56%) |
| 347.2 | 0.0717 | H-15→L+1 (79%) |
| 345.9 | 0.009 | H-17→L+1 (93%) |
| 344.7 | 0.0002 | H-16→L+1 (79%) |
| 341.2 | 0.0016 | H-12→L+2 (72%) |
| 339.7 | 0.0001 | H-18→L+1 (-20%), H-13→L+2 (10%), H-12→L+3 (48%) |
| 338.1 | 0.005 | H-1→L+5 (34%), HOMO→L+7 (47%) |
| 338.0 | 0.0072 | HOMO→L+6 (90%) |
| 337.5 | 0 | H-18→L+1 (69%), H-12→L+3 (19%) |
| 337.2 | 0.0472 | H-11→L+2 (83%) |
| 334.2 | 0.0099 | H-19→L+3 (-18%), H-14→L+3 (54%) |
| 333.6 | 0.0001 | H-26→LUMO (-12%), H-19→L+1 (65%) |
| 333.6 | 0.0193 | H-19→L+3 (58%), H-14→L+3 (19%), H-11→L+3 (13%) |
| 331.8 | 0.0444 | H-1→L+5 (-40%), HOMO→L+7 (45%) |
| 331.3 | 0.0004 | H-14→L+2 (66%), H-11→L+3 (-10%) |
| 329.8 | 0.0002 | H-26→LUMO (18%), H-22→LUMO (10%), H-19→L+1 (26%), H-13→L+2 (-13%) |
| 328.3 | 0.001 | H-15→L+3 (38%), H-13→L+3 (-32%) |
| 327.8 | 0.0141 | H-30→LUMO (-10%), H-24→LUMO (18%), H-23→LUMO (-17%), H-1→L+6 (-17%) |
| 327.5 | 0.0017 | H-22→LUMO (26%), H-13→L+2 (22%) |
| 325.8 | 0.0007 | H-29→LUMO (13%), H-26→LUMO (17%), H-13→L+2 (33%) |
| 325.1 | 0.0275 | H-19→L+3 (-15%), H-14→L+2 (17%), H-11→L+3 (54%) |
| 324.2 | 0.0023 | H-27→LUMO (11%), HOMO→L+8 (43%) |
| 323.7 | 0.0221 | H-30→LUMO (-19%), H-27→LUMO (-10%), HOMO→L+8 (36%) |
| 322.3 | 0.0001 | H-17→L+3 (36%), H-1→L+7 (48%) |
| 320.6 | 0.0258 | H-23→LUMO (-17%), H-1→L+6 (55%) |
| 319.6 | 0.0132 | H-17→L+2 (73%), H-13→L+3 (16%) |
| 318.3 | 0.0009 | H-15→L+2 (59%), H-1→L+7 (17%) |
| 318.0 | 0.0004 | H-16→L+2 (67%) |
| 315.7 | 0.0003 | H-25→LUMO (81%), H-22→LUMO (-12%) |
| 315.4 | 0.0353 | H-24→LUMO (22%), H-23→LUMO (31%), H-18→L+3 (-10%) |
| 314.9 | 0.0484 | H-18→L+3 (55%), H-1→L+8 (-16%) |
| 314.3 | 0.008 | H-19→LUMO (-11%), H-19→L+2 (55%) |
| 314.1 | 0.0096 | H-17→L+3 (42%), H-1→L+7 (-25%) |
| 313.7 | 0.0258 | H-15→L+3 (42%), H-13→L+3 (28%) |
| 313.4 | 0.0852 | H-16→L+3 (71%), H-11→L+3 (11%) |
| 312.8 | 0.0801 | H-18→L+2 (71%) |
| 312.1 | 0.0041 | H-2→L+4 (66%) |

# Figure S44.Bar graph (blue) reporting the position of the electronic transitions vs the oscillator strength (f) for ID-[B-Zn]-ID.The black line represents a spectrum where 1000 cm-1 is applied to each transition.

# Table S22.Atomic contributions for the frontier MO for ID-[B-Zn]-ID.

|  | H-4 | H-3 | H-2 | H-1 | H | L | L+1 | L+2 | L+3 | L+4 |
| --- | --- | --- | --- | --- | --- | --- | --- | --- | --- | --- |
| Benzo-porphyrins | 0.55 | 0.21 | 0.22 | 0.76 | 0.99 | 0.34 | 0.08 | 0.65 | 0.99 | 0.77 |
| 2*isoindigo | 0.45 | 0.79 | 0.78 | 0.24 | 0.01 | 0.66 | 0.92 | 0.35 | 0.01 | 0.23 |

**ID-[S]-[B-Zn]-[S]-ID**

# Figure S45.Structure of ID-[S]-[B-Zn]-[S]-ID.

# Figure S46.Image of the optimized structure of ID-[S]-[B-Zn]-[S]-ID.

H-4 (-0.21146)

H-3 (-0.20248)

H-2 (-0.19472)

H-1 (-0.17809)

HOMO (-0.17460)

**Figure S47.Representations of the frontier MOs ofID-[S]-[B-Zn]-[S]-ID.The MO energies are in Hartree.**

LUMO (-0.10726)

L+1 (-0.10412)

L+2 (-0.09496)

L+3 (-0.08241)

L+4 (-0.06564)

#

# Table S23.Computed positions of the electronic transitions, oscillator strength (f), and major contributions of the ID-[S]-[B-Zn]-[S]-ID.

| Wavelength (nm) | Osc. Strength | Major contribs |
| --- | --- | --- |
| 770.2 | 0.3003 | HOMO→LUMO (88%) |
| 758.4 | 2.3379 | H-1→LUMO (86%) |
| 716.3 | 0.0004 | HOMO→L+1 (100%) |
| 682.3 | 0.0041 | H-1→L+1 (97%) |
| 659.2 | 0.0645 | H-1→L+3 (-13%), HOMO→L+2 (79%) |
| 635.6 | 0.1797 | H-1→L+2 (62%), HOMO→L+3 (27%) |
| 590.9 | 0.021 | H-3→L+1 (12%), H-2→LUMO (83%) |
| 577.7 | 1.6795 | H-3→LUMO (27%), H-2→L+1 (44%), HOMO→L+3 (12%) |
| 538.4 | 0.9125 | H-4→L+1 (10%), H-2→L+1 (-11%), H-1→L+2 (-19%), HOMO→L+3 (38%) |
| 535.4 | 0.0139 | H-5→L+1 (17%), H-4→LUMO (34%), H-3→L+1 (-18%) |
| 532.6 | 0.0126 | H-5→LUMO (11%), H-4→L+1 (17%), H-3→LUMO (-18%), H-2→L+1 (29%) |
| 516.6 | 0.7905 | H-1→L+3 (78%), HOMO→L+2 (17%) |
| 513.6 | 0.0209 | H-8→LUMO (-17%), H-3→L+1 (21%), H-2→L+2 (31%) |
| 511.9 | 0.1979 | H-8→L+1 (-18%), H-3→LUMO (38%) |
| 494.8 | 0.0004 | H-3→L+1 (-25%), H-2→L+2 (60%) |
| 472.7 | 0.0041 | H-8→LUMO (10%), H-4→LUMO (31%), H-3→L+1 (12%), HOMO→L+4 (27%) |
| 472.2 | 0.0085 | H-4→LUMO (-12%), HOMO→L+4 (70%) |
| 468.4 | 0.0524 | H-7→LUMO (25%), H-5→LUMO (-20%), H-4→L+1 (-11%) |
| 464.5 | 0.0009 | H-6→LUMO (71%), H-6→L+2 (-11%) |
| 455.5 | 0.0192 | H-7→LUMO (13%), H-5→LUMO (-26%), H-4→L+1 (27%) |
| 452.9 | 0.2076 | H-3→L+2 (79%) |
| 452.5 | 0.0013 | H-1→L+4 (90%) |
| 449.8 | 0.0083 | H-2→L+3 (89%) |
| 437.5 | 0.0015 | H-5→L+1 (58%), H-4→LUMO (-14%) |
| 436.0 | 0.0706 | H-9→LUMO (18%), H-7→LUMO (17%), H-6→L+1 (19%), H-5→LUMO (11%), H-4→L+1 (-16%) |
| 434.0 | 0.0065 | H-6→L+1 (76%) |
| 426.0 | 0.0002 | H-7→L+1 (56%) |
| 423.8 | 0.0019 | H-10→LUMO (22%), H-8→LUMO (27%), H-4→L+2 (-11%) |
| 423.5 | 0.0884 | H-3→L+3 (11%), HOMO→L+5 (85%) |
| 418.0 | 0.0019 | H-10→LUMO (11%), H-6→L+2 (-29%), H-4→L+2 (42%) |
| 416.6 | 0.1433 | H-9→LUMO (-22%), H-8→L+1 (34%) |
| 414.9 | 0.1301 | H-11→LUMO (26%), H-9→LUMO (-18%) |
| 414.6 | 0.0252 | H-10→LUMO (33%), H-6→L+2 (15%) |
| 413.2 | 0.0313 | H-7→L+2 (-20%), H-5→L+2 (44%) |
| 412.9 | 0.0028 | H-6→L+2 (35%), H-4→L+2 (18%) |
| 407.7 | 0.3576 | H-3→L+3 (57%), H-1→L+5 (11%) |
| 406.9 | 0.0712 | H-11→LUMO (-29%), H-3→L+3 (-15%), H-1→L+5 (33%) |
| 399.5 | 0.0001 | H-9→L+1 (38%), H-8→L+2 (11%) |
| 398.9 | 0.0017 | H-7→L+2 (50%), H-5→L+2 (17%) |
| 397.3 | 0.0016 | H-13→LUMO (66%), H-13→L+2 (-18%) |
| 395.5 | 0.0186 | H-11→LUMO (10%), H-1→L+5 (31%) |
| 394.5 | 0.0037 | H-6→L+3 (83%) |
| 392.3 | 0.0041 | H-10→L+1 (89%) |
| 391.5 | 0.0006 | H-9→L+1 (-17%), H-8→L+2 (65%) |
| 389.1 | 0.0077 | H-7→L+3 (42%), H-5→L+3 (-39%) |
| 386.2 | 0.0001 | H-16→LUMO (35%), H-12→LUMO (-19%) |
| 384.4 | 0.0037 | H-15→LUMO (69%), H-15→L+2 (-20%) |
| 383.5 | 0.0318 | H-11→L+1 (73%) |
| 383.4 | 0.4771 | H-2→L+4 (70%) |
| 379.6 | 0.0034 | H-9→L+2 (70%) |
| 378.6 | 0.0014 | H-10→L+2 (-20%), H-4→L+3 (49%) |
| 376.6 | 0.0031 | H-10→L+2 (37%), H-4→L+3 (22%) |
| 374.9 | 0.0032 | H-16→LUMO (-19%), H-12→LUMO (-15%), H-10→L+2 (26%) |
| 372.9 | 0.0544 | H-12→L+1 (26%), H-11→L+2 (-14%) |
| 370.5 | 0.0004 | H-14→LUMO (41%), H-12→L+1 (32%) |
| 369.6 | 0.0027 | H-17→LUMO (66%), H-17→L+2 (-16%) |
| 369.0 | 0.0398 | H-21→LUMO (26%), H-21→L+2 (-19%), H-14→LUMO (10%), H-13→L+1 (12%), H-11→L+2 (-12%) |
| 367.4 | 0.0115 | H-13→L+1 (77%) |
| 366.5 | 0.0022 | H-14→L+1 (19%), H-3→L+4 (23%), H-2→L+5 (12%) |
| 366.2 | 0.0102 | H-21→LUMO (10%), H-11→L+2 (46%) |
| 362.2 | 0.0362 | H-20→LUMO (-13%), H-11→L+3 (-11%), H-9→L+3 (18%), H-7→L+3 (16%), H-5→L+3 (21%) |
| 361.1 | 0.0537 | H-20→LUMO (28%), H-19→LUMO (-12%), H-7→L+3 (10%), H-5→L+3 (10%) |
| 360.5 | 0.0215 | H-10→L+3 (54%), HOMO→L+6 (-22%) |
| 358.7 | 0.0281 | H-15→L+1 (12%), H-10→L+3 (18%), HOMO→L+6 (43%) |
| 357.4 | 0.0002 | H-15→L+1 (-13%), H-13→LUMO (14%), H-13→L+2 (52%) |
| 356.4 | 0.0067 | H-14→L+1 (-33%), H-3→L+4 (33%) |
| 356.3 | 0.0127 | H-20→LUMO (11%) |
| 355.0 | 0.0028 | H-18→LUMO (16%) |
| 354.6 | 0.0014 | H-11→L+3 (37%), H-9→L+3 (-22%), H-7→L+3 (21%), H-5→L+3 (15%) |
| 354.3 | 0.0033 | H-15→L+1 (-19%), H-12→L+2 (26%), H-1→L+6 (14%) |
| 353.7 | 0.0007 | H-15→L+1 (-13%), H-1→L+6 (-16%), HOMO→L+7 (53%) |
| 353.2 | 0.0041 | H-15→L+1 (23%), H-13→L+2 (11%), H-12→L+2 (17%), HOMO→L+7 (12%) |
| 351.6 | 0.0084 | H-10→L+3 (-11%), H-8→L+3 (54%) |
| 351.4 | 0.0344 | H-16→L+1 (64%) |
| 351.1 | 0.0014 | H-18→LUMO (-11%), H-12→L+2 (18%) |
| 350.3 | 0.0024 | H-32→L+1 (11%), H-19→LUMO (26%) |
| 346.5 | 0.0007 | H-17→L+1 (44%), H-15→LUMO (-10%), H-15→L+2 (-39%) |
| 345.0 | 0.0012 | H-2→L+5 (63%), H-1→L+6 (13%) |
| 343.2 | 0.0008 | H-17→L+1 (53%), H-15→LUMO (13%), H-15→L+2 (26%) |
| 342.5 | 0.001 | H-16→LUMO (11%), H-16→L+2 (41%), H-13→L+3 (22%) |
| 341.0 | 0.0086 | H-14→L+2 (51%), H-11→L+3 (11%), H-9→L+3 (13%) |
| 340.9 | 0.004 | H-14→L+2 (24%), H-11→L+3 (-21%), H-9→L+3 (-24%) |
| 340.5 | 0.0049 | H-18→LUMO (-10%), H-1→L+6 (36%), HOMO→L+7 (10%) |
| 339.0 | 0.0132 | H-18→L+1 (51%) |
| 338.7 | 0.0431 | H-1→L+7 (58%), HOMO→L+6 (-11%) |
| 336.9 | 0.0017 | H-19→L+1 (65%) |
| 336.4 | 0.0012 | H-20→L+1 (66%), H-13→L+3 (20%) |
| 334.6 | 0.0189 | H-17→LUMO (14%), H-17→L+2 (48%), H-16→L+3 (10%), H-1→L+7 (-13%) |
| 334.1 | 0.0007 | H-20→L+1 (-19%), H-16→L+2 (-23%), H-13→L+3 (39%) |
| 334.1 | 0.006 | H-15→L+3 (41%), H-4→L+4 (30%) |
| 333.6 | 0 | H-18→L+1 (-10%), H-15→L+3 (-30%), H-4→L+4 (32%) |
| 331.7 | 0.0011 | H-17→L+2 (-20%), H-16→L+3 (20%), H-12→L+3 (-19%), HOMO→L+8 (26%) |
| 330.1 | 0.0568 | H-21→L+3 (79%) |
| 329.1 | 0.0002 | H-16→L+3 (-13%), H-12→L+3 (12%), HOMO→L+8 (66%) |
| 328.2 | 0.0002 | H-21→L+1 (90%) |
| 327.3 | 0.0247 | H-6→L+4 (64%), H-5→L+4 (10%), H-3→L+5 (-14%) |
| 326.8 | 0.0032 | H-7→L+4 (-11%), H-5→L+4 (63%) |
| 326.4 | 0.1173 | H-20→L+2 (14%), H-19→L+2 (-11%), H-6→L+4 (14%), H-4→L+4 (13%), H-3→L+5 (22%) |
| 325.5 | 0.0118 | H-20→L+2 (34%), H-3→L+5 (-23%) |
| 324.8 | 0.0241 | H-20→L+2 (-19%), HOMO→L+9 (-17%), HOMO→L+10 (29%) |

# Figure S48.Bar graph (blue) reporting the position of the electronic transitions vs the oscillator strength (f) for ID-[S]-[B-Zn]-[S]-ID.The black line represents a spectrum where 1000 cm-1 is applied to each transition.

# Table S24.Atomic contributions for the frontier MO for ID-[S]-[B-Zn]-[S]-ID.

|  | H-4 | H-3 | H-2 | H-1 | H | L | L+1 | L+2 | L+3 | L+4 |
| --- | --- | --- | --- | --- | --- | --- | --- | --- | --- | --- |
| 2*Thiopene-  Benzo-porphyrins | 0.29 | 0.31 | 0.52 | 0.91 | ~1.00 | 0.38 | 0.12 | 0.70 | ~1.00 | 0.82 |
| 2*isoindigo | 0.71 | 0.69 | 0.48 | 0.09 | ~0.0 | 0.62 | 0.88 | 0.30 | ~0.0 | 0.18 |
